# Supplementary material for: Impact of pausing elective hip and knee replacement surgery during winter 2017 on subsequent service provision at a major NHS Trust: a descriptive observational study using interrupted time series
Source: BMJ Open. 2023 May 16;13(5):e066398. doi: 10.1136/bmjopen-2022-066398 (PMC10193088; doi:10.1136/bmjopen-2022-066398)
Supplement: Supplementary data [file bmjopen-2022-066398supp003.pdf]

Supplementary Table T1. OPCS-4 codes used to identify primary hip and knee replacement operations

Category	Code	Description	Notes
Primary Total Hip Replacement	W37.1	Primary total prosthetic replacement of hip joint using cement	
	W37.8	Other specified total prosthetic replacement of hip joint using cement	
	W37.9	Unspecified total prosthetic replacement of hip joint using cement	
	W38.1	Primary total prosthetic replacement of hip joint not using cement	
	W38.8	Other specified total prosthetic replacement of hip joint not using cement	
	W38.9	Unspecified total prosthetic replacement of hip joint not using cement	
	W39.1	Primary total prosthetic replacement of hip joint NEC	
	W39.8	Other specified other total prosthetic replacement of hip joint	
	W39.9	Unspecified other total prosthetic replacement of hip joint	
	W43.1	Primary total prosthetic replacement of other joint using cement NEC	
	W43.8	Other specified total prosthetic replacement of other joint using cement NEC	
	W43.9	Unspecified total prosthetic replacement of other joint using cement NEC	
	W44.1	Primary total prosthetic replacement of other joint not using cement NEC	
	W44.8	Other specified total prosthetic replacement of other joint not using cement NEC	
	W44.9	Unspecified total prosthetic replacement of other joint not using cement NEC	
	W45.1	Other primary total prosthetic replacement of other joint NEC	
	W45.8	Other specified total prosthetic replacement of other joint NEC	
	W45.9	Unspecified total prosthetic replacement of other joint NEC	
	W52.1	Primary prosthetic replacement of articulation of bone using cement NEC	

W52.8	Other specified prosthetic replacement of articulation of bone using cement NEC
W52.9	Unspecified prosthetic replacement of articulation of bone using cement NEC
W53.1	Primary prosthetic replacement of articulation of bone not using cement NEC
W53.8	Other specified prosthetic replacement of articulation of bone not using cement NEC
W53.9	Unspecified prosthetic replacement of articulation of bone not using cement NEC
W54.1	Primary prosthetic replacement of articulation of bone NEC
W54.8	Other specified prosthetic replacement of articulation of bone NEC
W54.9	Unspecified prosthetic replacement of articulation of bone NEC
W93.1	Primary hybrid prosthetic replacement of hip joint using cemented acetabular component
W93.8	Other specified hybrid prosthetic replacement of hip joint using cemented acetabular component
W93.9	Unspecified hybrid prosthetic replacement of hip joint using cemented acetabular component
W94.1	Primary hybrid prosthetic replacement of hip joint using cemented femoral component
W94.8	Other specified hybrid prosthetic replacement of hip joint using cemented femoral component
W94.9	Unspecified hybrid prosthetic replacement of hip joint using cemented femoral component
W95.1	Primary hybrid prosthetic replacement of hip joint using cement NEC
W95.8	Other specified hybrid prosthetic replacement of hip joint using cement
W95.9	Unspecified hybrid prosthetic replacement of hip joint using cement

Primary Total Knee Replacement	W40.1	Primary total prosthetic replacement of knee joint using cement	
	W40.8	Other specified total prosthetic replacement of knee joint using cement	
	W40.9	Unspecified total prosthetic replacement of knee joint using cement	
	W41.1	Primary total prosthetic replacement of knee joint not using cement	
	W41.8	Other specified total prosthetic replacement of knee joint not using cement	
	W41.9	Unspecified total prosthetic replacement of knee joint not using cement	
	W42.1	Primary total prosthetic replacement of knee joint NEC	
	W42.8	Other specified other total prosthetic replacement of knee joint	
	W42.9	Unspecified other total prosthetic replacement of knee joint	
	O18.1	Primary hybrid prosthetic replacement of knee joint using cement	
	O18.8	Other specified hybrid prosthetic replacement of knee joint using cement	
	O18.9	Unspecified hybrid prosthetic replacement of knee joint using cement	
Resurfacing / Reconstruction	W58.1	Primary resurfacing arthroplasty of joint	Require combination with site + combination codes to ID
	W58.8	Other specified reconstruction of joint	Require combination with site + combination codes to ID
	W58.9	Unspecified other reconstruction of joint	Require combination with site + combination codes to ID
Primary unicondylar / unicompartmental knee operations	W52.1	Primary prosthetic replacement of articulation of bone using cement NEC	Require combination with site + combination codes to ID
	W52.8	Other specified prosthetic replacement of articulation of other bone using cement	Require combination with site + combination codes to ID
	W52.9	Unspecified prosthetic replacement of articulation of other bone using cement	Require combination with site + combination codes to ID
	W53.1	Primary prosthetic replacement of articulation of bone not using cement NEC	Require combination with site + combination codes to ID

Jones T, *et al. BMJ Open* 2023; 13:e066398. doi: 10.1136/bmjopen-2022-066398

W53.9	Unspecified prosthetic replacement of articulation of other bone not using cement	Require combination with site + combination codes to ID
W54.0	Conversion from previous prosthetic replacement of articulation of bone NEC	Require combination with site + combination codes to ID
W54.1	Primary prosthetic replacement of articulation of bone NEC	Require combination with site + combination codes to ID
W54.8	Other specified other prosthetic replacement of articulation of other bone	Require combination with site + combination codes to ID
W54.9	Unspecified other prosthetic replacement of articulation of other bone	Require combination with site + combination codes to ID
