# Supplementary material for: Impact of pausing elective hip and knee replacement surgery during winter 2017 on subsequent service provision at a major NHS Trust: a descriptive observational study using interrupted time series
Source: BMJ Open. 2023 May 16;13(5):e066398. doi: 10.1136/bmjopen-2022-066398 (PMC10193088; doi:10.1136/bmjopen-2022-066398)
Supplement: Supplementary data [file bmjopen-2022-066398supp004.pdf]

BMJ Publishing Group Limited (BMJ) disclaims all liability and responsibility arising from any reliance placed on this supplemental material which has been supplied by the author(s)

Supplementary Table T2. Interrupted time series model results with maximum auto-correlation lag 2

	pre-trend		level change		trend change		spring		summer		autumn	
	estimate (95% CI)	p	estimate (95% CI)	p	estimate (95% CI)	p	estimate (95% CI)	p	estimate (95% CI)	p	estimate (95% CI)	p
Hip Admissions	0.995 (0.986,1.004)	0.300	1.055 (0.912,1.221)	0.469	0.991 (0.977,1.006)	0.239	1.19 (1.034,1.37)	0.015	1.207 (1.094,1.332)	0.000	1.101 (0.982,1.234)	0.099
Hip Average Age	-0.015 (-0.102,0.072)	0.737	1.571 (-0.1,3.242)	0.065	-0.06 (-0.175,0.055)	0.307	1.52 (-0.07,3.111)	0.061	2.09 (0.811,3.368)	0.001	1.031 (-0.598,2.66)	0.215
Hip Prop Women	0.999 (0.995,1.003)	0.582	0.966 (0.861,1.083)	0.549	1.007 (0.999,1.016)	0.089	1.023 (0.941,1.111)	0.596	1.088 (1.001,1.183)	0.048	0.981 (0.888,1.083)	0.700
Hip Prop 2+ Charlson	0.994 (0.982,1.007)	0.380	1.411 (1.064,1.873)	0.017	1.01 (0.994,1.027)	0.220	1.195 (0.982,1.455)	0.075	1.306 (1.096,1.557)	0.003	1.003 (0.788,1.276)	0.982
Hip Prop High Deprivation	1.003 (0.993,1.013)	0.587	1.027 (0.869,1.214)	0.754	1.004 (0.986,1.022)	0.660	0.937 (0.815,1.076)	0.358	0.877 (0.752,1.022)	0.093	0.996 (0.84,1.18)	0.963
Hip LoS	-0.006 (-0.034,0.021)	0.660	0.312 (-0.193,0.818)	0.225	-0.014 (-0.047,0.02)	0.425	0.502 (0.214,0.79)	0.001	0.135 (-0.194,0.463)	0.422	0.1 (-0.288,0.488)	0.613
Hip LoS Age 16-59	-0.003 (-0.028,0.021)	0.788	0.379 (-0.094,0.851)	0.116	-0.026 (-0.065,0.013)	0.190	0.223 (-0.156,0.602)	0.249	0.384 (0.065,0.703)	0.018	0.219 (-0.183,0.622)	0.285
Hip LoS Age 60-69	-0.004 (-0.034,0.026)	0.818	0.013 (-0.807,0.832)	0.976	0 (-0.053,0.052)	0.988	0.107 (-0.44,0.654)	0.702	0.158 (-0.468,0.783)	0.621	0.162 (-0.487,0.811)	0.625
Hip LoS Age 70-79	0.006 (-0.058,0.069)	0.862	-0.753 (-2.003,0.497)	0.238	0.007 (-0.06,0.073)	0.847	-0.433 (-1.416,0.549)	0.387	-0.842 (-1.93,0.246)	0.129	-0.865 (-1.991,0.262)	0.132
Hip LoS Age 80+	-0.068 (-0.171,0.036)	0.199	2.109 (0.683,3.535)	0.004	-0.002 (-0.128,0.123)	0.971	1.506 (0.515,2.497)	0.003	0.222 (-0.773,1.217)	0.662	1.003 (-0.229,2.235)	0.111
Hip LoS Men	-0.007 (-0.055,0.041)	0.776	0.347 (-0.583,1.278)	0.464	-0.02 (-0.083,0.043)	0.536	0.293 (-0.126,0.712)	0.170	0.289 (-0.196,0.774)	0.243	0.458 (-0.111,1.026)	0.114
Hip LoS Women	-0.004 (-0.041,0.033)	0.842	0.235 (-0.308,0.778)	0.396	-0.014 (-0.055,0.027)	0.505	0.693 (0.309,1.077)	0.000	0.009 (-0.428,0.446)	0.966	-0.203 (-0.728,0.323)	0.449
Hip LoS Charlson 0	-0.011 (-0.042,0.019)	0.469	0.297 (-0.288,0.882)	0.319	-0.005 (-0.042,0.031)	0.782	0.912 (0.549,1.276)	0.000	0.178 (-0.148,0.503)	0.285	0.222 (-0.065,0.509)	0.129
Hip LoS Charlson 1	0.034 (-0.012,0.08)	0.146	0.238 (-0.709,1.184)	0.623	-0.077 (-0.145,-0.01)	0.024	0.191 (-0.418,0.801)	0.538	0.312 (-0.405,1.029)	0.393	0.014 (-0.578,0.606)	0.963
Hip LoS Charlson 2+	-0.123 (-0.259,0.013)	0.077	0.172 (-2.224,2.569)	0.888	0.116 (-0.016,0.249)	0.085	-0.789 (-2.141,0.563)	0.253	-0.783 (-2.087,0.521)	0.239	0.188 (-2.051,2.427)	0.869
Hip LoS Dep 1	0.004 (-0.032,0.04)	0.829	0.553 (-0.391,1.496)	0.251	-0.017 (-0.068,0.035)	0.529	0.784 (0.107,1.462)	0.023	0.42 (-0.024,0.863)	0.064	0.299 (-0.358,0.956)	0.373
Hip LoS Dep 2	-0.038 (-0.083,0.006)	0.092	0.822 (-0.392,2.035)	0.184	-0.015 (-0.086,0.055)	0.670	1.509 (0.759,2.258)	0.000	0.66 (-0.246,1.567)	0.154	0.589 (-0.417,1.594)	0.251
Hip LoS Dep 3	-0.034 (-0.107,0.038)	0.353	0.617 (-0.897,2.131)	0.424	0.01 (-0.076,0.095)	0.827	-0.216 (-1.224,0.792)	0.675	-0.424 (-1.378,0.53)	0.384	-0.701 (-1.536,0.135)	0.100
Hip LoS Dep 4	0.081 (-0.005,0.166)	0.064	-0.473 (-2.181,1.236)	0.588	-0.112 (-0.215,-0.009)	0.034	-0.374 (-1.65,0.903)	0.566	-1.204 (-2.351,-0.057)	0.040	-0.491 (-1.918,0.935)	0.500
Hip LoS Dep 5	0.015 (-0.086,0.116)	0.773	-0.72 (-2.059,0.62)	0.292	0 (-0.098,0.099)	0.999	0.621 (-0.084,1.327)	0.084	0.689 (-0.31,1.689)	0.176	0.816 (-0.784,2.416)	0.317
Hip Bed Occ	0.997 (0.987,1.008)	0.643	1 (0.844,1.186)	0.997	0.987 (0.97,1.005)	0.149	1.291 (1.11,1.501)	0.001	1.283 (1.125,1.463)	0.000	1.146 (0.988,1.328)	0.071
Hip Public Private	0.013 (-0.015,0.041)	0.377	-0.741 (-1.237,-0.245)	0.003	-0.019 (-0.05,0.011)	0.218	-0.008 (-0.212,0.196)	0.939	0.308 (0.154,0.463)	0.000	0.038 (-0.166,0.241)	0.718
Knee Admissions	0.995 (0.99,1.001)	0.106	0.843 (0.728,0.976)	0.022	1.005 (0.996,1.014)	0.256	1.308 (1.157,1.479)	0.000	1.26 (1.138,1.396)	0.000	1.286 (1.164,1.42)	0.000
Knee Average Age	-0.078 (-0.157,0.001)	0.054	-1.632 (-2.988,-0.276)	0.018	0.211 (0.117,0.305)	0.000	0.926 (-0.112,1.965)	0.080	0.953 (-0.021,1.927)	0.055	0.354 (-0.531,1.24)	0.433
Knee Prop Women	1.004 (0.998,1.01)	0.150	0.96 (0.85,1.084)	0.513	0.994 (0.986,1.003)	0.193	1.037 (0.963,1.117)	0.336	1.017 (0.924,1.118)	0.735	1.036 (0.958,1.12)	0.375
Knee Prop 2+ Charlson	1.009 (0.993,1.026)	0.249	0.638 (0.455,0.894)	0.009	1.042 (1.017,1.067)	0.001	1.156 (0.911,1.467)	0.234	1.074 (0.849,1.359)	0.551	0.909 (0.628,1.315)	0.612
Knee Prop High Deprivation	1.005 (0.997,1.013)	0.189	0.968 (0.786,1.191)	0.758	0.986 (0.974,0.998)	0.021	1.224 (1.077,1.39)	0.002	1.075 (0.942,1.227)	0.282	1.031 (0.902,1.178)	0.656
Knee LoS	-0.024 (-0.049,0.001)	0.058	0.176 (-0.279,0.63)	0.449	-0.008 (-0.036,0.02)	0.566	0.422 (0.073,0.771)	0.018	0.15 (-0.166,0.467)	0.352	0.396 (0.015,0.777)	0.042

Jones T, *et al. BMJ Open* 2023; 13:e066398. doi: 10.1136/bmjopen-2022-066398

Knee LoS Age 16-59	-0.016 (-0.037,0.006)	0.151	0.403 (-0.045,0.852)	0.078	-0.028 (-0.06,0.005)	0.096	0.484 (0.13,0.839)	0.007	0.422 (-0.021,0.866)	0.062	0.475 (0.19,0.76)	0.001
Knee LoS Age 16-59	0.007 (-0.028,0.043)	0.684	-0.295 (-1.015,0.426)	0.423	-0.026 (-0.072,0.02)	0.262	0.069 (-0.46,0.599)	0.797	-0.194 (-0.687,0.3)	0.441	-0.096 (-0.725,0.532)	0.764
Knee LoS Age 70-79	-0.009 (-0.057,0.038)	0.705	0.77 (0.002,1.538)	0.049	-0.054 (-0.11,0.003)	0.061	-0.086 (-0.715,0.542)	0.787	-0.552 (-1.163,0.059)	0.077	0.367 (-0.352,1.085)	0.317
Knee LoS Age 80+	-0.051 (-0.118,0.015)	0.131	-0.562 (-2.298,1.173)	0.525	0.028 (-0.081,0.137)	0.612	1.521 (0.03,3.011)	0.046	1.37 (0.169,2.57)	0.025	1.361 (0.496,2.227)	0.002
Knee LoS Men	-0.007 (-0.031,0.018)	0.581	0.096 (-0.405,0.597)	0.708	-0.033 (-0.068,0.002)	0.064	0.082 (-0.367,0.531)	0.720	0.157 (-0.262,0.575)	0.464	0.205 (-0.221,0.631)	0.346
Knee LoS Women	-0.042 (-0.084,0)	0.052	0.252 (-0.461,0.966)	0.488	0.017 (-0.029,0.063)	0.478	0.635 (0.048,1.223)	0.034	0.15 (-0.397,0.698)	0.590	0.49 (-0.17,1.151)	0.146
Knee LoS Charlson 0	-0.021 (-0.046,0.004)	0.095	0.067 (-0.362,0.495)	0.760	-0.011 (-0.045,0.024)	0.545	0.645 (0.334,0.956)	0.000	0.193 (-0.111,0.497)	0.213	0.415 (0.069,0.76)	0.019
Knee LoS Charlson 1	-0.03 (-0.074,0.015)	0.191	0.47 (-0.391,1.331)	0.284	-0.029 (-0.083,0.025)	0.298	0.455 (-0.043,0.953)	0.073	0.324 (-0.297,0.945)	0.307	1.058 (0.367,1.748)	0.003
Knee LoS Charlson 2+	-0.04 (-0.107,0.028)	0.247	0.407 (-2.28,3.095)	0.766	-0.022 (-0.196,0.151)	0.802	-0.627 (-1.698,0.443)	0.251	-0.949 (-2.342,0.444)	0.182	-1.433 (-2.229,-0.637)	0.000
Knee LoS Dep 1	-0.009 (-0.06,0.041)	0.712	-0.035 (-0.726,0.656)	0.920	-0.008 (-0.073,0.056)	0.800	0.257 (-0.47,0.985)	0.488	0.096 (-0.619,0.811)	0.793	-0.127 (-0.725,0.472)	0.678
Knee LoS Dep 2	-0.019 (-0.06,0.022)	0.358	0.199 (-0.699,1.097)	0.664	-0.043 (-0.098,0.012)	0.124	0.018 (-0.776,0.812)	0.965	-0.731 (-1.49,0.029)	0.059	0.05 (-0.822,0.922)	0.911
Knee LoS Dep 3	0.014 (-0.036,0.064)	0.586	-0.426 (-1.355,0.503)	0.369	-0.036 (-0.106,0.033)	0.302	0.976 (0.061,1.892)	0.036	0.909 (-0.034,1.852)	0.059	0.547 (-0.217,1.312)	0.160
Knee LoS Dep 4	-0.064 (-0.123,-0.004)	0.035	0.634 (-0.293,1.56)	0.180	0.033 (-0.039,0.105)	0.373	0.669 (-0.187,1.525)	0.126	0.617 (0.02,1.214)	0.043	0.852 (-0.208,1.912)	0.115
Knee LoS Dep 5	-0.035 (-0.09,0.021)	0.224	0.709 (-0.303,1.721)	0.170	0.015 (-0.046,0.076)	0.628	0.123 (-0.73,0.976)	0.778	-0.387 (-1.281,0.507)	0.397	1.104 (0.077,2.131)	0.035
Knee Bed Occ	0.993 (0.984,1.002)	0.103	0.834 (0.704,0.989)	0.037	1 (0.989,1.011)	0.993	1.42 (1.297,1.556)	0.000	1.373 (1.218,1.547)	0.000	1.465 (1.312,1.635)	0.000
Knee Public Private	0.006 (-0.02,0.031)	0.667	-0.476 (-1.026,0.074)	0.090	-0.015 (-0.04,0.009)	0.225	0.113 (-0.089,0.314)	0.274	0.276 (0.035,0.517)	0.025	0.131 (-0.071,0.332)	0.205
Elec Emerg Ratio	-0.005 (-0.013,0.002)	0.171	-0.322 (-0.446,-0.198)	0.000	-0.016 (-0.026,-0.005)	0.003	-0.008 (-0.12,0.103)	0.886	-0.053 (-0.182,0.077)	0.424	-0.028 (-0.151,0.096)	0.661

Jones T, *et al. BMJ Open* 2023; 13:e066398. doi: 10.1136/bmjopen-2022-066398
