# Supplementary material for: Impact of pausing elective hip and knee replacement surgery during winter 2017 on subsequent service provision at a major NHS Trust: a descriptive observational study using interrupted time series
Source: BMJ Open. 2023 May 16;13(5):e066398. doi: 10.1136/bmjopen-2022-066398 (PMC10193088; doi:10.1136/bmjopen-2022-066398)
Supplement: Supplementary data [file bmjopen-2022-066398supp001.pdf]

Supplementary Figure F1. Ratio of elective to emergency hospital admissions for any reason at the Trust

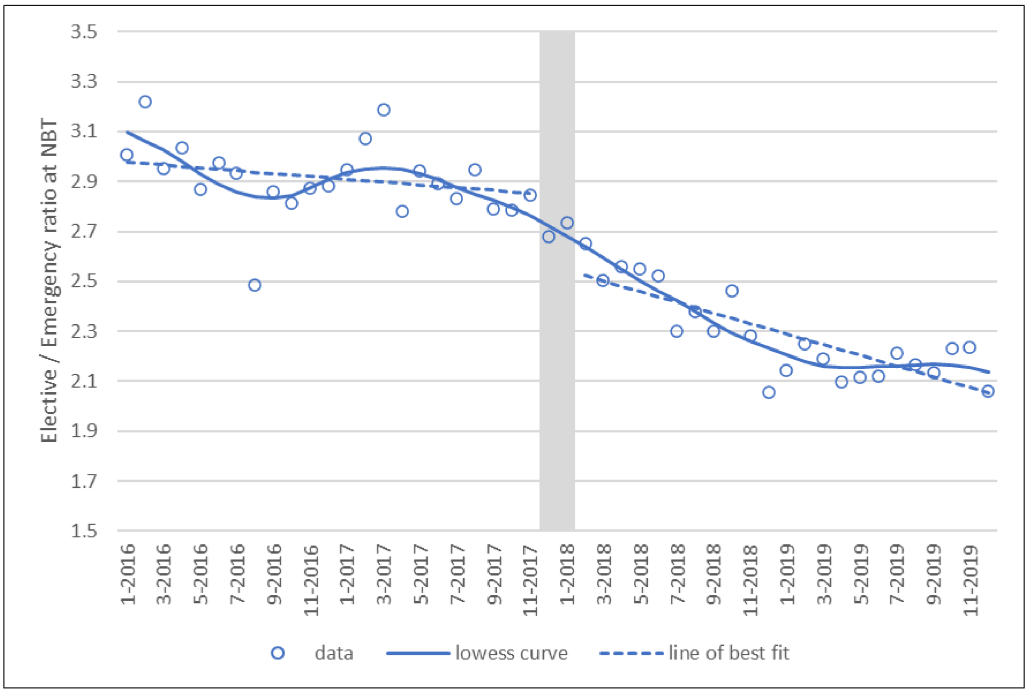

Note: grey area shows the winter 2017 cancellations and is excluded from the analysis
