# Supplementary material for: Impact of pausing elective hip and knee replacement surgery during winter 2017 on subsequent service provision at a major NHS Trust: a descriptive observational study using interrupted time series
Source: BMJ Open. 2023 May 16;13(5):e066398. doi: 10.1136/bmjopen-2022-066398 (PMC10193088; doi:10.1136/bmjopen-2022-066398)
Supplement: Supplementary data [file bmjopen-2022-066398supp005.pdf]

Supplementary Table T3. Interrupted time series model results with maximum auto-correlation lag 0

	pre-trend		level change		trend change		spring		summer		autumn	
	estimate (95% CI)	p	estimate (95% CI)	p	estimate (95% CI)	p	estimate (95% CI)	p	estimate (95% CI)	p	estimate (95% CI)	p
Hip Admissions	0.995 (0.985,1.005)	0.323	1.055 (0.902,1.235)	0.503	0.991 (0.978,1.005)	0.220	1.19 (1.036,1.367)	0.014	1.207 (1.088,1.339)	0.000	1.101 (0.983,1.234)	0.098
Hip Average Age	-0.015 (-0.134,0.104)	0.806	1.571 (-0.863,4.004)	0.206	-0.06 (-0.208,0.088)	0.427	1.52 (-0.079,3.119)	0.062	2.09 (0.685,3.494)	0.004	1.031 (-0.505,2.567)	0.188
Hip Prop Women	0.999 (0.993,1.004)	0.674	0.966 (0.826,1.13)	0.662	1.007 (0.997,1.017)	0.158	1.023 (0.937,1.116)	0.617	1.088 (0.995,1.189)	0.064	0.981 (0.878,1.096)	0.730
Hip Prop 2+ Charlson	0.994 (0.98,1.009)	0.455	1.411 (0.969,2.055)	0.072	1.01 (0.989,1.031)	0.344	1.195 (0.971,1.471)	0.092	1.306 (1.043,1.637)	0.020	1.003 (0.734,1.37)	0.986
Hip Prop High Deprivation	1.003 (0.991,1.014)	0.631	1.027 (0.846,1.247)	0.787	1.004 (0.983,1.025)	0.704	0.937 (0.819,1.072)	0.342	0.877 (0.746,1.03)	0.110	0.996 (0.831,1.193)	0.965
Hip LoS	-0.006 (-0.038,0.026)	0.704	0.312 (-0.262,0.887)	0.287	-0.014 (-0.058,0.03)	0.542	0.502 (0.132,0.872)	0.008	0.135 (-0.292,0.562)	0.537	0.1 (-0.287,0.487)	0.612
Hip LoS Age 16-59	-0.003 (-0.033,0.026)	0.821	0.379 (-0.165,0.922)	0.172	-0.026 (-0.067,0.015)	0.210	0.223 (-0.17,0.617)	0.266	0.384 (0.053,0.715)	0.023	0.219 (-0.168,0.607)	0.267
Hip LoS Age 60-69	-0.004 (-0.046,0.039)	0.871	0.013 (-1.026,1.052)	0.981	0 (-0.069,0.068)	0.990	0.107 (-0.592,0.805)	0.765	0.158 (-0.514,0.829)	0.645	0.162 (-0.588,0.912)	0.672
Hip LoS Age 70-79	0.006 (-0.067,0.078)	0.879	-0.753 (-2.031,0.525)	0.248	0.007 (-0.069,0.082)	0.865	-0.433 (-1.583,0.716)	0.460	-0.842 (-2.028,0.344)	0.164	-0.865 (-2.164,0.435)	0.192
Hip LoS Age 80+	-0.068 (-0.193,0.058)	0.291	2.109 (0.536,3.682)	0.009	-0.002 (-0.16,0.155)	0.977	1.506 (-0.007,3.018)	0.051	0.222 (-1.036,1.48)	0.729	1.003 (-0.375,2.38)	0.154
Hip LoS Men	-0.007 (-0.056,0.042)	0.783	0.347 (-0.58,1.275)	0.463	-0.02 (-0.084,0.044)	0.543	0.293 (-0.283,0.87)	0.319	0.289 (-0.314,0.892)	0.347	0.458 (-0.15,1.066)	0.140
Hip LoS Women	-0.004 (-0.048,0.041)	0.868	0.235 (-0.563,1.033)	0.564	-0.014 (-0.071,0.043)	0.634	0.693 (0.164,1.222)	0.010	0.009 (-0.543,0.562)	0.973	-0.203 (-0.727,0.322)	0.448
Hip LoS Charlson 0	-0.011 (-0.047,0.024)	0.532	0.297 (-0.428,1.023)	0.422	-0.005 (-0.049,0.039)	0.817	0.912 (0.522,1.302)	0.000	0.178 (-0.232,0.587)	0.396	0.222 (-0.135,0.579)	0.223
Hip LoS Charlson 1	0.034 (-0.023,0.091)	0.240	0.238 (-0.704,1.179)	0.621	-0.077 (-0.157,0.002)	0.056	0.191 (-0.562,0.945)	0.619	0.312 (-0.397,1.022)	0.388	0.014 (-0.544,0.572)	0.960
Hip LoS Charlson 2+	-0.123 (-0.266,0.02)	0.092	0.172 (-2.155,2.5)	0.885	0.116 (-0.04,0.273)	0.144	-0.789 (-2.218,0.64)	0.279	-0.783 (-2.108,0.541)	0.246	0.188 (-1.779,2.156)	0.851
Hip LoS Dep 1	0.004 (-0.039,0.047)	0.857	0.553 (-0.509,1.614)	0.307	-0.017 (-0.078,0.045)	0.599	0.784 (0.118,1.451)	0.021	0.42 (-0.084,0.923)	0.102	0.299 (-0.356,0.954)	0.371
Hip LoS Dep 2	-0.038 (-0.087,0.01)	0.124	0.822 (-0.528,2.172)	0.233	-0.015 (-0.103,0.072)	0.730	1.509 (0.797,2.22)	0.000	0.66 (-0.225,1.546)	0.144	0.589 (-0.26,1.437)	0.174
Hip LoS Dep 3	-0.034 (-0.096,0.027)	0.275	0.617 (-1.066,2.3)	0.472	0.01 (-0.09,0.109)	0.851	-0.216 (-1.343,0.912)	0.708	-0.424 (-1.475,0.627)	0.429	-0.701 (-1.667,0.266)	0.155
Hip LoS Dep 4	0.081 (-0.023,0.185)	0.129	-0.473 (-2.643,1.698)	0.670	-0.112 (-0.258,0.034)	0.134	-0.374 (-1.695,0.947)	0.579	-1.204 (-2.409,0.001)	0.050	-0.491 (-1.978,0.995)	0.517
Hip LoS Dep 5	0.015 (-0.112,0.142)	0.819	-0.72 (-2.66,1.221)	0.467	0 (-0.135,0.135)	0.999	0.621 (-0.412,1.655)	0.239	0.689 (-0.485,1.864)	0.250	0.816 (-0.891,2.524)	0.349
Hip Bed Occ	0.997 (0.987,1.009)	0.653	1 (0.824,1.215)	0.997	0.987 (0.972,1.002)	0.097	1.291 (1.125,1.48)	0.000	1.283 (1.129,1.458)	0.000	1.146 (0.984,1.334)	0.080
Hip Public Private	0.013 (-0.015,0.041)	0.377	-0.741 (-1.237,-0.245)	0.003	-0.019 (-0.05,0.011)	0.218	-0.008 (-0.212,0.196)	0.939	0.308 (0.154,0.463)	0.000	0.038 (-0.166,0.241)	0.718
Knee Admissions	0.995 (0.987,1.004)	0.274	0.843 (0.702,1.013)	0.068	1.005 (0.993,1.017)	0.422	1.308 (1.154,1.483)	0.000	1.26 (1.114,1.426)	0.000	1.286 (1.147,1.441)	0.000
Knee Average Age	-0.078 (-0.157,0.001)	0.053	-1.632 (-3.299,0.035)	0.055	0.211 (0.102,0.32)	0.000	0.926 (-0.129,1.981)	0.085	0.953 (-0.257,2.163)	0.123	0.354 (-0.777,1.485)	0.540
Knee Prop Women	1.004 (0.998,1.011)	0.182	0.96 (0.84,1.097)	0.551	0.994 (0.986,1.003)	0.184	1.037 (0.939,1.146)	0.474	1.017 (0.908,1.138)	0.776	1.036 (0.928,1.157)	0.529
Knee Prop 2+ Charlson	1.009 (0.988,1.031)	0.384	0.638 (0.392,1.037)	0.070	1.042 (1.008,1.077)	0.015	1.156 (0.891,1.501)	0.276	1.074 (0.829,1.393)	0.589	0.909 (0.636,1.299)	0.600
Knee Prop High Deprivation	1.005 (0.997,1.014)	0.237	0.968 (0.784,1.195)	0.761	0.986 (0.974,0.998)	0.023	1.224 (1.099,1.363)	0.000	1.075 (0.956,1.209)	0.225	1.031 (0.911,1.166)	0.631
Knee LoS	-0.024 (-0.052,0.004)	0.089	0.176 (-0.352,0.703)	0.515	-0.008 (-0.041,0.025)	0.625	0.422 (0.117,0.726)	0.007	0.15 (-0.174,0.475)	0.364	0.396 (0.027,0.765)	0.036

Knee LoS Age 16-59	-0.016 (-0.048,0.017)	0.352	0.403 (-0.286,1.093)	0.252	-0.028 (-0.074,0.019)	0.246	0.484 (0.111,0.857)	0.011	0.422 (-0.111,0.956)	0.121	0.475 (0.056,0.893)	0.026
Knee LoS Age 16-59	0.007 (-0.026,0.041)	0.664	-0.295 (-1.22,0.63)	0.532	-0.026 (-0.085,0.033)	0.384	0.069 (-0.465,0.604)	0.799	-0.194 (-0.754,0.366)	0.497	-0.096 (-0.673,0.48)	0.743
Knee LoS Age 70-79	-0.009 (-0.072,0.054)	0.775	0.77 (-0.267,1.807)	0.145	-0.054 (-0.128,0.02)	0.154	-0.086 (-0.782,0.609)	0.808	-0.552 (-1.267,0.163)	0.130	0.367 (-0.528,1.261)	0.421
Knee LoS Age 80+	-0.051 (-0.154,0.051)	0.327	-0.562 (-2.568,1.443)	0.583	0.028 (-0.105,0.161)	0.678	1.521 (-0.076,3.118)	0.062	1.37 (-0.178,2.918)	0.083	1.361 (-0.071,2.794)	0.063
Knee LoS Men	-0.007 (-0.037,0.023)	0.656	0.096 (-0.512,0.704)	0.758	-0.033 (-0.075,0.009)	0.121	0.082 (-0.382,0.547)	0.729	0.157 (-0.272,0.585)	0.474	0.205 (-0.265,0.675)	0.392
Knee LoS Women	-0.042 (-0.088,0.004)	0.071	0.252 (-0.495,1)	0.508	0.017 (-0.036,0.07)	0.536	0.635 (0.107,1.164)	0.019	0.15 (-0.365,0.666)	0.567	0.49 (-0.022,1.003)	0.061
Knee LoS Charlson 0	-0.021 (-0.054,0.012)	0.214	0.067 (-0.49,0.624)	0.814	-0.011 (-0.056,0.034)	0.643	0.645 (0.278,1.013)	0.001	0.193 (-0.146,0.533)	0.265	0.415 (-0.078,0.907)	0.099
Knee LoS Charlson 1	-0.03 (-0.081,0.022)	0.260	0.47 (-0.741,1.681)	0.447	-0.029 (-0.093,0.036)	0.384	0.455 (-0.111,1.021)	0.115	0.324 (-0.426,1.074)	0.398	1.058 (0.262,1.854)	0.009
Knee LoS Charlson 2+	-0.04 (-0.111,0.032)	0.276	0.407 (-2.137,2.951)	0.754	-0.022 (-0.184,0.14)	0.788	-0.627 (-1.883,0.628)	0.327	-0.949 (-2.145,0.247)	0.120	-1.433 (-2.278,-0.589)	0.001
Knee LoS Dep 1	-0.009 (-0.064,0.045)	0.733	-0.035 (-0.918,0.847)	0.937	-0.008 (-0.082,0.066)	0.826	0.257 (-0.443,0.958)	0.471	0.096 (-0.699,0.89)	0.813	-0.127 (-0.786,0.532)	0.706
Knee LoS Dep 2	-0.019 (-0.065,0.026)	0.413	0.199 (-1.031,1.429)	0.751	-0.043 (-0.116,0.031)	0.253	0.018 (-0.822,0.858)	0.967	-0.731 (-1.538,0.077)	0.076	0.05 (-0.819,0.919)	0.910
Knee LoS Dep 3	0.014 (-0.05,0.078)	0.670	-0.426 (-1.892,1.04)	0.569	-0.036 (-0.116,0.043)	0.369	0.976 (-0.09,2.043)	0.073	0.909 (-0.134,1.952)	0.088	0.547 (-0.408,1.503)	0.261
Knee LoS Dep 4	-0.064 (-0.143,0.015)	0.113	0.634 (-0.591,1.858)	0.310	0.033 (-0.057,0.123)	0.474	0.669 (-0.193,1.531)	0.128	0.617 (-0.032,1.266)	0.063	0.852 (-0.138,1.842)	0.092
Knee LoS Dep 5	-0.035 (-0.116,0.047)	0.405	0.709 (-0.701,2.119)	0.324	0.015 (-0.076,0.106)	0.745	0.123 (-0.882,1.128)	0.810	-0.387 (-1.379,0.606)	0.445	1.104 (-0.268,2.476)	0.115
Knee Bed Occ	0.993 (0.984,1.001)	0.074	0.834 (0.711,0.979)	0.027	1 (0.989,1.011)	0.993	1.42 (1.269,1.59)	0.000	1.373 (1.217,1.548)	0.000	1.465 (1.287,1.667)	0.000
Knee Public Private	0.006 (-0.02,0.031)	0.667	-0.476 (-1.026,0.074)	0.090	-0.015 (-0.04,0.009)	0.225	0.113 (-0.089,0.314)	0.274	0.276 (0.035,0.517)	0.025	0.131 (-0.071,0.332)	0.205
Elec Emerg Ratio	-0.005 (-0.013,0.002)	0.167	-0.322 (-0.451,-0.192)	0.000	-0.016 (-0.025,-0.006)	0.001	-0.008 (-0.117,0.101)	0.884	-0.053 (-0.174,0.068)	0.392	-0.028 (-0.128,0.073)	0.591

Jones T, *et al. BMJ Open* 2023; 13:e066398. doi: 10.1136/bmjopen-2022-066398
