# Supplementary material for: Impact of pausing elective hip and knee replacement surgery during winter 2017 on subsequent service provision at a major NHS Trust: a descriptive observational study using interrupted time series
Source: BMJ Open. 2023 May 16;13(5):e066398. doi: 10.1136/bmjopen-2022-066398 (PMC10193088; doi:10.1136/bmjopen-2022-066398)
Supplement: Supplementary data [file bmjopen-2022-066398supp006.pdf]

Supplementary Table T4. Interrupted time series model results with maximum auto-correlation lag 5

	pre-trend		level change		trend change		spring		summer		autumn	
	estimate (95% CI)	p	estimate (95% CI)	p	estimate (95% CI)	p	estimate (95% CI)	p	estimate (95% CI)	p	estimate (95% CI)	p
Hip Admissions	0.995 (0.987,1.003)	0.249	1.055 (0.917,1.215)	0.454	0.991 (0.978,1.005)	0.217	1.19 (1.04,1.362)	0.012	1.207 (1.104,1.32)	0.000	1.101 (0.993,1.221)	0.069
Hip Average Age	-0.015 (-0.094,0.065)	0.714	1.571 (0.241,2.901)	0.021	-0.06 (-0.168,0.048)	0.277	1.52 (-0.178,3.218)	0.079	2.09 (0.818,3.361)	0.001	1.031 (-0.639,2.701)	0.226
Hip Prop Women	0.999 (0.995,1.002)	0.503	0.966 (0.895,1.042)	0.367	1.007 (1.001,1.014)	0.027	1.023 (0.932,1.122)	0.636	1.088 (1,1.184)	0.051	0.981 (0.879,1.094)	0.727
Hip Prop 2+ Charlson	0.994 (0.985,1.004)	0.261	1.411 (1.116,1.785)	0.004	1.01 (1,1.02)	0.042	1.195 (0.979,1.459)	0.079	1.306 (1.111,1.535)	0.001	1.003 (0.792,1.27)	0.981
Hip Prop High Deprivation	1.003 (0.994,1.012)	0.551	1.027 (0.883,1.195)	0.729	1.004 (0.99,1.018)	0.577	0.937 (0.805,1.091)	0.400	0.877 (0.752,1.022)	0.092	0.996 (0.847,1.171)	0.961
Hip LoS	-0.006 (-0.029,0.017)	0.605	0.312 (-0.12,0.745)	0.157	-0.014 (-0.042,0.015)	0.346	0.502 (0.286,0.718)	0.000	0.135 (-0.191,0.461)	0.418	0.1 (-0.332,0.532)	0.650
Hip LoS Age 16-59	-0.003 (-0.026,0.02)	0.773	0.379 (-0.046,0.803)	0.080	-0.026 (-0.062,0.01)	0.152	0.223 (-0.113,0.559)	0.193	0.384 (0.09,0.679)	0.011	0.219 (-0.191,0.63)	0.295
Hip LoS Age 60-69	-0.004 (-0.031,0.024)	0.799	0.013 (-0.766,0.791)	0.975	0 (-0.051,0.05)	0.987	0.107 (-0.33,0.543)	0.632	0.158 (-0.451,0.766)	0.611	0.162 (-0.352,0.676)	0.537
Hip LoS Age 70-79	0.006 (-0.054,0.065)	0.853	-0.753 (-1.944,0.438)	0.215	0.007 (-0.056,0.069)	0.836	-0.433 (-1.343,0.476)	0.350	-0.842 (-1.91,0.226)	0.122	-0.865 (-2.063,0.334)	0.157
Hip LoS Age 80+	-0.068 (-0.147,0.012)	0.097	2.109 (0.803,3.414)	0.002	-0.002 (-0.111,0.107)	0.967	1.506 (0.499,2.513)	0.003	0.222 (-0.799,1.243)	0.670	1.003 (-0.111,2.116)	0.078
Hip LoS Men	-0.007 (-0.047,0.033)	0.732	0.347 (-0.44,1.135)	0.387	-0.02 (-0.073,0.033)	0.458	0.293 (-0.117,0.704)	0.161	0.289 (-0.193,0.771)	0.239	0.458 (-0.163,1.079)	0.149
Hip LoS Women	-0.004 (-0.035,0.028)	0.816	0.235 (-0.271,0.741)	0.362	-0.014 (-0.046,0.018)	0.391	0.693 (0.395,0.991)	0.000	0.009 (-0.421,0.439)	0.966	-0.203 (-0.762,0.357)	0.477
Hip LoS Charlson 0	-0.011 (-0.039,0.016)	0.418	0.297 (-0.242,0.837)	0.280	-0.005 (-0.035,0.025)	0.738	0.912 (0.617,1.208)	0.000	0.178 (-0.123,0.478)	0.247	0.222 (-0.066,0.51)	0.131
Hip LoS Charlson 1	0.034 (0.003,0.065)	0.030	0.238 (-0.604,1.08)	0.580	-0.077 (-0.137,-0.018)	0.011	0.191 (-0.385,0.767)	0.515	0.312 (-0.431,1.056)	0.410	0.014 (-0.563,0.591)	0.962
Hip LoS Charlson 2+	-0.123 (-0.239,-0.007)	0.038	0.172 (-1.97,2.314)	0.875	0.116 (0.005,0.228)	0.040	-0.789 (-2.189,0.611)	0.269	-0.783 (-2.119,0.553)	0.250	0.188 (-2.176,2.552)	0.876
Hip LoS Dep 1	0.004 (-0.029,0.037)	0.814	0.553 (-0.303,1.408)	0.206	-0.017 (-0.065,0.032)	0.501	0.784 (0.131,1.437)	0.019	0.42 (-0.034,0.874)	0.070	0.299 (-0.401,0.999)	0.402
Hip LoS Dep 2	-0.038 (-0.083,0.006)	0.092	0.822 (-0.167,1.81)	0.103	-0.015 (-0.078,0.047)	0.630	1.509 (0.737,2.28)	0.000	0.66 (-0.202,1.522)	0.133	0.589 (-0.461,1.638)	0.272
Hip LoS Dep 3	-0.034 (-0.102,0.033)	0.319	0.617 (-0.789,2.023)	0.390	0.01 (-0.072,0.091)	0.819	-0.216 (-1.011,0.579)	0.595	-0.424 (-1.293,0.445)	0.339	-0.701 (-1.351,-0.05)	0.035
Hip LoS Dep 4	0.081 (0.017,0.144)	0.013	-0.473 (-1.858,0.913)	0.504	-0.112 (-0.189,-0.035)	0.004	-0.374 (-1.59,0.842)	0.547	-1.204 (-2.355,-0.053)	0.040	-0.491 (-1.989,1.007)	0.520
Hip LoS Dep 5	0.015 (-0.056,0.085)	0.682	-0.72 (-1.647,0.208)	0.128	0 (-0.07,0.07)	0.998	0.621 (-0.104,1.347)	0.093	0.689 (-0.25,1.629)	0.150	0.816 (-0.696,2.329)	0.290
Hip Bed Occ	0.997 (0.987,1.008)	0.627	1 (0.862,1.161)	0.996	0.987 (0.97,1.004)	0.128	1.291 (1.116,1.492)	0.001	1.283 (1.14,1.444)	0.000	1.146 (0.99,1.326)	0.068
Hip Public Private	0.013 (-0.015,0.041)	0.377	-0.741 (-1.237,-0.245)	0.003	-0.019 (-0.05,0.011)	0.218	-0.008 (-0.212,0.196)	0.939	0.308 (0.154,0.463)	0.000	0.038 (-0.166,0.241)	0.718
Knee Admissions	0.995 (0.992,0.999)	0.016	0.843 (0.761,0.934)	0.001	1.005 (0.998,1.012)	0.170	1.308 (1.154,1.482)	0.000	1.26 (1.16,1.369)	0.000	1.286 (1.178,1.403)	0.000
Knee Average Age	-0.078 (-0.152,-0.005)	0.037	-1.632 (-2.825,-0.439)	0.007	0.211 (0.128,0.293)	0.000	0.926 (-0.072,1.924)	0.069	0.953 (0.077,1.829)	0.033	0.354 (-0.422,1.13)	0.371
Knee Prop Women	1.004 (0.999,1.009)	0.086	0.96 (0.861,1.071)	0.465	0.994 (0.987,1.001)	0.097	1.037 (0.973,1.105)	0.260	1.017 (0.922,1.121)	0.743	1.036 (0.966,1.111)	0.319
Knee Prop 2+ Charlson	1.009 (0.993,1.026)	0.250	0.638 (0.468,0.869)	0.004	1.042 (1.021,1.064)	0.000	1.156 (0.931,1.436)	0.190	1.074 (0.841,1.372)	0.567	0.909 (0.626,1.321)	0.617
Knee Prop High Deprivation	1.005 (0.999,1.011)	0.107	0.968 (0.835,1.122)	0.664	0.986 (0.977,0.995)	0.003	1.224 (1.082,1.384)	0.001	1.075 (0.95,1.217)	0.251	1.031 (0.924,1.149)	0.586
Knee LoS	-0.024 (-0.047,-0.001)	0.042	0.176 (-0.2,0.551)	0.360	-0.008 (-0.031,0.015)	0.490	0.422 (0.033,0.81)	0.033	0.15 (-0.199,0.499)	0.398	0.396 (-0.011,0.803)	0.057

Jones T, *et al. BMJ Open* 2023; 13:e066398. doi: 10.1136/bmjopen-2022-066398

Knee LoS Age 16-59	-0.016 (-0.032,0.001)	0.068	0.403 (0.008,0.799)	0.046	-0.028 (-0.059,0.003)	0.081	0.484 (0.198,0.77)	0.001	0.422 (-0.01,0.855)	0.056	0.475 (0.216,0.734)	0.000
Knee LoS Age 16-59	0.007 (-0.018,0.033)	0.571	-0.295 (-0.854,0.265)	0.302	-0.026 (-0.062,0.009)	0.147	0.069 (-0.455,0.593)	0.795	-0.194 (-0.652,0.265)	0.407	-0.096 (-0.713,0.52)	0.759
Knee LoS Age 70-79	-0.009 (-0.045,0.027)	0.616	0.77 (0.101,1.439)	0.024	-0.054 (-0.096,-0.012)	0.012	-0.086 (-0.736,0.563)	0.794	-0.552 (-1.116,0.012)	0.055	0.367 (-0.3,1.034)	0.281
Knee LoS Age 80+	-0.051 (-0.097,-0.006)	0.026	-0.562 (-1.849,0.724)	0.391	0.028 (-0.062,0.119)	0.541	1.521 (0.119,2.923)	0.034	1.37 (0.391,2.349)	0.006	1.361 (0.617,2.106)	0.000
Knee LoS Men	-0.007 (-0.028,0.014)	0.514	0.096 (-0.297,0.488)	0.633	-0.033 (-0.062,-0.004)	0.024	0.082 (-0.416,0.58)	0.746	0.157 (-0.244,0.558)	0.444	0.205 (-0.149,0.559)	0.256
Knee LoS Women	-0.042 (-0.079,-0.005)	0.027	0.252 (-0.356,0.861)	0.416	0.017 (-0.018,0.052)	0.350	0.635 (-0.036,1.307)	0.064	0.15 (-0.454,0.754)	0.625	0.49 (-0.258,1.239)	0.199
Knee LoS Charlson 0	-0.021 (-0.044,0.002)	0.077	0.067 (-0.312,0.446)	0.730	-0.011 (-0.036,0.015)	0.419	0.645 (0.331,0.959)	0.000	0.193 (-0.129,0.516)	0.240	0.415 (0.075,0.754)	0.017
Knee LoS Charlson 1	-0.03 (-0.064,0.005)	0.092	0.47 (-0.199,1.139)	0.169	-0.029 (-0.067,0.01)	0.145	0.455 (-0.097,1.007)	0.106	0.324 (-0.341,0.988)	0.340	1.058 (0.384,1.731)	0.002
Knee LoS Charlson 2+	-0.04 (-0.092,0.012)	0.134	0.407 (-1.956,2.771)	0.736	-0.022 (-0.184,0.14)	0.789	-0.627 (-1.625,0.37)	0.218	-0.949 (-2.374,0.476)	0.192	-1.433 (-2.175,-0.691)	0.000
Knee LoS Dep 1	-0.009 (-0.05,0.031)	0.644	-0.035 (-0.656,0.586)	0.911	-0.008 (-0.059,0.043)	0.750	0.257 (-0.541,1.056)	0.527	0.096 (-0.581,0.772)	0.782	-0.127 (-0.724,0.47)	0.677
Knee LoS Dep 2	-0.019 (-0.055,0.017)	0.300	0.199 (-0.57,0.968)	0.612	-0.043 (-0.086,0)	0.049	0.018 (-0.731,0.767)	0.962	-0.731 (-1.507,0.046)	0.065	0.05 (-0.887,0.987)	0.917
Knee LoS Dep 3	0.014 (-0.031,0.059)	0.544	-0.426 (-1.102,0.25)	0.217	-0.036 (-0.095,0.022)	0.220	0.976 (0.281,1.672)	0.006	0.909 (-0.067,1.886)	0.068	0.547 (-0.12,1.215)	0.108
Knee LoS Dep 4	-0.064 (-0.127,-0.001)	0.047	0.634 (-0.165,1.432)	0.120	0.033 (-0.04,0.106)	0.380	0.669 (-0.076,1.414)	0.079	0.617 (0.007,1.227)	0.047	0.852 (-0.274,1.978)	0.138
Knee LoS Dep 5	-0.035 (-0.087,0.018)	0.194	0.709 (-0.225,1.643)	0.137	0.015 (-0.042,0.072)	0.606	0.123 (-0.701,0.947)	0.770	-0.387 (-1.304,0.531)	0.409	1.104 (0.188,2.02)	0.018
Knee Bed Occ	0.993 (0.986,0.999)	0.032	0.834 (0.728,0.956)	0.009	1 (0.991,1.009)	0.991	1.42 (1.308,1.542)	0.000	1.373 (1.213,1.553)	0.000	1.465 (1.318,1.627)	0.000
Knee Public Private	0.006 (-0.02,0.031)	0.667	-0.476 (-1.026,0.074)	0.090	-0.015 (-0.04,0.009)	0.225	0.113 (-0.089,0.314)	0.274	0.276 (0.035,0.517)	0.025	0.131 (-0.071,0.332)	0.205
Elec Emerg Ratio	-0.005 (-0.013,0.003)	0.198	-0.322 (-0.455,-0.189)	0.000	-0.016 (-0.026,-0.005)	0.004	-0.008 (-0.107,0.091)	0.872	-0.053 (-0.18,0.074)	0.414	-0.028 (-0.163,0.108)	0.690

Jones T, *et al. BMJ Open* 2023; 13:e066398. doi: 10.1136/bmjopen-2022-066398
