# Supplementary material for: Impact of pausing elective hip and knee replacement surgery during winter 2017 on subsequent service provision at a major NHS Trust: a descriptive observational study using interrupted time series
Source: BMJ Open. 2023 May 16;13(5):e066398. doi: 10.1136/bmjopen-2022-066398 (PMC10193088; doi:10.1136/bmjopen-2022-066398)
Supplement: Author's manuscript [file bmjopen-2022-066398.draft_revisions.pdf]

BMJ Open

BMJ Open is committed to open peer review. As part of this commitment we make the peer review history of every article we publish publicly available.

When an article is published we post the peer reviewers' comments and the authors' responses online. We also post the versions of the paper that were used during peer review. These are the versions that the peer review comments apply to.

The versions of the paper that follow are the versions that were submitted during the peer review process. They are not the versions of record or the final published versions. They should not be cited or distributed as the published version of this manuscript.

BMJ Open is an open access journal and the full, final, typeset and author-corrected version of record of the manuscript is available on our site with no access controls, subscription charges or pay-per-view fees (http://bmjopen.bmj.com).

If you have any questions on BMJ Open's open peer review process please email info.bmjopen@bmj.com

BMJ Open

The impact of pausing elective hip and knee replacement surgery during winter 2017 on subsequent service provision at a major NHS Trust: a natural experiment

Journal:	*BMJ Open*
Manuscript ID	bmjopen-2022-066398
Article Type:	Original research
Date Submitted by the Author:	07-Jul-2022
Complete List of Authors:	Jones, Tim; University Hospitals Bristol and Weston NHS Foundation Trust, NIHR ARC West; University of Bristol, Department of Population Health Sciences<br>Penfold, Chris; University Hospitals Bristol and Weston NHS Foundation Trust, NIHR ARC West; University of Bristol, Musculoskeletal Research Unit<br>Redaniel, Maria Theresa; University Hospitals Bristol and Weston NHS Foundation Trust, NIHR ARC West; University of Bristol, Population Health Sciences, Bristol Medical School<br>Eyles, Emily; University Hospitals Bristol and Weston NHS Foundation Trust, NIHR ARC West; University of Bristol, Population Health Sciences, Bristol Medical School<br>Keen, Tim; North Bristol NHS Trust<br>Elliott, Andrew; North Bristol NHS Trust<br>Blom, AW; University of Bristol, Translational Health Sciences, Bristol Medical School<br>Judge, Andrew; University of Bristol, Musculoskeletal Research Unit
Keywords:	Knee < ORTHOPAEDIC & TRAUMA SURGERY, Hip < ORTHOPAEDIC & TRAUMA SURGERY, Orthopaedic & trauma surgery < SURGERY, Human resource management < HEALTH SERVICES ADMINISTRATION & MANAGEMENT

SCHOLARONE™
Manuscripts

I, the Submitting Author has the right to grant and does grant on behalf of all authors of the Work (as defined in the below author licence), an exclusive licence and/or a non-exclusive licence for contributions from authors who are: i) UK Crown employees; ii) where BMJ has agreed a CC-BY licence shall apply, and/or iii) in accordance with the terms applicable for US Federal Government officers or employees acting as part of their official duties; on a worldwide, perpetual, irrevocable, royalty-free basis to BMJ Publishing Group Ltd ("BMJ") its licensees and where the relevant Journal is co-owned by BMJ to the co-owners of the Journal, to publish the Work in this journal and any other BMJ products and to exploit all rights, as set out in our licence.

The Submitting Author accepts and understands that any supply made under these terms is made by BMJ to the Submitting Author unless you are acting as an employee on behalf of your employer or a postgraduate student of an affiliated institution which is paying any applicable article publishing charge ("APC") for Open Access articles. Where the Submitting Author wishes to make the Work available on an Open Access basis (and intends to pay the relevant APC), the terms of reuse of such Open Access shall be governed by a Creative Commons licence – details of these licences and which Creative Commons licence will apply to this Work are set out in our licence referred to above.

Other than as permitted in any relevant BMJ Author's Self Archiving Policies, I confirm this Work has not been accepted for publication elsewhere, is not being considered for publication elsewhere and does not duplicate material already published. I confirm all authors consent to publication of this Work and authorise the granting of this licence.

For peer review only

Title: The impact of pausing elective hip and knee replacement surgery during winter 2017 on subsequent service provision at a major NHS Trust: a natural experiment

Authors: Tim Jones[1,2], Christopher Penfold[1,2,3], Maria Theresa Redaniel[1,2], Emily Eyles[1,2], Tim Keen[4], Andrew Elliott[4], Ashley Blom[2,3], Andrew Judge[3,5]

[1] NIHR ARC West, University Hospitals Bristol NHS Foundation Trust, Bristol, BS1 2NT, UK

[2] Department of Population Health Sciences, Bristol Medical School, University of Bristol, BS8 2PS, UK

[3] Musculoskeletal Research Unit, Translational Health Sciences, Bristol Medical School, University of Bristol, Learning and Research Building, Level 1, Southmead Hospital, Bristol, BS10 5NB, UK

[4] North Bristol NHS Trust, Southmead Hospital, Westbury-on-Trym, Bristol, BS10 5NB, UK

[5] National Institute for Health Research Bristol Biomedical Research Centre, University Hospitals Bristol and Weston NHS Foundation Trust and University of Bristol, Bristol, UK

Corresponding author:

Tim Jones
Research Fellow
NIHR ARC West,
9th Floor Whitefriars,
Lewin's Mead
Bristol, BS1 2NT
United Kingdom

Tel: ++44 (0)117 342 1265
Timothy.Jones@bristol.ac.uk

Word count [3,560]

For peer review only

ABSTRACT

Objectives: To explore the impact of a temporary cancellation of elective surgery in winter 2017 on trends in primary hip and knee replacement at a major NHS Trust, and whether lessons can be learnt about efficient surgery provision.

Design and Setting: Observational interrupted time series analysis using hospital records to explore trends in primary hip and knee replacement surgery at a major NHS Trust, as well as patient characteristics, 2016-2019.

Intervention: A temporary cancellation of elective services for two months in winter 2017

Outcomes: NHS-funded hospital admissions for primary hip or knee replacement, length of stay and bed occupancy. Additionally, we explored the ratio of elective to emergency admissions at the Trust as a measure of elective capacity, and the ratio of public to private provision of NHS-funded hip and knee surgery.

Results: After winter 2017 there was a sustained reduction in the number of knee replacements, a decrease in the proportion of most deprived people having knee replacements, and an increase in average age for knee replacement and comorbidity for both types of surgery. The ratio of public to private provision dropped after winter 2017, and elective capacity generally has reduced over time. There was clear seasonality in provision of elective surgery, with less-complex patients admitted during winter.

Conclusions: Declining elective capacity and seasonality has a marked effect on the provision of joint replacement, despite efficiency improvements in hospital treatment. The Trust has outsourced less complex patients to independent providers, and/or treated them during winter when capacity is most limited. There is a need to explore whether these are strategies that could be used explicitly to maximise the use of limited elective capacity, provide benefit to patients, and value for money for taxpayers.

For peer review only - http://bmjopen.bmj.com/site/about/guidelines.xhtml

STRENGTH AND LIMITATIONS OF THIS STUDY

- Trends analyses using data obtained from the electronic health records of a local hospital NHS Trust are informative for clinicians and service managers in monitoring changes in planning and delivery of elective surgery, and could be regularly updated in near real time for monitoring.
- The inclusion of wider hospital admissions data beyond the NHS Trust allows us to estimate the proportion of people within the Trust catchment area having NHS-funded treatment at independent providers.
- We report the experience of one NHS Trust that is one of the larger elective orthopaedic centres - the findings may not be generalisable to or reflect the experience of other trusts.
- Our study does not include privately funded, privately provided hip and knee surgery which may also have been changing over time.

For peer review only

INTRODUCTION

Primary hip and knee replacement operations are common planned elective surgical procedures. They are highly clinically effective for improving symptoms of pain and functional limitations, and have been shown to be safe and cost-effective.[1-4] Around 100,000 hip[4] and over 100,000 knee operations[3] are carried out each year in the UK. Demand for these operations has been increasing substantially in recent decades[5] with an ageing population, rising levels of obesity, and widening indications for surgery in younger patient groups.[3 4]

Orthopaedic services have become more efficient over time, with length of hospital stay for primary hip and knee replacements reducing from around 15 days in 1997 to roughly 5.5 days in 2014.[6] This is largely due to the introduction of 'fast track' surgery and enhanced recovery services,[7] which reduce length of stay whilst maintaining patient safety and outcomes of surgery.[6] However, over the past decade there has also been a reduction in the numbers of hospital beds and operating theatres available for hip and knee replacement patients.[8] Waiting lists for orthopaedic procedures have been growing over time, and the average time people wait for treatment once on the waiting list has also increased.[9]

Pressures on elective surgery are exacerbated during winter, when resources for planned surgery are often displaced by more acute, unplanned hospital admissions.[8] At the end of 2017, this led to all planned elective hip and knee replacement operations in England being cancelled for the whole of January.[10] Even before the COVID-19 pandemic, over half a million people were already on the waiting list.[11] Patients are having to wait longer with deteriorating severe pain and functional limitation, affecting their health and quality of life. The COVID-19 pandemic has had an even greater impact on cancelling planned elective surgery, with over 635,000 people waiting for hip and knee replacements in April 2021, more than 10% of these waiting over a year, and over a third waiting longer than the 18 week target.[11]

For peer review only - http://bmjopen.bmj.com/site/about/guidelines.xhtml

The winter of 2017 provides a form of 'natural experiment', where elective capacity was intentionally reduced close to zero. A natural experimental design is a valid methodological approach to evaluate the impact of a range of events, policies and interventions which are not under the control of researchers.[12] Researchers can use the variation in exposure that natural experiments generate to analyse their impact on health outcomes. This provides a form of quasi-experimental study, where we can explore trends in provision of elective surgery before and after Winter 2017, which is a robust approach to explore real-world impact when randomisation is not possible.[13 14]

Our aim was to understand what happens after common, planned elective surgery is temporarily cancelled, and how this might inform optimum planning of elective surgery when capacity is limited, such as following the COVID-19 pandemic. We used interrupted time series analysis to model trends in elective hip and knee replacement surgery for a major NHS Trust from 2016 to 2019 and see how these were impacted by the withdrawal of elective surgery in winter 2017. We explored these trends by patient factors (age, sex, deprivation, number of comorbidities) and seasonality to see when demand was highest for different patient groups.

METHODS

This study is a longitudinal observational study using routinely collected administrative information about patients admitted to a major NHS Trust for elective hip and knee replacements, 2016 to 2019. It was developed and reported according to the RECORD extension[15] to STROBE guidelines for observational studies using routinely collected data.

Data Sources

We used two data sources for our analyses. The first was an extract of elective primary hip and knee replacement inpatient admissions identified from the Trust's electronic medical records (EMR) between 1st January 2016 and 31st December 2019. Up to 29 diagnoses were provided per entry using the International Classification of Diseases version 10 (ICD-10), and up to 11 procedures were provided

For peer review only - http://bmjopen.bmj.com/site/about/guidelines.xhtml

per entry using the Office of Population Censuses and Surveys Classification of Interventions and Procedures version 4 (OPCS-4). The extract included patient demographics such as age, sex, deprivation quintile, and comorbidities; and other characteristics of the hospital admissions such as length of stay. This data source was used for all analyses of hip and knee replacements at the Trust, including those relating to patient demographics, length of stay, and bed occupancy.

The second data source was pseudonymised national admitted patient care Hospital Episode Statistics (HES-APC) between 1st January 2016 and 31st December 2019. HES-APC is a routinely collected dataset that records all episodes of admitted (day case or inpatient) care provided to patients at NHS hospitals in England and to NHS-funded patients treated in independent hospitals.[16] Each episode represents a period of care under one consultant team. Up to 20 diagnoses and 24 clinical procedures are recorded per episode using ICD-10 codes and OPCS-4 codes, respectively. HES also includes the Lower Super Output Area (LSOA; an area of around 1,500 people) of residence for each patient, which can be linked to CCG of residence. This data source was used to estimate elective capacity overall at the Trust, and the ratio of public/private provision of hip and knee replacements in the catchment area for the Trust (see details below), which could not be gathered from the extract provided from the Trust EMR.

Hospital admissions for hip and knee replacements

Hospital admissions for elective hip and knee replacements were identified by entries with a primary procedure code representing primary hip or knee replacement (Supplementary Table T1) using the Trust EMR. We used this information to explore summary characteristics of the hospital admissions over time (overall counts of admissions, average age, proportion of women, proportion with 2+ comorbidities, proportion in the two most deprived quintiles) stratified by primary hip or knee replacements.

Length of stay and bed occupancy

For peer review only - http://bmjopen.bmj.com/site/about/guidelines.xhtml

149 We used the average number of overnight stays in hospital (days) for length of stay, trimmed at 30 days to exclude a small number of outliers (n=32, 0.6%). Trimming allowed us to model averages assuming a roughly normal distribution which we felt was more easily interpretable. Bed occupancy was the total number of beds used overnight for hip and knee replacement patients.

153 **Comorbidity of Admissions**

154 For each admission, we counted the number of conditions from the Charlson comorbidity index[17] recorded in the diagnosis fields. The Charlson index provides a summary of weighted scores relating to different comorbidities and has been shown to be associated with mortality. Admissions were categorised into those with zero, one, and two or more Charlson comorbidities.

158 **Ratio of Elective to Emergency Admissions**

159 To estimate the ratio of elective to emergency admissions for all purposes at the Trust (as a proxy for elective capacity), we extracted all hospital admissions from HES-APC with the Trust as a provider and categorised them into elective and emergency (admission method beginning with ‘1’ or ‘2’, respectively).

163 **Ratio of Public to Private Provision of Hip and Knee Replacements**

164 To estimate the ratio of public to private provision of NHS-funded elective hip and knee surgery for the Trust catchment area, we extracted all hospital admissions for primary hip and knee replacements (codes in Supplementary Table T1) for residents of the major local clinical commissioning groups (CCGs) from HES-APC (using 2021 CCG boundaries after local CCGs had merged into one CCG[18]), and categorised providers into public and private (provider code beginning with ‘R’ or ‘N’, respectively).

170 **Statistical Analysis**

171 For each of our outcomes, we conducted interrupted time series (ITS) analyses using segmented regression models comparing hospital admissions in the ‘before’ period (January 2016 to November

For peer review only - http://bmjopen.bmj.com/site/about/guidelines.xhtml

2017) to the 'after' period (February 2018 to December 2019). We excluded the winter 2017 period when admissions were very low (December 2017 and January 2018). The ITS analyses explored the trend before winter 2017, and how this trend changed after winter 2017[12 19], allowing for an immediate step change in February 2018 and a change in slope afterwards. We explored seasonality in the data by including indicator variables for spring, summer, and autumn[19] compared to winter as a baseline, and adjusted for serial autocorrelation using Newey-West standard errors with a maximum lag of two[20-22]. For count or proportion outcomes (number of admissions, proportion women, proportion with 2+ comorbidities, proportion in top two deprivation quintiles, bed occupancy) segmented Poisson regression models were fit to the data, whilst for averages/ratios (average age, average length of stay, ratio of elective to emergency admissions, ratio of public to private provision) segmented linear regression models were fit, using the 'glm' command in Stata. Sensitivity analyses were conducted adjusting the maximum lag for serial autocorrelation to zero and five; this would not affect point estimates but could alter standard errors, confidence intervals, and p-values.

All statistical analyses were conducted using Stata/MP version 16.1. Smoothed trends were fit to the data on all plots using the 'lowess' command with bandwidth 0.3. Stata code is available at: https://github.com/jonestim2002/hdr_uk_hospital_efficiency

Patient and public involvement

Initial research ideas for the grant application of which this work is part were presented to the public in a workshop and suggestions and comments were incorporated in the protocol. Feedback during the workshop was positive, with participants agreeing with the research objectives and the identified need.

RESULTS

Descriptive information and demographics

For peer review only - http://bmjopen.bmj.com/site/about/guidelines.xhtml

A total of 2,623 patients had a hip replacement and 2,674 had a knee replacement at the Trust in the 4 years between 2016 and 2019. The mean age of patients was 67 years and 60% were women for both types of operations.

Trends in hip and knee elective hospital admissions over time

The overall numbers of elective primary hip and knee replacement operations gradually declined over the study period from 63 hip and 65 knee replacements per month in 2016 to 49 hip and 51 knee replacements per month in 2019. Whilst there was a drop off in winter 2017, after elective surgery was re-started hip replacements resumed at similar numbers and continued to decline along a similar trajectory. Numbers of knee replacements dropped by 16% after winter 2017 (RR=0.843, 95% CI: 0.728 to 0.976, p=0.022), and the slope appeared to level off, although there was little evidence for this in the regression model (RR=1.005, 95% CI: 0.996 to 1.014, p=0.256); see Figure 1 and Supplementary Table T2. Hip and knee operations were clearly seasonal, with higher admissions in non-winter months compared to winter; 21% higher in the highest season (summer) for hips (RR=1.207, 95% CI: 1.094 to 1.332, $p<0.001$), and 31% higher in the highest season (spring) for knee replacements (RR=1.308, 95% CI: 1.157 to 1.479, $p<0.001$), excluding winter 2017.

Age on Admission

There was evidence of seasonality in average age on admission for hip admissions, where the mean age of patients was 66 in winter compared to 68 in summer (+2.09; 95% CI: 0.81 to 3.37, p = 0.001), suggesting that older patients might be more likely to receive surgery in the summer months. There was a change in the trend in average age for knee replacements after winter 2017 (+0.21, 95% CI: 0.12 to 0.31, $p < 0.001$) towards treating older patients (+1.59 years of age per year); see Figure 2.

Proportion Women

For peer review only

For peer review only - http://bmjopen.bmj.com/site/about/guidelines.xhtml

A higher proportion of hip replacements were performed on women in the summer (64%) compared to winter (58%) months (RR=1.088, 95% CI: 1.001 to 1.183, p=0.048). There were no clear patterns for knee replacements.

Comorbidity of Admissions

There was seasonality in the comorbidity of people having hip replacements, with a higher proportion of people having 2+ comorbidities in the summer (15.9%) compared to winter (12.3%) months (RR=1.306, 95% CI: 1.096 to 1.557, p=0.003). There was also a step change up in the proportion having hip replacements with 2+ comorbidities after winter 2017 (RR=1.411, 95% CI: 1.064 to 1.873, p=0.017), and an upward slope change for knee replacements (RR=1.042, 95% CI: 1.017 to 1.067, p=0.001); see Figure 3.

Deprivation

There was a higher proportion of more deprived people (quintiles 4 and 5) having knee replacements in the spring (37.6%) compared to the winter (30.2%) months (RR=1.224, 95% CI: 1.077 to 1.49, p=0.002). Additionally, there was evidence of a reducing proportion of the most deprived people having knee replacements after winter 2017 (RR=0.986, 95% CI: 0.974 to 0.998, p=0.021).

Ratio of elective admissions to emergency admissions at the Trust

There was an overall downward trend in the ratio of elective to emergency admissions at the Trust, from an average of 2.91 (SD: 0.17) electives for every emergency in 2016 to 2.16 (SD: 0.06) in 2019; see Supplementary Figure F1. There was no indication of seasonality, but the ratio reduced after winter 2017 (RR=0.725, 95% CI: 0.64 to 0.821, p<0.001), and started to decrease more rapidly afterwards (RR=984, 95% CI: 0.974 to 0.995, p=0.003).

For peer review only - http://bmjopen.bmj.com/site/about/guidelines.xhtml

Ratio of public to private provision of hip/knee elective surgery at the Trust

The ratio of public to private provision was higher in the summer (1.56 for hips and 1.28 for knees) compared to winter (1.22 and 0.99, respectively) months (hips RR=1.361, 95% CI 1.166 to 1.589, p<0.001; knees RR=1.318, 95% CI: 1.035 to 1.678, p=0.025). There was also evidence of a step change downwards in public provision compared to private provision after winter 2017 for both types of surgery, but particularly for hip replacements (hips RR=0.477, 95% CI: 0.29 to 0.782, p=0.003; knees RR=0.621, 95% CI: 0.358 to 1.077, p=0.09); see Figure 4.

Bed Occupancy

Bed occupancy for hip and knee replacements at the Trust was very seasonal, with lower occupancy in the winter months compared to all other seasons; e.g. summer bed occupancy was 324 beds for hips and 291 beds for knees on average compared to winter bed occupancy of 225 beds for hips and 199 beds for knees on average. In both cases bed occupancy has reduced over time, although there wasn't evidence of this in the regression model for hip replacements, and there was a step change downwards (RR=0.834, 95% CI: 0.704 to 0.989, p=0.037) for knee surgery after winter 2017; see Figure 5.

Length of Stay

The average length of hospital stay was 5.5 days (SD: 5.9 days) for hip replacements and 5.2 days (SD: 5.0 days) for knee replacements in 2016, compared to 5.1 days (SD: 4.1 days) and 4.3 days (SD: 3.4 days) respectively in 2019 (see Supplementary Figure F2). Length of stay was longer in spring than winter for hip replacements (0.502 days, 95% CI: 0.214 to 0.79, p=0.001), and longer in spring

For peer review only - http://bmjopen.bmj.com/site/about/guidelines.xhtml

(0.422, 95% CI: 0.073 to 0.771, p=0.018) and autumn (0.396, 95% CI: 0.015 to 0.777, p=0.042) compared to winter for knee replacements.

DISCUSSION

Principle findings

The temporary cancellation of elective services during winter 2017 does appear to have had some impact on service provision at the Trust after that time. There was an immediate and sustained reduction in the number of knee replacements being done at the Trust and this was also reflected in the drop in bed occupancy for knee surgery. The average age for knee replacement and comorbidity of hip and knee surgery patients increased after winter 2017, whilst the proportion of more deprived people having knee replacements decreased, and the ratio of public to private provision of hip and knee replacements in the local area dropped after winter 2017. This suggests an NHS-funded outsourcing of less comorbid hip and knee replacement surgery to independent providers, and therefore on average the patients being treated at the Trust became older and more comorbid. There was a general decrease in capacity for elective surgery at the Trust (ratio of elective to emergency admissions), mostly driven by increasing non-elective admissions even before the COVID-19 pandemic. The winter 2017 cancellation may have been just one symptom of this overall pressure on elective surgery that underlies some of the longer-term changes in provision.

There was also some seasonality in service provision. It is no surprise that elective admissions and bed occupancy are lower in winter when the hospital requires capacity for an increase in unplanned admissions. There were also indications that people being admitted in winter were younger, less comorbid, and less deprived (particularly for knee surgery). Length of stay for hip and knee replacements was lower in winter compared to spring. This suggests the admission of younger, less

For peer review only

comorbid patients during the winter months given the reduced elective capacity and delaying surgery for more comorbid patients to when capacity is higher in the following months.

Strengths and limitations

Trends analyses such as these, using data obtained from the EHR of a local hospital NHS Trust, are informative for clinicians and service managers in monitoring changes in planning and delivery of elective surgery, and could be regularly updated in near real time for monitoring. This concept might be informative for other commissioning groups / Trusts to adopt for monitoring of their own elective surgery and capacity. We report the experience of just one trust that is one of the larger elective orthopaedic centres, and hence the findings may not be generalisable to or reflect the experience of other trusts. We should be aware that some results may reflect chance findings due to multiple testing and type 1 error. The trends in the data as plotted do not change substantially in sensitivity analyses accounting for different autocorrelation lags (Supplementary Tables T3-T4). The catchment area of the Trust is not exactly the same as the major local CCG and is difficult to define exactly. However, 89.4% of admissions at the Trust were for residents of the local CCG and we felt this was a reasonable approximation to estimate the ratio of public to private provision in the Trust catchment area. Our analyses only include NHS-funded surgery and not privately-funded, privately-provided surgery.

Comparison to other studies

A previous study[23] using data for England from Hospital Episode Statistics found increasing private provision of elective hip arthroplasties nationally from 2007/8 to 2012/13, particularly for less deprived people, which echoes our findings. More recent news stories have suggested that 20% of NHS-funded hip replacements and 29% of NHS-funded knee replacements were carried out by independent providers in 2016/17[24], and that independently-provided hip and knee replacement surgery (privately or NHS-funded) has now overtaken NHS provision.[25] A UK-wide study[6] using

For peer review only - http://bmjopen.bmj.com/site/about/guidelines.xhtml

primary care data (CPRD) linked to hospital admissions found similar effects of patient characteristics (age, sex, comorbidity, and deprivation) on length of stay for primary hip and knee replacements, although they did not explore seasonality. A recent qualitative study[26] highlighted the negative financial and emotional impact of winter elective cancellations on patients and their families and recommended better advanced planning of elective operations to reduce these impacts.

Implications for clinicians and policy makers

Outsourcing of less complex hip and knee replacements to take advantage of spare capacity in non-NHS hospitals may be a good strategy to reduce waiting times and waiting lists for surgery and get the best results for patients given the evident capacity limitations. However, this would leave the NHS Trust to cope with more complex cases and has training implications because trainee surgeons are usually trained by first undertaking less-complex cases on healthier patients. There are also potential equity implications, if less complex cases have the option of surgery with shorter waiting times at independent providers, whilst more complex (and potentially more deprived) cases do not. We would need to consider the acceptability of this outsourcing to patients and practitioners.

There is an indication that some selection of patients for elective surgery depending on available capacity already takes place at the Trust. It is possible that this could become a more explicit strategy, based on evidence, to optimise the use of limited capacity in hospitals at different times of the year. However, this could mean that people placed earlier on the waiting list for surgery might get their surgery later due to such scheduling strategies, so acceptability to patients would need to be explored. We need to understand how the scheduling and possible outsourcing of elective surgery for different types of patients, depending on capacity, may impact on throughput of patients, waiting times, waiting lists, outcomes of surgery, costs, and equity of access to surgery. Inevitably outsourcing simpler patients to the independent sector will leave more complex patients being treated by NHS Trusts, which could have a detrimental impact on their service. An appropriate

For peer review only - http://bmjopen.bmj.com/site/about/guidelines.xhtml

For peer review only

balance would need to be achieved to maximise the benefits for patients, and research is needed to understand what that balance is. Additionally, we need to understand whether this type of scheduling and outsourcing is acceptable to people waiting for hip and knee surgery as well as clinicians. These issues of optimising limited elective resources are in even sharper focus due to the backlog in waiting lists caused by the COVID-19 pandemic.

Unanswered questions and future research

We need to understand how the scheduling and possible outsourcing of elective surgery for different types of patients, depending on capacity, may impact on throughput of patients, waiting times, waiting lists, outcomes of surgery, costs, and equity of access to surgery. Inevitably outsourcing simpler patients to the independent sector will leave more complex patients being treated by NHS Trusts, which could have a detrimental impact on their service. An appropriate balance would need to be achieved to maximise the benefits for patients, and research is needed to understand what that balance is. Additionally, we need to understand whether this type of scheduling and outsourcing is acceptable to people waiting for hip and knee surgery as well as clinicians.

Conclusions

Declining elective capacity and seasonality has a marked effect on the provision of joint replacement, despite efficiency improvements in hospital treatment. The Trust has outsourced less complex patients to independent providers, and/or treated them during winter when capacity is most limited. There is a need to explore whether these are strategies that could be used explicitly to maximise the use of limited elective capacity, provide benefit to patients, and value for money for taxpayers.

Author Contributions

For peer review only - http://bmjopen.bmj.com/site/about/guidelines.xhtml

This publication is the work of the authors, who serve as guarantors for the contents of this paper. TJ contributed to study design, data cleaning, data analysis, interpretation of results and writing the manuscript. MTR contributed to study conceptualisation, supervision, interpretation of results and reviewing the manuscript. TK contributed to data curation, supervision, interpretation of results and reviewing the manuscript. AE contributed to data curation, interpretation of results and reviewing the manuscript. CP and EE contributed to interpretation of results and reviewing the manuscript. AB contributed to study conceptualisation, supervision, interpretation of results and reviewing the manuscript. AJ contributed to study conceptualisation and design, supervision, and writing the manuscript. TJ had full access to the data in the study and takes responsibility for the integrity of the data and the accuracy of the data analysis.

Acknowledgements

The study reported here would not have been possible without information provided by the NHS Trust, and the use of their hospital admissions data via the NIHR ARC West Partnership Agreement.

Funding

This research was funded by Health Data Research UK (HDR UK) Better Care South-West Partnership. EE, TJ, and MTR's time was supported by the National Institute for Health Research Applied Research Collaboration West (NIHR ARC West). The views expressed in this article are those of the author(s) and not necessarily those of the NHS, the NIHR, the Department of Health and Social Care or HDR UK.

Ethical Approval

For peer review only - http://bmjopen.bmj.com/site/about/guidelines.xhtml

We were provided with pseudonymised hospital admissions data from the NHS Trust under the NIHR ARC West Partnership Agreement. The project received ethical approval from the University of Bristol Faculty of Health Sciences ethical review board on 3rd November 2020 (ref# 109024).

We were provided with routinely-collected Hospital Episode Statistics data under licence from NHS Digital (DARS-NIC-17875-X7K1V). The licence allows us to use the information under Section 261 of the Health and Social Care Act 2012, 2(b)(ii): "after taking into account the public interest as well as the interests of the relevant person, considers that it is appropriate for the information to be disseminated".

Data Sharing

The data from the NHS Trust was obtained under the NIHR ARC West Partnership Agreement. The agreement precludes us from sharing the raw data but it can be published and shared once aggregated to a non-identifiable level.

The data from Hospital Episode Statistics (HES) was obtained under licence (DARS-NIC-17875-X7K1V) from NHS Digital (previously the Health and Social Care Information Centre); *Copyright © 2022, re-used with the permission of The Health & Social Care Information Centre. All rights reserved*. The data are provided by patients and collected by the NHS as part of their care and support. HES data can be accessed via NHS Digital: https://digital.nhs.uk/services/data-access-request-service-dars

Transparency

The manuscript's guarantor (TJ) affirms that the manuscript is an honest, accurate, and transparent account of the study being reported; that no important aspects of the study have been omitted; and that any discrepancies from the study as originally planned have been explained.

For peer review only - http://bmjopen.bmj.com/site/about/guidelines.xhtml

Competing Interests

All authors have completed the ICMJE uniform disclosure form at www.icmje.org/coi_disclosure.pdf and declare: TJ, EE, and TR had financial support from NIHR ARC West for the submitted work; AJ has had financial support in the previous three years through institutional grants from NIHR, HDR UK, Versus Arthritis, Healthcare Quality Improvement Partnership (HQIP), Royal College of Physicians (RCP), and Health Foundation, had unpaid committee or leadership roles relating to musculoskeletal conditions for NIHR, Nuffield Foundation, Warwick CTU, and Versus Arthritis, and a paid expert panel role for Nuffield Foundation Oliver Bird Fund; no other financial relationships with any organisations that might have an interest in the submitted work in the previous three years; no other relationships or activities that could appear to have influenced the submitted work.

For peer review only

REFERENCES

1. Carr AJ, Robertsson O, Graves S, et al. Knee replacement. *Lancet* 2012;379(9823):1331-40. doi: 10.1016/S0140-6736(11)60752-6
2. Pivec R, Johnson AJ, Mears SC, et al. Hip arthroplasty. *Lancet* 2012;380(9855):1768-77. doi: 10.1016/S0140-6736(12)60607-2
3. Price AJ, Alvand A, Troelsen A, et al. Hip and knee replacement 2 Knee replacement. *Lancet* 2018;392(10158):1672-82. doi: Doi 10.1016/S0140-6736(18)32344-4
4. Ferguson RJ, Palmer AJ, Taylor A, et al. Hip replacement. *Lancet* 2018;392(10158):1662-71. doi: 10.1016/S0140-6736(18)31777-X [published Online First: 2018/11/30]
5. Culliford DJ, Maskell J, Beard DJ, et al. Temporal trends in hip and knee replacement in the United Kingdom 1991 TO 2006. *J Bone Joint Surg Br* 2010;92b(1):130-35. doi: 10.1302/0301-620x.92b1.22654
6. Burn E, Edwards CJ, Murray DW, et al. Trends and determinants of length of stay and hospital reimbursement following knee and hip replacement: evidence from linked primary care and NHS hospital records from 1997 to 2014. *Bmj Open* 2018;8(1) doi: 10.1136/bmjopen-2017-019146
7. Kehlet H. Fast-track hip and knee arthroplasty. *Lancet* 2013;381(9878):1600-02. doi: Doi 10.1016/S0140-6736(13)61003-X
8. Briggs T. A national review of adult elective orthopaedic services in England 2015. Available from: https://gettingitrightfirsttime.co.uk/wp-content/uploads/2017/06/GIRFT-National-Report-Mar15-Web.pdf.
9. Ward D, Wenzel L. A new trend in elective hip surgery 2019. Available from: https://www.kingsfund.org.uk/blog/2019/06/new-trend-elective-hip-surgery.
10. Fisher E, Merry L. Winter 2017/18: the worst ever for the NHS? 2018. Available from: https://www.nuffieldtrust.org.uk/news-item/winter-2017-18-the-worst-ever-for-the-nhs.

For peer review only - http://bmjopen.bmj.com/site/about/guidelines.xhtml

11. BMJ. Covid-19: "Life on hold" for NHS patients needing musculoskeletal care 2021. Available from: https://doi.org/10.1136/bmj.n1616.

12. Bernal JL, Cummins S, Gasparrini A. Interrupted time series regression for the evaluation of public health interventions: a tutorial. *Int J Epidemiol* 2017;46(1):348-55. doi: 10.1093/ije/dyw098

13. Kontopantelis E, Doran T, Springate DA, et al. Regression based quasi-experimental approach when randomisation is not an option: interrupted time series analysis. *Bmj-Brit Med J* 2015;350 doi: 10.1136/bmj.h2750

14. Leatherdale ST. Natural experiment methodology for research: a review of how different methods can support real-world research. *Int J Soc Res Method* 2019;22(1):19-35. doi: 10.1080/13645579.2018.1488449

15. Benchimol EI, Smeeth L, Guttmann A, et al. The REporting of studies Conducted using Observational Routinely-collected health Data (RECORD) Statement. *Plos Med* 2015;12(10) doi: 10.1371/journal.pmed.1001885

16. Herbert A, Wijlaars L, Zylbersztejn A, et al. Data Resource Profile: Hospital Episode Statistics Admitted Patient Care (HES APC). *Int J Epidemiol* 2017;46(4):1093-+. doi: 10.1093/ije/dyx015

17. Quan H, Sundararajan V, Halfon P, et al. Coding algorithms for defining comorbidities in ICD-9-CM and ICD-10 administrative data. *Med Care* 2005;43(11):1130-9. doi: 10.1097/01.mlr.0000182534.19832.83 [published Online First: 2005/10/15]

18. Office for National Statistics. LSOA (2011) to Clinical Commissioning Groups to Sustainability and Transformation Partnerships (April 2021) Lookup in England 2021. Available from: https://geoportal.statistics.gov.uk/datasets/ons::lsoa-2011-to-clinical-commissioning-groups-to-sustainability-and-transformation-partnerships-april-2021-lookup-in-england/explore.

19. Bhaskaran K, Gasparrini A, Hajat S, et al. Time series regression studies in environmental epidemiology. *Int J Epidemiol* 2013;42(4):1187-95. doi: 10.1093/ije/dyt092

20. Bottomley C, Scott JAG, Isham V. Analysing Interrupted Time Series with a Control. *Epidemiologic Methods* 2019;8(1) doi: 10.1515/em-2018-0010

21. Linden A. Combining synthetic controls and interrupted time series analysis to improve causal inference in program evaluation. *J Eval Clin Pract* 2018;24(2):447-53. doi: 10.1111/jep.12882

22. Dorward J, Khubone T, Gate K, et al. The impact of the COVID-19 lockdown on HIV care in 65 South African primary care clinics: an interrupted time series analysis. *Lancet Hiv* 2021;8(3):e158-e65. doi: 10.1016/S2352-3018(20)30359-3

23. Sutaria S, Kirkwood G, Pollock AM. An ecological study of NHS funded elective hip arthroplasties in England from 2003/04 to 2012/13. *J Roy Soc Med* 2019;112(7):292-303. doi: 10.1177/0141076819851701

24. The Independent. NHS privatisation fears after figures show third of knee replacements done by private sector 2018. Available from: https://www.independent.co.uk/news/uk/home-news/nhs-knee-hip-replacements-private-sector-a8549006.html.

25. Dench J. Private hospital overtakes NHS for hip and knee replacement 2021. Available from: https://londonnewstime.com/private-hospital-overtakes-nhs-for-hip-and-knee-replacement/479438/.

26. Herrod PJJ, Adiamah A, Boyd-Carson H, et al. Winter cancellations of elective surgical procedures in the UK: a questionnaire survey of patients on the economic and psychological impact. *Bmj Open* 2019;9(9) doi: 10.1136/bmjopen-2018-028753

For peer review only - http://bmjopen.bmj.com/site/about/guidelines.xhtml

FIGURE CAPTIONS

Figure 1. Elective hip (left panel) and knee (right panel) replacement admissions at the Trust
Note: grey area shows the winter 2017 cancellations and is excluded from the analysis

Figure 2. Average age on admission for hip (left panel) and knee (right panel) replacements at the Trust
Note: grey area shows the winter 2017 cancellations and is excluded from the analysis

Figure 3. Proportion of people having hip (left panel) and knee (right panel) replacements with 2+ Charlson comorbidities recorded
Note: grey area shows the winter 2017 cancellations and is excluded from the analysis

Figure 4. Ratio of public to private provision of elective hip (left panel) and knee (right panel) replacements for NHS patients in the Trust CCG
Note: grey area shows the winter 2017 cancellations and is excluded from the analysis

Figure 5. Bed occupancy for hip (left panel) and knee (right panel) replacements at the Trust
Note: grey area shows the winter 2017 cancellations and is excluded from the analysis

For peer review only

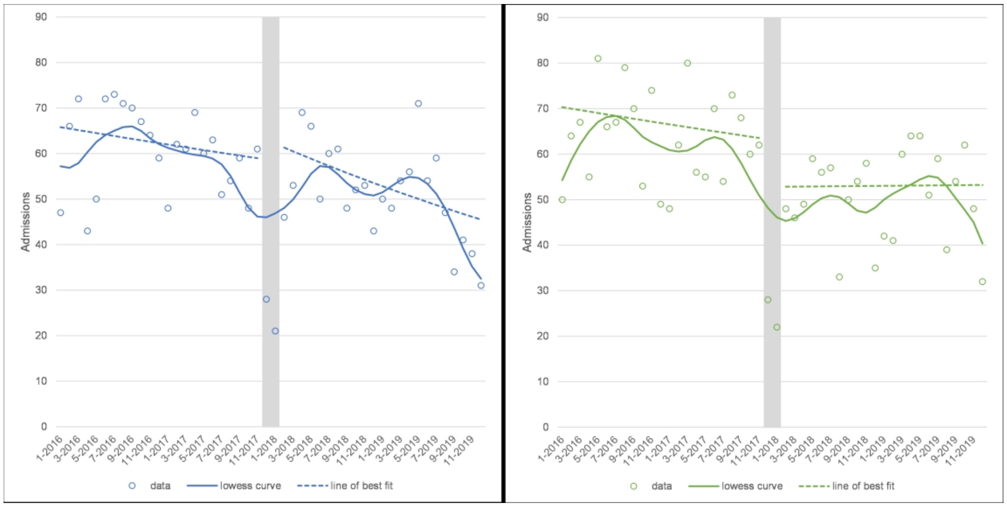

Figure 1. Elective hip (left panel) and knee (right panel) replacement admissions at the Trust
Note: grey area shows the winter 2017 cancellations and is excluded from the analysis

301x150mm (300 x 300 DPI)

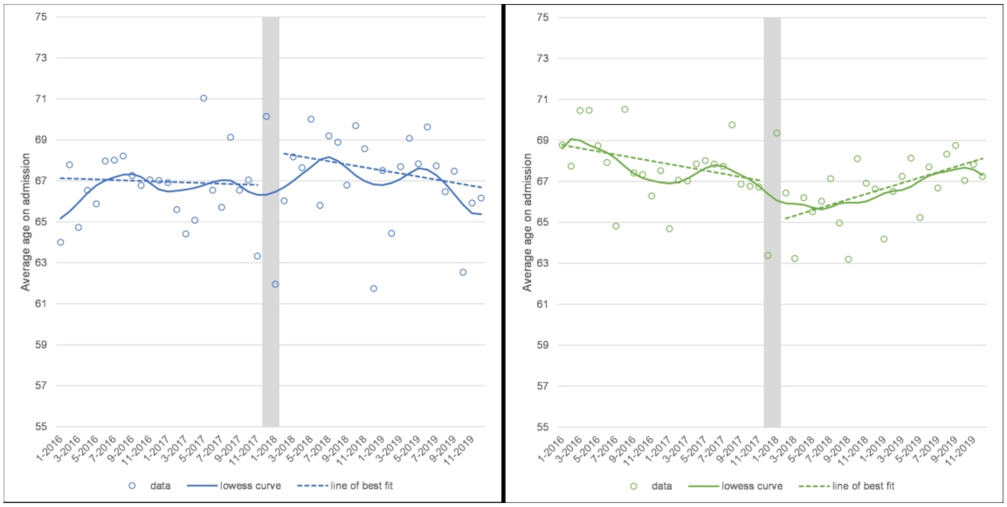

Figure 2. Average age on admission for hip (left panel) and knee (right panel) replacements at the Trust
Note: grey area shows the winter 2017 cancellations and is excluded from the analysis

301x150mm (300 x 300 DPI)

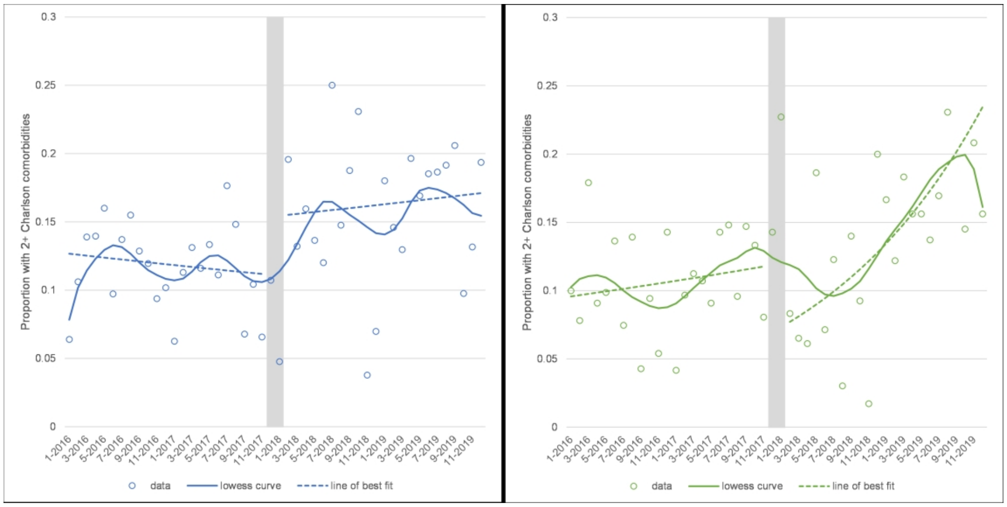

Figure 3. Proportion of people having hip (left panel) and knee (right panel) replacements with 2+ Charlson comorbidities recorded
Note: grey area shows the winter 2017 cancellations and is excluded from the analysis

301x150mm (300 x 300 DPI)

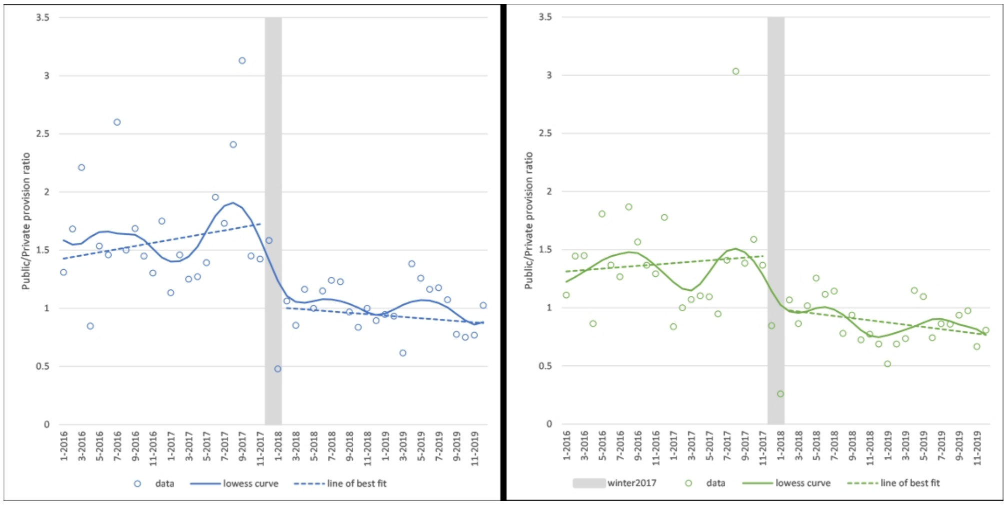

Figure 4. Ratio of public to private provision of elective hip (left panel) and knee (right panel) replacements for NHS patients in the Trust CCG
Note: grey area shows the winter 2017 cancellations and is excluded from the analysis

301x150mm (300 x 300 DPI)

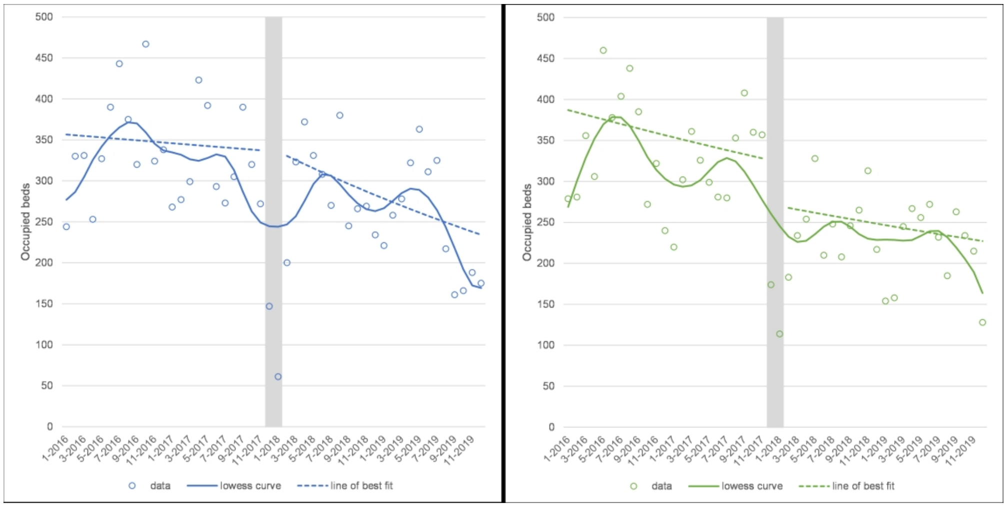

Figure 5. Bed occupancy for hip (left panel) and knee (right panel) replacements at the Trust
Note: grey area shows the winter 2017 cancellations and is excluded from the analysis

301x150mm (300 x 300 DPI)

Supplementary Figure F1. Ratio of elective to emergency hospital admissions for any reason at the Trust

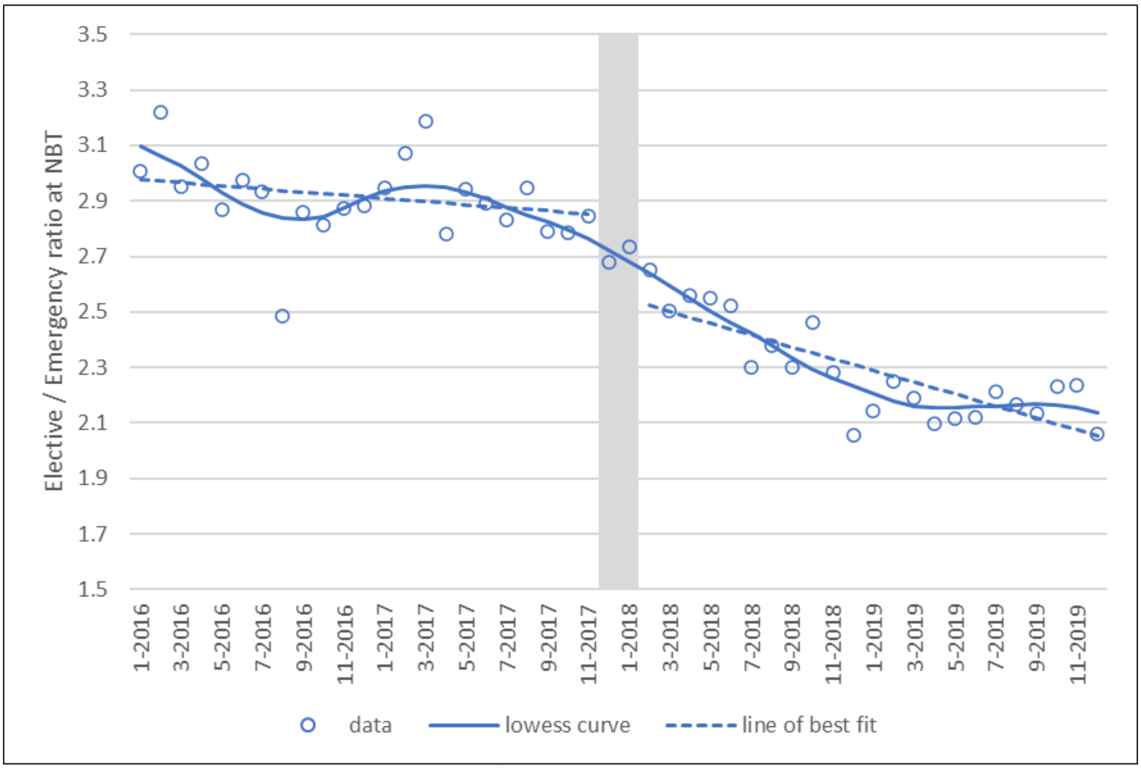

Note: grey area shows the winter 2017 cancellations and is excluded from the analysis

For peer review only

Supplementary Figure F2. Average length of stay for primary hip (left) and knee (right) replacement operations at NBT

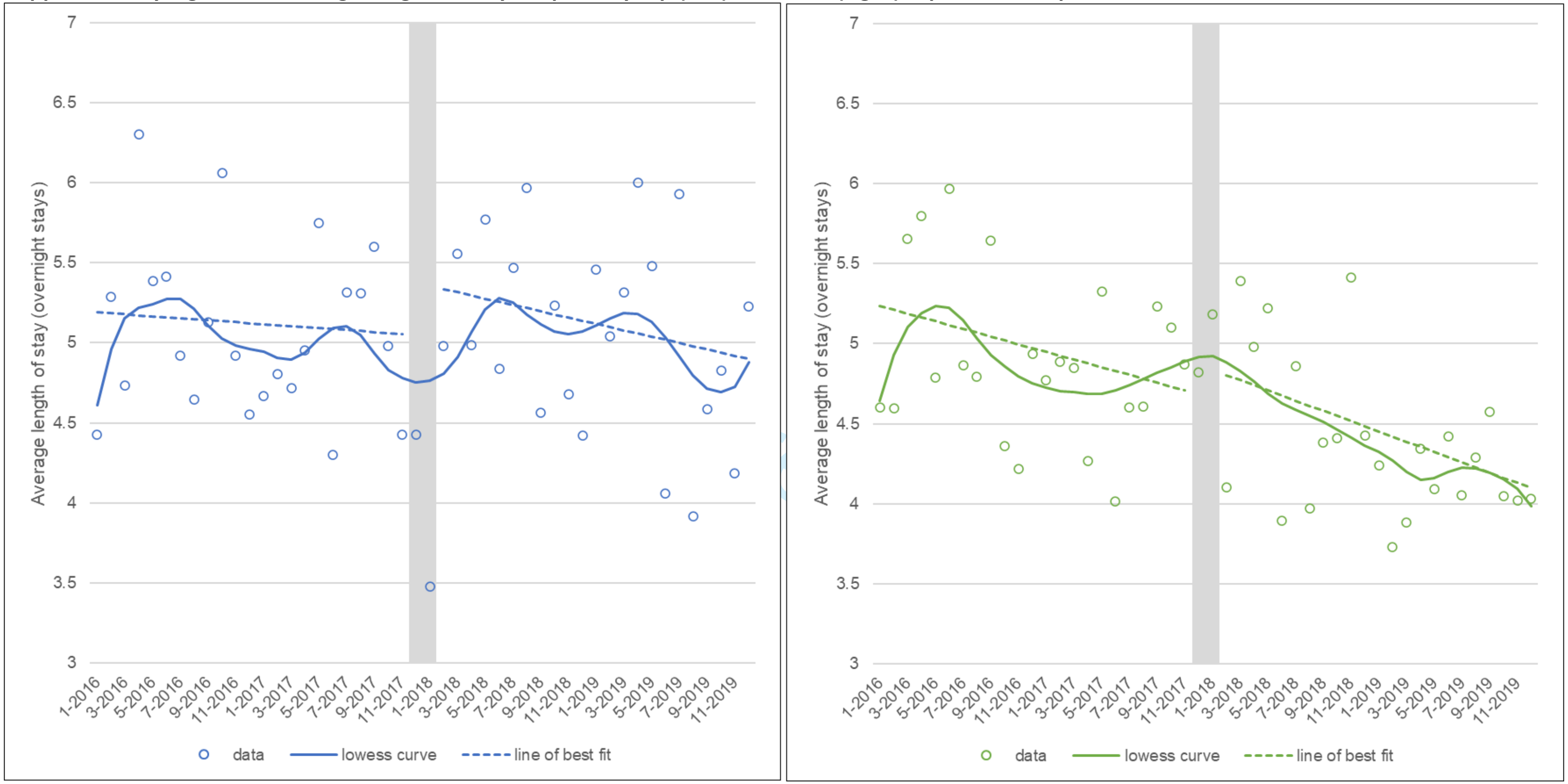

Note: grey area shows the winter 2017 cancellations and is excluded from the analysis

For peer review only

For peer review only - http://bmjopen.bmj.com/site/about/guidelines.xhtml

Supplementary Table T1. OPCS-4 codes used to identify primary hip and knee replacement operations

Category	Code	Description	Notes
Primary Total Hip Replacement	W37.1	Primary total prosthetic replacement of hip joint using cement	
	W37.8	Other specified total prosthetic replacement of hip joint using cement	
	W37.9	Unspecified total prosthetic replacement of hip joint using cement	
	W38.1	Primary total prosthetic replacement of hip joint not using cement	
	W38.8	Other specified total prosthetic replacement of hip joint not using cement	
	W38.9	Unspecified total prosthetic replacement of hip joint not using cement	
	W39.1	Primary total prosthetic replacement of hip joint NEC	
	W39.8	Other specified other total prosthetic replacement of hip joint	
	W39.9	Unspecified other total prosthetic replacement of hip joint	
	W43.1	Primary total prosthetic replacement of other joint using cement NEC	
	W43.8	Other specified total prosthetic replacement of other joint using cement NEC	
	W43.9	Unspecified total prosthetic replacement of other joint using cement NEC	
	W44.1	Primary total prosthetic replacement of other joint not using cement NEC	
	W44.8	Other specified total prosthetic replacement of other joint not using cement NEC	
	W44.9	Unspecified total prosthetic replacement of other joint not using cement NEC	
	W45.1	Other primary total prosthetic replacement of other joint NEC	
	W45.8	Other specified total prosthetic replacement of other joint NEC	
	W45.9	Unspecified total prosthetic replacement of other joint NEC	
	W52.1	Primary prosthetic replacement of articulation of bone using cement NEC	

For peer review only

For peer review only - http://bmjopen.bmj.com/site/about/guidelines.xhtml

W52.8	Other specified prosthetic replacement of articulation of bone using cement NEC
W52.9	Unspecified prosthetic replacement of articulation of bone using cement NEC
W53.1	Primary prosthetic replacement of articulation of bone not using cement NEC
W53.8	Other specified prosthetic replacement of articulation of bone not using cement NEC
W53.9	Unspecified prosthetic replacement of articulation of bone not using cement NEC
W54.1	Primary prosthetic replacement of articulation of bone NEC
W54.8	Other specified prosthetic replacement of articulation of bone NEC
W54.9	Unspecified prosthetic replacement of articulation of bone NEC
W93.1	Primary hybrid prosthetic replacement of hip joint using cemented acetabular component
W93.8	Other specified hybrid prosthetic replacement of hip joint using cemented acetabular component
W93.9	Unspecified hybrid prosthetic replacement of hip joint using cemented acetabular component
W94.1	Primary hybrid prosthetic replacement of hip joint using cemented femoral component
W94.8	Other specified hybrid prosthetic replacement of hip joint using cemented femoral component
W94.9	Unspecified hybrid prosthetic replacement of hip joint using cemented femoral component
W95.1	Primary hybrid prosthetic replacement of hip joint using cement NEC
W95.8	Other specified hybrid prosthetic replacement of hip joint using cement
W95.9	Unspecified hybrid prosthetic replacement of hip joint using cement

For peer review only - http://bmjopen.bmj.com/site/about/guidelines.xhtml

Primary Total Knee Replacement	W40.1	Primary total prosthetic replacement of knee joint using cement	
	W40.8	Other specified total prosthetic replacement of knee joint using cement	
	W40.9	Unspecified total prosthetic replacement of knee joint using cement	
	W41.1	Primary total prosthetic replacement of knee joint not using cement	
	W41.8	Other specified total prosthetic replacement of knee joint not using cement	
	W41.9	Unspecified total prosthetic replacement of knee joint not using cement	
	W42.1	Primary total prosthetic replacement of knee joint NEC	
	W42.8	Other specified other total prosthetic replacement of knee joint	
	W42.9	Unspecified other total prosthetic replacement of knee joint	
	O18.1	Primary hybrid prosthetic replacement of knee joint using cement	
	O18.8	Other specified hybrid prosthetic replacement of knee joint using cement	
	O18.9	Unspecified hybrid prosthetic replacement of knee joint using cement	
Resurfacing / Reconstruction	W58.1	Primary resurfacing arthroplasty of joint	Require combination with site + combination codes to ID
	W58.8	Other specified reconstruction of joint	Require combination with site + combination codes to ID
	W58.9	Unspecified other reconstruction of joint	Require combination with site + combination codes to ID
Primary unicondylar / unicompartmental knee operations	W52.1	Primary prosthetic replacement of articulation of bone using cement NEC	Require combination with site + combination codes to ID
	W52.8	Other specified prosthetic replacement of articulation of other bone using cement	Require combination with site + combination codes to ID
	W52.9	Unspecified prosthetic replacement of articulation of other bone using cement	Require combination with site + combination codes to ID
	W53.1	Primary prosthetic replacement of articulation of bone not using cement NEC	Require combination with site + combination codes to ID

For peer review only - http://bmjopen.bmj.com/site/about/guidelines.xhtml

W53.9	Unspecified prosthetic replacement of articulation of other bone not using cement	Require combination with site + combination codes to ID
W54.0	Conversion from previous prosthetic replacement of articulation of bone NEC	Require combination with site + combination codes to ID
W54.1	Primary prosthetic replacement of articulation of bone NEC	Require combination with site + combination codes to ID
W54.8	Other specified other prosthetic replacement of articulation of other bone	Require combination with site + combination codes to ID
W54.9	Unspecified other prosthetic replacement of articulation of other bone	Require combination with site + combination codes to ID

For peer review only

Supplementary Table T2. Interrupted time series model results with maximum auto-correlation lag 2

	pre-trend		level change		trend change		spring		summer		autumn	
	estimate (95% CI)	p	estimate (95% CI)	p	estimate (95% CI)	p	estimate (95% CI)	p	estimate (95% CI)	p	estimate (95% CI)	p
Hip Admissions	0.995 (0.986,1.004)	0.300	1.055 (0.912,1.221)	0.469	0.991 (0.977,1.006)	0.239	1.19 (1.034,1.37)	0.015	1.207 (1.094,1.332)	0.000	1.101 (0.982,1.234)	0.099
Hip Average Age	-0.015 (-0.102,0.072)	0.737	1.571 (-0.1,3.242)	0.065	-0.06 (-0.175,0.055)	0.307	1.52 (-0.07,3.111)	0.061	2.09 (0.811,3.368)	0.001	1.031 (-0.598,2.66)	0.215
Hip Prop Women	0.999 (0.995,1.003)	0.582	0.966 (0.861,1.083)	0.549	1.007 (0.999,1.016)	0.089	1.023 (0.941,1.111)	0.596	1.088 (1.001,1.183)	0.048	0.981 (0.888,1.083)	0.700
Hip Prop 2+ Charlson	0.994 (0.982,1.007)	0.380	1.411 (1.064,1.873)	0.017	1.01 (0.994,1.027)	0.220	1.195 (0.982,1.455)	0.075	1.306 (1.096,1.557)	0.003	1.003 (0.788,1.276)	0.982
Hip Prop High Deprivation	1.003 (0.993,1.013)	0.587	1.027 (0.869,1.214)	0.754	1.004 (0.986,1.022)	0.660	0.937 (0.815,1.076)	0.358	0.877 (0.752,1.022)	0.093	0.996 (0.84,1.18)	0.963
Hip LoS	-0.006 (-0.034,0.021)	0.660	0.312 (-0.193,0.818)	0.225	-0.014 (-0.047,0.02)	0.425	0.502 (0.214,0.79)	0.001	0.135 (-0.194,0.463)	0.422	0.1 (-0.288,0.488)	0.613
Hip Bed Occ	0.997 (0.987,1.008)	0.643	1 (0.844,1.186)	0.997	0.987 (0.97,1.005)	0.149	1.291 (1.11,1.501)	0.001	1.283 (1.125,1.463)	0.000	1.146 (0.988,1.328)	0.071
Hip Public Private	1.013 (0.985,1.041)	0.377	0.477 (0.29,0.782)	0.003	0.981 (0.951,1.011)	0.218	0.992 (0.809,1.217)	0.939	1.361 (1.166,1.589)	0.000	1.038 (0.847,1.273)	0.718
Knee Admissions	0.995 (0.99,1.001)	0.106	0.843 (0.728,0.976)	0.022	1.005 (0.996,1.014)	0.256	1.308 (1.157,1.479)	0.000	1.26 (1.138,1.396)	0.000	1.286 (1.164,1.42)	0.000
Knee Average Age	-0.078 (-0.157,0.001)	0.054	-1.632 (-2.988,-0.276)	0.018	0.211 (0.117,0.305)	0.000	0.926 (-0.112,1.965)	0.080	0.953 (-0.021,1.927)	0.055	0.354 (-0.531,1.24)	0.433
Knee Prop Women	1.004 (0.998,1.01)	0.150	0.96 (0.85,1.084)	0.513	0.994 (0.986,1.003)	0.193	1.037 (0.963,1.117)	0.336	1.017 (0.924,1.118)	0.735	1.036 (0.958,1.12)	0.375
Knee Prop 2+ Charlson	1.009 (0.993,1.026)	0.249	0.638 (0.455,0.894)	0.009	1.042 (1.017,1.067)	0.001	1.156 (0.911,1.467)	0.234	1.074 (0.849,1.359)	0.551	0.909 (0.628,1.315)	0.612
Knee Prop High Deprivation	1.005 (0.997,1.013)	0.189	0.968 (0.786,1.191)	0.758	0.986 (0.974,0.998)	0.021	1.224 (1.077,1.39)	0.002	1.075 (0.942,1.227)	0.282	1.031 (0.902,1.178)	0.656
Knee LoS	-0.024 (-0.049,0.001)	0.058	0.176 (-0.279,0.63)	0.449	-0.008 (-0.036,0.02)	0.566	0.422 (0.073,0.771)	0.018	0.15 (-0.166,0.467)	0.352	0.396 (0.015,0.777)	0.042
Knee Bed Occ	0.993 (0.984,1.002)	0.103	0.834 (0.704,0.989)	0.037	1 (0.989,1.011)	0.993	1.42 (1.297,1.556)	0.000	1.373 (1.218,1.547)	0.000	1.465 (1.312,1.635)	0.000
Knee Public Private	1.006 (0.98,1.032)	0.667	0.621 (0.358,1.077)	0.090	0.985 (0.961,1.01)	0.225	1.119 (0.915,1.369)	0.274	1.318 (1.035,1.678)	0.025	1.139 (0.931,1.394)	0.205
Elec Emerg Ratio	0.995 (0.987,1.002)	0.171	0.725 (0.64,0.821)	0.000	0.984 (0.974,0.995)	0.003	0.992 (0.887,1.109)	0.886	0.948 (0.833,1.08)	0.424	0.973 (0.86,1.1)	0.661

For peer review only - http://bmjopen.bmj.com/site/about/guidelines.xhtml

Supplementary Table T3. Interrupted time series model results with maximum auto-correlation lag 0

	pre-trend		level change		trend change		spring		summer		autumn	
	estimate (95% CI)	p	estimate (95% CI)	p	estimate (95% CI)	p	estimate (95% CI)	p	estimate (95% CI)	p	estimate (95% CI)	p
Hip Admissions	0.995 (0.985,1.005)	0.323	1.055 (0.902,1.235)	0.503	0.991 (0.978,1.005)	0.220	1.19 (1.036,1.367)	0.014	1.207 (1.088,1.339)	0.000	1.101 (0.983,1.234)	0.098
Hip Average Age	-0.015 (-0.134,0.104)	0.806	1.571 (-0.863,4.004)	0.206	-0.06 (-0.208,0.088)	0.427	1.52 (-0.079,3.119)	0.062	2.09 (0.685,3.494)	0.004	1.031 (-0.505,2.567)	0.188
Hip Prop Women	0.999 (0.993,1.004)	0.674	0.966 (0.826,1.13)	0.662	1.007 (0.997,1.017)	0.158	1.023 (0.937,1.116)	0.617	1.088 (0.995,1.189)	0.064	0.981 (0.878,1.096)	0.730
Hip Prop 2+ Charlson	0.994 (0.98,1.009)	0.455	1.411 (0.969,2.055)	0.072	1.01 (0.989,1.031)	0.344	1.195 (0.971,1.471)	0.092	1.306 (1.043,1.637)	0.020	1.003 (0.734,1.37)	0.986
Hip Prop High Deprivation	1.003 (0.991,1.014)	0.631	1.027 (0.846,1.247)	0.787	1.004 (0.983,1.025)	0.704	0.937 (0.819,1.072)	0.342	0.877 (0.746,1.03)	0.110	0.996 (0.831,1.193)	0.965
Hip LoS	-0.006 (-0.038,0.026)	0.704	0.312 (-0.262,0.887)	0.287	-0.014 (-0.058,0.03)	0.542	0.502 (0.132,0.872)	0.008	0.135 (-0.292,0.562)	0.537	0.1 (-0.287,0.487)	0.612
Hip Bed Occ	0.997 (0.987,1.009)	0.653	1 (0.824,1.215)	0.997	0.987 (0.972,1.002)	0.097	1.291 (1.125,1.48)	0.000	1.283 (1.129,1.458)	0.000	1.146 (0.984,1.334)	0.080
Hip Public Private	1.013 (0.985,1.041)	0.377	0.477 (0.29,0.782)	0.003	0.981 (0.951,1.011)	0.218	0.992 (0.809,1.217)	0.939	1.361 (1.166,1.589)	0.000	1.038 (0.847,1.273)	0.718
Knee Admissions	0.995 (0.987,1.004)	0.274	0.843 (0.702,1.013)	0.068	1.005 (0.993,1.017)	0.422	1.308 (1.154,1.483)	0.000	1.26 (1.114,1.426)	0.000	1.286 (1.147,1.441)	0.000
Knee Average Age	-0.078 (-0.157,0.001)	0.053	-1.632 (-3.299,0.035)	0.055	0.211 (0.102,0.32)	0.000	0.926 (-0.129,1.981)	0.085	0.953 (-0.257,2.163)	0.123	0.354 (-0.777,1.485)	0.540
Knee Prop Women	1.004 (0.998,1.011)	0.182	0.96 (0.84,1.097)	0.551	0.994 (0.986,1.003)	0.184	1.037 (0.939,1.146)	0.474	1.017 (0.908,1.138)	0.776	1.036 (0.928,1.157)	0.529
Knee Prop 2+ Charlson	1.009 (0.988,1.031)	0.384	0.638 (0.392,1.037)	0.070	1.042 (1.008,1.077)	0.015	1.156 (0.891,1.501)	0.276	1.074 (0.829,1.393)	0.589	0.909 (0.636,1.299)	0.600
Knee Prop High Deprivation	1.005 (0.997,1.014)	0.237	0.968 (0.784,1.195)	0.761	0.986 (0.974,0.998)	0.023	1.224 (1.099,1.363)	0.000	1.075 (0.956,1.209)	0.225	1.031 (0.911,1.166)	0.631
Knee LoS	-0.024 (-0.052,0.004)	0.089	0.176 (-0.352,0.703)	0.515	-0.008 (-0.041,0.025)	0.625	0.422 (0.117,0.726)	0.007	0.15 (-0.174,0.475)	0.364	0.396 (0.027,0.765)	0.036
Knee Bed Occ	0.993 (0.984,1.001)	0.074	0.834 (0.711,0.979)	0.027	1 (0.989,1.011)	0.993	1.42 (1.269,1.59)	0.000	1.373 (1.217,1.548)	0.000	1.465 (1.287,1.667)	0.000
Knee Public Private	1.006 (0.98,1.032)	0.667	0.621 (0.358,1.077)	0.090	0.985 (0.961,1.01)	0.225	1.119 (0.915,1.369)	0.274	1.318 (1.035,1.678)	0.025	1.139 (0.931,1.394)	0.205
Elec Emerg Ratio	0.995 (0.987,1.002)	0.167	0.725 (0.637,0.825)	0.000	0.984 (0.975,0.994)	0.001	0.992 (0.889,1.106)	0.884	0.948 (0.84,1.07)	0.392	0.973 (0.879,1.076)	0.591

For peer review only

Supplementary Table T4. Interrupted time series model results with maximum auto-correlation lag 5

	pre-trend		level change		trend change		spring		summer		autumn	
	estimate (95% CI)	p	estimate (95% CI)	p	estimate (95% CI)	p	estimate (95% CI)	p	estimate (95% CI)	p	estimate (95% CI)	p
Hip Admissions	0.995 (0.987,1.003)	0.249	1.055 (0.917,1.215)	0.454	0.991 (0.978,1.005)	0.217	1.19 (1.04,1.362)	0.012	1.207 (1.104,1.32)	0.000	1.101 (0.993,1.221)	0.069
Hip Average Age	-0.015 (-0.094,0.065)	0.714	1.571 (0.241,2.901)	0.021	-0.06 (-0.168,0.048)	0.277	1.52 (-0.178,3.218)	0.079	2.09 (0.818,3.361)	0.001	1.031 (-0.639,2.701)	0.226
Hip Prop Women	0.999 (0.995,1.002)	0.503	0.966 (0.895,1.042)	0.367	1.007 (1.001,1.014)	0.027	1.023 (0.932,1.122)	0.636	1.088 (1,1.184)	0.051	0.981 (0.879,1.094)	0.727
Hip Prop 2+ Charlson	0.994 (0.985,1.004)	0.261	1.411 (1.116,1.785)	0.004	1.01 (1,1.02)	0.042	1.195 (0.979,1.459)	0.079	1.306 (1.111,1.535)	0.001	1.003 (0.792,1.27)	0.981
Hip Prop High Deprivation	1.003 (0.994,1.012)	0.551	1.027 (0.883,1.195)	0.729	1.004 (0.99,1.018)	0.577	0.937 (0.805,1.091)	0.400	0.877 (0.752,1.022)	0.092	0.996 (0.847,1.171)	0.961
Hip LoS	-0.006 (-0.029,0.017)	0.605	0.312 (-0.12,0.745)	0.157	-0.014 (-0.042,0.015)	0.346	0.502 (0.286,0.718)	0.000	0.135 (-0.191,0.461)	0.418	0.1 (-0.332,0.532)	0.650
Hip Bed Occ	0.997 (0.987,1.008)	0.627	1 (0.862,1.161)	0.996	0.987 (0.97,1.004)	0.128	1.291 (1.116,1.492)	0.001	1.283 (1.14,1.444)	0.000	1.146 (0.99,1.326)	0.068
Hip Public Private	1.013 (0.985,1.041)	0.377	0.477 (0.29,0.782)	0.003	0.981 (0.951,1.011)	0.218	0.992 (0.809,1.217)	0.939	1.361 (1.166,1.589)	0.000	1.038 (0.847,1.273)	0.718
Knee Admissions	0.995 (0.992,0.999)	0.016	0.843 (0.761,0.934)	0.001	1.005 (0.998,1.012)	0.170	1.308 (1.154,1.482)	0.000	1.26 (1.16,1.369)	0.000	1.286 (1.178,1.403)	0.000
Knee Average Age	-0.078 (-0.152,-0.005)	0.037	-1.632 (-2.825,-0.439)	0.007	0.211 (0.128,0.293)	0.000	0.926 (-0.072,1.924)	0.069	0.953 (0.077,1.829)	0.033	0.354 (-0.422,1.13)	0.371
Knee Prop Women	1.004 (0.999,1.009)	0.086	0.96 (0.861,1.071)	0.465	0.994 (0.987,1.001)	0.097	1.037 (0.973,1.105)	0.260	1.017 (0.922,1.121)	0.743	1.036 (0.966,1.111)	0.319
Knee Prop 2+ Charlson	1.009 (0.993,1.026)	0.250	0.638 (0.468,0.869)	0.004	1.042 (1.021,1.064)	0.000	1.156 (0.931,1.436)	0.190	1.074 (0.841,1.372)	0.567	0.909 (0.626,1.321)	0.617
Knee Prop High Deprivation	1.005 (0.999,1.011)	0.107	0.968 (0.835,1.122)	0.664	0.986 (0.977,0.995)	0.003	1.224 (1.082,1.384)	0.001	1.075 (0.95,1.217)	0.251	1.031 (0.924,1.149)	0.586
Knee LoS	-0.024 (-0.047,-0.001)	0.042	0.176 (-0.2,0.551)	0.360	-0.008 (-0.031,0.015)	0.490	0.422 (0.033,0.81)	0.033	0.15 (-0.199,0.499)	0.398	0.396 (-0.011,0.803)	0.057
Knee Bed Occ	0.993 (0.986,0.999)	0.032	0.834 (0.728,0.956)	0.009	1 (0.991,1.009)	0.991	1.42 (1.308,1.542)	0.000	1.373 (1.213,1.553)	0.000	1.465 (1.318,1.627)	0.000
Knee Public Private	1.006 (0.98,1.032)	0.667	0.621 (0.358,1.077)	0.090	0.985 (0.961,1.01)	0.225	1.119 (0.915,1.369)	0.274	1.318 (1.035,1.678)	0.025	1.139 (0.931,1.394)	0.205
Elec Emerg Ratio	0.995 (0.987,1.003)	0.198	0.725 (0.635,0.828)	0.000	0.984 (0.974,0.995)	0.004	0.992 (0.898,1.095)	0.872	0.948 (0.835,1.077)	0.414	0.973 (0.849,1.114)	0.690

For peer review only

For peer review only - http://bmjopen.bmj.com/site/about/guidelines.xhtml

The RECORD statement – checklist of items, extended from the STROBE statement, that should be reported in observational studies using routinely collected health data.

	Item No.	STROBE items	Location in manuscript where items are reported	RECORD items	Location in manuscript where items are reported
**Title and abstract**					
	1	(a) Indicate the study's design with a commonly used term in the title or the abstract (b) Provide in the abstract an informative and balanced summary of what was done and what was found		RECORD 1.1: The type of data used should be specified in the title or abstract. When possible, the name of the databases used should be included.<br><br>RECORD 1.2: If applicable, the geographic region and timeframe within which the study took place should be reported in the title or abstract.<br><br>RECORD 1.3: If linkage between databases was conducted for the study, this should be clearly stated in the title or abstract.	Abstract P2 L35-37<br><br>Abstract P2 L35-38
**Introduction**					
Background rationale	2	Explain the scientific background and rationale for the investigation being reported			Introduction P4-5 L76-113
Objectives	3	State specific objectives, including any prespecified hypotheses			Introduction P5 L107-113
**Methods**					
Study Design	4	Present key elements of study design early in the paper			Methods P5 L116-119
Setting	5	Describe the setting, locations, and relevant dates, including periods of recruitment, exposure, follow-up, and data collection			Methods P5 L116-119 Methods P7-8 L70-176

For peer review only

Participants	6	*(a) Cohort study* - Give the eligibility criteria, and the sources and methods of selection of participants. Describe methods of follow-up<br>*Case-control study* - Give the eligibility criteria, and the sources and methods of case ascertainment and control selection. Give the rationale for the choice of cases and controls<br>*Cross-sectional study* - Give the eligibility criteria, and the sources and methods of selection of participants<br><br>*(b) Cohort study* - For matched studies, give matching criteria and number of exposed and unexposed<br>*Case-control study* - For matched studies, give matching criteria and the number of controls per case		RECORD 6.1: The methods of study population selection (such as codes or algorithms used to identify subjects) should be listed in detail. If this is not possible, an explanation should be provided.<br><br>RECORD 6.2: Any validation studies of the codes or algorithms used to select the population should be referenced. If validation was conducted for this study and not published elsewhere, detailed methods and results should be provided.<br><br>RECORD 6.3: If the study involved linkage of databases, consider use of a flow diagram or other graphical display to demonstrate the data linkage process, including the number of individuals with linked data at each stage.	Methods P6<br>L141-147<br>Supplementary Table T1
Variables	7	Clearly define all outcomes, exposures, predictors, potential confounders, and effect modifiers. Give diagnostic criteria, if applicable.		RECORD 7.1: A complete list of codes and algorithms used to classify exposures, outcomes, confounders, and effect modifiers should be provided. If these cannot be reported, an explanation should be provided.	Methods P6-7<br>L141-169
Data sources/ measurement	8	For each variable of interest, give sources of data and details of methods of assessment (measurement).<br>Describe comparability of assessment methods if there is more than one group			Methods P5-6<br>L120-140

For peer review only

Bias	9	Describe any efforts to address potential sources of bias			Methods P7-8 L170-185,
Study size	10	Explain how the study size was arrived at			Methods P6 L141-147
Quantitative variables	11	Explain how quantitative variables were handled in the analyses. If applicable, describe which groupings were chosen, and why			Methods P6-7 L141-169
Statistical methods	12	(a) Describe all statistical methods, including those used to control for confounding<br>(b) Describe any methods used to examine subgroups and interactions<br>(c) Explain how missing data were addressed<br>(d) *Cohort study* - If applicable, explain how loss to follow-up was addressed<br>*Case-control study* - If applicable, explain how matching of cases and controls was addressed<br>*Cross-sectional study* - If applicable, describe analytical methods taking account of sampling strategy<br>(e) Describe any sensitivity analyses			Methods P7-8 L170-185
Data access and cleaning methods		..		RECORD 12.1: Authors should describe the extent to which the investigators had access to the database population used to create the study population.	Data Sharing P17 L392-400

For peer review only

				RECORD 12.2: Authors should provide information on the data cleaning methods used in the study.	Methods P6-7 L141-169
Linkage		..		RECORD 12.3: State whether the study included person-level, institutional-level, or other data linkage across two or more databases. The methods of linkage and methods of linkage quality evaluation should be provided.	N/A
**Results**					
Participants	13	(a) Report the numbers of individuals at each stage of the study (*e.g.*, numbers potentially eligible, examined for eligibility, confirmed eligible, included in the study, completing follow-up, and analysed)<br>(b) Give reasons for non-participation at each stage.<br>(c) Consider use of a flow diagram		RECORD 13.1: Describe in detail the selection of the persons included in the study (*i.e.,* study population selection) including filtering based on data quality, data availability and linkage. The selection of included persons can be described in the text and/or by means of the study flow diagram.	Methods P6 L141-147<br><br>Results P8 L191-194
Descriptive data	14	(a) Give characteristics of study participants (*e.g.*, demographic, clinical, social) and information on exposures and potential confounders<br>(b) Indicate the number of participants with missing data for each variable of interest<br>(c) *Cohort study* - summarise follow-up time (*e.g.*, average and total amount)			Results P8 L191-194
Outcome data	15	*Cohort study* - Report numbers of outcome events or summary measures over time<br>*Case-control study* - Report numbers in each exposure			Results P8-9 L195-206

For peer review only

		category, or summary measures of exposure<br>*Cross-sectional study* - Report numbers of outcome events or summary measures			
Main results	16	(a) Give unadjusted estimates and, if applicable, confounder-adjusted estimates and their precision (e.g., 95% confidence interval). Make clear which confounders were adjusted for and why they were included<br>(b) Report category boundaries when continuous variables were categorized<br>(c) If relevant, consider translating estimates of relative risk into absolute risk for a meaningful time period			Results P9-11 L196-267
Other analyses	17	Report other analyses done—e.g., analyses of subgroups and interactions, and sensitivity analyses			Results P9-11 L196-267
**Discussion**					
Key results	18	Summarise key results with reference to study objectives			Discussion P11-12, L268-288
Limitations	19	Discuss limitations of the study, taking into account sources of potential bias or imprecision. Discuss both direction and magnitude of any potential bias		RECORD 19.1: Discuss the implications of using data that were not created or collected to answer the specific research question(s). Include discussion of misclassification bias, unmeasured confounding, missing data, and changing eligibility over time, as they pertain to the study being reported.	Discussion P12-13 L289-303
Interpretation	20	Give a cautious overall interpretation of results considering objectives,			Discussion P13-14 L317-340

		limitations, multiplicity of analyses, results from similar studies, and other relevant evidence			
Generalisability	21	Discuss the generalisability (external validity) of the study results			Discussion P13 L294-303
**Other Information**					
Funding	22	Give the source of funding and the role of the funders for the present study and, if applicable, for the original study on which the present article is based			Funding Statement P16 L375-380
Accessibility of protocol, raw data, and programming code		..		RECORD 22.1: Authors should provide information on how to access any supplemental information such as the study protocol, raw data, or programming code.	Methods P8 L186-188 Data Sharing P17 L392-400

*Reference: Benchimol EI, Smeeth L, Guttmann A, Harron K, Moher D, Petersen I, Sørensen HT, von Elm E, Langan SM, the RECORD Working Committee. The REporting of studies Conducted using Observational Routinely-collected health Data (RECORD) Statement. *PLoS Medicine* 2015; in press.

*Checklist is protected under Creative Commons Attribution (CC BY) license.

For peer review only

The impact of pausing elective hip and knee replacement surgery during winter 2017 on subsequent service provision at a major NHS Trust: a descriptive observational study using interrupted time series

Journal:	*BMJ Open*
Manuscript ID	bmjopen-2022-066398.R1
Article Type:	Original research
Date Submitted by the Author:	31-Jan-2023
Complete List of Authors:	Jones, Tim; University Hospitals Bristol and Weston NHS Foundation Trust, NIHR ARC West; University of Bristol, Department of Population Health Sciences<br>Penfold, Chris; University Hospitals Bristol and Weston NHS Foundation Trust, NIHR ARC West; University of Bristol, Musculoskeletal Research Unit<br>Redaniel, Maria Theresa; University Hospitals Bristol and Weston NHS Foundation Trust, NIHR ARC West; University of Bristol, Population Health Sciences, Bristol Medical School<br>Eyles, Emily; University Hospitals Bristol and Weston NHS Foundation Trust, NIHR ARC West; University of Bristol, Population Health Sciences, Bristol Medical School<br>Keen, Tim; North Bristol NHS Trust<br>Elliott, Andrew; North Bristol NHS Trust<br>Blom, AW; University of Bristol, Translational Health Sciences, Bristol Medical School<br>Judge, Andrew; University of Bristol, Musculoskeletal Research Unit
**Primary Subject Heading**:	Health services research
Secondary Subject Heading:	Surgery
Keywords:	Knee < ORTHOPAEDIC & TRAUMA SURGERY, Hip < ORTHOPAEDIC & TRAUMA SURGERY, Orthopaedic & trauma surgery < SURGERY, Human resource management < HEALTH SERVICES ADMINISTRATION & MANAGEMENT

I, the Submitting Author has the right to grant and does grant on behalf of all authors of the Work (as defined in the below author licence), an exclusive licence and/or a non-exclusive licence for contributions from authors who are: i) UK Crown employees; ii) where BMJ has agreed a CC-BY licence shall apply, and/or iii) in accordance with the terms applicable for US Federal Government officers or employees acting as part of their official duties; on a worldwide, perpetual, irrevocable, royalty-free basis to BMJ Publishing Group Ltd ("BMJ") its licensees and where the relevant Journal is co-owned by BMJ to the co-owners of the Journal, to publish the Work in this journal and any other BMJ products and to exploit all rights, as set out in our licence.

The Submitting Author accepts and understands that any supply made under these terms is made by BMJ to the Submitting Author unless you are acting as an employee on behalf of your employer or a postgraduate student of an affiliated institution which is paying any applicable article publishing charge ("APC") for Open Access articles. Where the Submitting Author wishes to make the Work available on an Open Access basis (and intends to pay the relevant APC), the terms of reuse of such Open Access shall be governed by a Creative Commons licence – details of these licences and which Creative Commons licence will apply to this Work are set out in our licence referred to above.

Other than as permitted in any relevant BMJ Author's Self Archiving Policies, I confirm this Work has not been accepted for publication elsewhere, is not being considered for publication elsewhere and does not duplicate material already published. I confirm all authors consent to publication of this Work and authorise the granting of this licence.

For peer review only

Title: The impact of pausing elective hip and knee replacement surgery during winter 2017 on subsequent service provision at a major NHS Trust: a descriptive observational study using interrupted time series

Authors: Tim Jones[1,2,3], Christopher Penfold[1,2,3], Maria Theresa Redaniel[1,2], Emily Eyles[1,2], Tim Keen[4], Andrew Elliott[4], Ashley Blom[2,3], Andrew Judge[3,5]

[1] NIHR ARC West, University Hospitals Bristol NHS Foundation Trust, Bristol, BS1 2NT, UK

[2] Department of Population Health Sciences, Bristol Medical School, University of Bristol, BS8 2PS, UK

[3] Musculoskeletal Research Unit, Translational Health Sciences, Bristol Medical School, University of Bristol, Learning and Research Building, Level 1, Southmead Hospital, Bristol, BS10 5NB, UK

[4] North Bristol NHS Trust, Southmead Hospital, Westbury-on-Trym, Bristol, BS10 5NB, UK

[5] National Institute for Health Research Bristol Biomedical Research Centre, University Hospitals Bristol and Weston NHS Foundation Trust and University of Bristol, Bristol, UK

Corresponding author:

Tim Jones
Research Fellow
NIHR ARC West,
9th Floor Whitefriars,
Lewin's Mead
Bristol, BS1 2NT
United Kingdom

Tel: ++44 (0)117 342 1265
Timothy.Jones@bristol.ac.uk

Word count [3,826]

For peer review only

ABSTRACT

Objectives: To explore the impact of a temporary cancellation of elective surgery in winter 2017 on trends in primary hip and knee replacement at a major NHS Trust, and whether lessons can be learnt about efficient surgery provision.

Design and Setting: Observational descriptive study using interrupted time series analysis of hospital records to explore trends in primary hip and knee replacement surgery at a major NHS Trust, as well as patient characteristics, 2016-2019.

Intervention: A temporary cancellation of elective services for two months in winter 2017

Outcomes: NHS-funded hospital admissions for primary hip or knee replacement, length of stay and bed occupancy. Additionally, we explored the ratio of elective to emergency admissions at the Trust as a measure of elective capacity, and the ratio of public to private provision of NHS-funded hip and knee surgery.

Results: After winter 2017 there was a sustained reduction in the number of knee replacements, a decrease in the proportion of most deprived people having knee replacements, and an increase in average age for knee replacement and comorbidity for both types of surgery. The ratio of public to private provision dropped after winter 2017, and elective capacity generally has reduced over time. There was clear seasonality in provision of elective surgery, with less-complex patients admitted during winter.

Conclusions: Declining elective capacity and seasonality has a marked effect on the provision of joint replacement, despite efficiency improvements in hospital treatment. The Trust has outsourced less complex patients to independent providers, and/or treated them during winter when capacity is most limited. There is a need to explore whether these are strategies that could be used explicitly to maximise the use of limited elective capacity, provide benefit to patients, and value for money for taxpayers.

For peer review only - http://bmjopen.bmj.com/site/about/guidelines.xhtml

STRENGTH AND LIMITATIONS OF THIS STUDY

- Trends analyses using data obtained from the electronic health records of a local hospital NHS Trust are informative for clinicians and service managers in monitoring changes in planning and delivery of elective surgery, and could be regularly updated in near real time for monitoring.
- The inclusion of wider hospital admissions data beyond the NHS Trust allows us to estimate the proportion of people within the Trust catchment area having NHS-funded treatment at independent providers.
- We report the experience of one NHS Trust that is one of the larger elective orthopaedic centres - the findings may not be generalisable to or reflect the experience of other trusts.
- Our study does not include privately funded, privately provided hip and knee surgery which may also have been changing over time.

For peer review only

For peer review only - http://bmjopen.bmj.com/site/about/guidelines.xhtml

INTRODUCTION

Primary hip and knee replacement operations are common planned elective surgical procedures. They are highly clinically effective for improving symptoms of pain and functional limitations, and have been shown to be safe and cost-effective.[1-4] Around 100,000 hip[4] and over 100,000 knee operations[3] are carried out each year in the UK. Demand for these operations has been increasing substantially in recent decades[5] with an ageing population, rising levels of obesity, and widening indications for surgery in younger patient groups.[3 4]

Orthopaedic services have become more efficient over time, with length of hospital stay for primary hip and knee replacements reducing from around 15 days in 1997 to roughly 5.5 days in 2014.[6] This is largely due to the introduction of 'fast track' surgery and enhanced recovery services,[7] which reduce length of stay whilst maintaining patient safety and outcomes of surgery.[6] However, over the past decade there has also been a reduction in the numbers of hospital beds and operating theatres available for hip and knee replacement patients.[8] Waiting lists for orthopaedic procedures have been growing over time, and the average time people wait for treatment once on the waiting list has also increased.[9]

Pressures on elective surgery are exacerbated during winter, when resources for planned surgery are often displaced by more acute, unplanned hospital admissions.[8] At the end of 2017, this led to all planned elective hip and knee replacement operations in England being cancelled for the whole of January.[10] Even before the COVID-19 pandemic, over half a million people were already on the waiting list.[11] Patients are having to wait longer with deteriorating severe pain and functional limitation, affecting their health and quality of life. The COVID-19 pandemic has had an even greater impact on cancelling planned elective surgery, with over 635,000 people waiting for hip and knee replacements in April 2021, more than 10% of these waiting over a year, and over a third waiting longer than the 18 week target.[11]

For peer review only

99 The winter of 2017 provides a form of 'natural experiment', where elective capacity was intentionally reduced close to zero. A natural experimental design is a valid methodological approach to evaluate the impact of a range of events, policies and interventions which are not under the control of researchers.[12] Researchers can use the variation in exposure that natural experiments generate to analyse their impact on health outcomes. This provides a form of quasi-experimental study, where we can explore trends in provision of elective surgery before and after Winter 2017, which is a robust approach to explore real-world impact when randomisation is not possible.[13 14]

106 Our aim was to understand what happens after common, planned elective surgery is temporarily cancelled, and how this might inform optimum planning of elective surgery when capacity is limited, such as following the COVID-19 pandemic. We used interrupted time series analysis to model trends in elective hip and knee replacement surgery for a major NHS Trust from 2016 to 2019 and see how these were impacted by the withdrawal of elective surgery in winter 2017. We explored these trends by patient factors (age, sex, deprivation, number of comorbidities) and seasonality to see when demand was highest for different patient groups.

113

114 **METHODS**

115 This study is a longitudinal observational descriptive study using routinely collected administrative information about patients admitted to a major NHS Trust for elective hip and knee replacements, 2016 to 2019. It was developed and reported according to the RECORD extension[15] to STROBE guidelines for observational studies using routinely collected data.

119 **Data Sources**

120 We used two data sources for our analyses. The first was an extract of elective primary hip and knee replacement inpatient admissions identified from the Trust's electronic medical records (EMR) between 1st January 2016 and 31st December 2019. Up to 29 diagnoses were provided per entry using the International Classification of Diseases version 10 (ICD-10), and up to 11 procedures were provided

For peer review only - http://bmjopen.bmj.com/site/about/guidelines.xhtml

per entry using the Office of Population Censuses and Surveys Classification of Interventions and Procedures version 4 (OPCS-4). The extract included patient demographics such as age, sex, deprivation quintile, and comorbidities; and other characteristics of the hospital admissions such as length of stay. This data source was used for all analyses of hip and knee replacements at the Trust, including those relating to patient demographics, length of stay, and bed occupancy.

The second data source was pseudonymised national admitted patient care Hospital Episode Statistics (HES-APC) between 1st January 2016 and 31st December 2019. HES-APC is a routinely collected dataset that records all episodes of admitted (day case or inpatient) care provided to patients at NHS hospitals in England and to NHS-funded patients treated in independent hospitals.[16] Each episode represents a period of care under one consultant team. Up to 20 diagnoses and 24 clinical procedures are recorded per episode using ICD-10 codes and OPCS-4 codes, respectively. HES also includes the Lower Super Output Area (LSOA; an area of around 1,500 people) of residence for each patient, which can be linked to CCG of residence. This data source was used to estimate elective capacity overall at the Trust, and the ratio of public/private provision of hip and knee replacements in the catchment area for the Trust (see details below), which could not be gathered from the extract provided from the Trust EMR.

Hospital admissions for hip and knee replacements

Hospital admissions for elective hip and knee replacements were identified by entries with a primary procedure code representing primary hip or knee replacement (Supplementary Table T1) using the Trust EMR. We used this information to explore summary characteristics of the hospital admissions over time (overall counts of admissions, average age, proportion of women, proportion with 2+ comorbidities, proportion in the two most deprived quintiles) stratified by primary hip or knee replacements.

Length of stay and bed occupancy

For peer review only - http://bmjopen.bmj.com/site/about/guidelines.xhtml

We used the average number of overnight stays in hospital (days) for length of stay, trimmed at 30 days to exclude a small number of outliers (n=32, 0.6%). Trimming allowed us to model averages assuming a roughly normal distribution which we felt was more easily interpretable. Bed occupancy was the total number of beds used overnight for hip and knee replacement patients.

Comorbidity of Admissions

For each admission, we counted the number of conditions from the Charlson comorbidity index[17] recorded in the diagnosis fields. The Charlson index provides a summary of weighted scores relating to different comorbidities and has been shown to be associated with mortality. Admissions were categorised into those with zero, one, and two or more Charlson comorbidities.

Ratio of Elective to Emergency Admissions

To estimate the ratio of elective to emergency admissions for all purposes at the Trust (as a proxy for elective capacity), we extracted all hospital admissions from HES-APC with the Trust as a provider and categorised them into elective and emergency (admission method beginning with '1' or '2', respectively).

Ratio of Public to Private Provision of Hip and Knee Replacements

To estimate the ratio of public to private provision of NHS-funded elective hip and knee surgery for the Trust catchment area, we extracted all hospital admissions for primary hip and knee replacements (codes in Supplementary Table T1) for residents of the major local clinical commissioning groups (CCGs) from HES-APC (using 2021 CCG boundaries after local CCGs had merged into one CCG[18]), and categorised providers into public and private (provider code beginning with 'R' or 'N', respectively).

Statistical Analysis

We explored the change in trend for the following outcomes before/after the winter 2017 cancellation of elective surgery, stratified by primary hip and knee replacements: number of hospital admissions;

For peer review only - http://bmjopen.bmj.com/site/about/guidelines.xhtml

average age of patients; proportion of women; proportion with 2+ comorbidities; proportion in more deprived deprivation quintiles (4 and 5); average length of stay; bed occupancy; and ratio of public to private provision of surgery. Additionally, we explored the overall ratio of elective to emergency admissions at the hospital for any purpose without stratification. For each of the outcomes, we conducted interrupted time series (ITS) analyses using segmented regression models comparing hospital admissions in the 'before' period (January 2016 to November 2017) to the 'after' period (February 2018 to December 2019). We excluded the winter 2017 period when admissions were very low (December 2017 and January 2018). The ITS analyses explored the 'pre-trend' before winter 2017, and how this trend changed after winter 2017[12 19], allowing for an immediate 'level change' up or down in February 2018, and a longer-term 'trend change' in the slope afterwards. We explored seasonality in the data by including indicator variables for spring, summer, and autumn[19] compared to winter as a baseline, and adjusted for serial autocorrelation using Newey-West standard errors with a maximum lag of two[20-22]. For count or proportion outcomes (number of admissions, proportion women, proportion with 2+ comorbidities, proportion in top two deprivation quintiles, bed occupancy) segmented Poisson regression models were fit to the data, whilst for averages/ratios (average age, average length of stay, ratio of elective to emergency admissions, ratio of public to private provision) segmented linear regression models were fit, using the 'glm' command in Stata. Sensitivity analyses were conducted adjusting the maximum lag for serial autocorrelation to zero and five; this would not affect point estimates but could alter standard errors, confidence intervals, and p-values.

All statistical analyses were conducted using Stata/MP version 16.1. Smoothed trends were fit to the data on all plots using the 'lowess' command with bandwidth 0.3. Stata code is available at: https://github.com/jonestim2002/hdr_uk_hospital_efficiency

Patient and public involvement

For peer review only - http://bmjopen.bmj.com/site/about/guidelines.xhtml

Initial research ideas for the grant application of which this work is part were presented to the public in a workshop and suggestions and comments were incorporated in the protocol. Feedback during the workshop was positive, with participants agreeing with the research objectives and the identified need.

RESULTS

Descriptive information and demographics

A total of 2,623 patients had a hip replacement and 2,674 had a knee replacement at the Trust in the 4 years between 2016 and 2019. The mean age of patients was 67 years and 60% were women for both types of operations.

Trend changes after winter 2017

Table 1 shows the results of our interrupted time series analyses for all outcomes, including the trend before winter 2017 (pre-trend), any immediate change after winter 2017 (level change) and any change in the slope after winter 2017 (trend change). These are described in more detail below.

Table 1. Interrupted time series model results

	pre-trend		level change		trend change	
	estimate (95% CI)	p	estimate (95% CI)	p	estimate (95% CI)	p
Hip Admissions	1 (0.99,1)	0.300	1.06 (0.91,1.22)	0.469	0.99 (0.98,1.01)	0.239
Hip Age*	-0.01 (-0.1,0.07)	0.737	1.57 (-0.1,3.24)	0.065	-0.06 (-0.17,0.05)	0.307
Hip Prop Women	1 (0.99,1)	0.582	0.97 (0.86,1.08)	0.549	1.01 (1,1.02)	0.089
Hip Charlson	0.99 (0.98,1.01)	0.380	1.41 (1.06,1.87)	0.017	1.01 (0.99,1.03)	0.220
Hip Deprivation	1 (0.99,1.01)	0.587	1.03 (0.87,1.21)	0.754	1 (0.99,1.02)	0.660
Hip LoS*	-0.01 (-0.03,0.02)	0.660	0.31 (-0.19,0.82)	0.225	-0.01 (-0.05,0.02)	0.425
Hip Bed Occ	1 (0.99,1.01)	0.643	1 (0.84,1.19)	0.997	0.99 (0.97,1)	0.149
Hip Public Private*	0.01 (-0.02,0.04)	0.377	-0.74 (-1.24,-0.25)	0.003	-0.02 (-0.05,0.01)	0.218
Knee Admissions	1 (0.99,1)	0.106	0.84 (0.73,0.98)	0.022	1 (1,1.01)	0.256
Knee Age*	-0.08 (-0.16,0)	0.054	-1.63 (-2.99,-0.28)	0.018	0.21 (0.12,0.31)	0.000

For peer review only - http://bmjopen.bmj.com/site/about/guidelines.xhtml

For peer review only

Knee Prop Women	1 (1,1.01)	0.150	0.96 (0.85,1.08)	0.513	0.99 (0.99,1)	0.193
Knee Charlson	1.01 (0.99,1.03)	0.249	0.64 (0.46,0.89)	0.009	1.04 (1.02,1.07)	0.001
Knee Deprivation	1.01 (1,1.01)	0.189	0.97 (0.79,1.19)	0.758	0.99 (0.97,1)	0.021
Knee LoS*	-0.02 (-0.05,0)	0.058	0.18 (-0.28,0.63)	0.449	-0.01 (-0.04,0.02)	0.566
Knee Bed Occ	0.99 (0.98,1)	0.103	0.83 (0.7,0.99)	0.037	1 (0.99,1.01)	0.993
Knee Public Private*	0.01 (-0.02,0.03)	0.667	-0.48 (-1.03,0.07)	0.090	-0.02 (-0.04,0.01)	0.225
Elec Emerg Ratio*	-0.01 (-0.01,0)	0.171	-0.32 (-0.45,-0.2)	0.000	-0.02 (-0.03,-0.01)	0.003

*Notes: *Linear regression model (additive) rather than Poisson regression model (multiplicative). Shaded cells indicate p < 0.05.*

Trends in hip and knee elective hospital admissions over time

The overall numbers of elective primary hip and knee replacement operations gradually declined over the study period from 63 hip and 65 knee replacements per month in 2016 to 49 hip and 51 knee replacements per month in 2019. Whilst there was a drop off in winter 2017, after elective surgery was re-started hip replacements resumed at similar numbers and continued to decline along a similar trajectory. Numbers of knee replacements dropped by 16% after winter 2017 (level change=0.843, 95% CI: 0.728 to 0.976, p=0.022), and the slope appeared to level off, although there was little evidence for this in the regression model (trend change=1.005, 95% CI: 0.996 to 1.014, p=0.256); see Figure 1 and Supplementary Table T2.

Age on Admission

There was a change in the trend in average age for knee replacements after winter 2017 (trend change=+0.21, 95% CI: 0.12 to 0.31, p < 0.001) towards treating older patients over time (+1.59 years of age per year); see Figure 2.

Comorbidity of Admissions

There was a level change upwards in the proportion having hip replacements with 2+ comorbidities after winter 2017 (level change=1.411, 95% CI: 1.064 to 1.873, p=0.017), and an upward slope change for knee replacements (trend change=1.042, 95% CI: 1.017 to 1.067, p=0.001); see Figure 3.

For peer review only - http://bmjopen.bmj.com/site/about/guidelines.xhtml

Deprivation

There was evidence of a reducing proportion of the most deprived people having knee replacements after winter 2017 (trend change=0.986, 95% CI: 0.974 to 0.998, p=0.021).

Ratio of elective admissions to emergency admissions at the Trust

There was an overall downward trend in the ratio of elective to emergency admissions at the Trust, from an average of 2.91 (SD: 0.17) electives for every emergency in 2016 to 2.16 (SD: 0.06) in 2019; see Supplementary Figure F1. The ratio reduced after winter 2017 (level change=-0.322, 95% CI: -0.446 to -0.198, p<0.001), and started to decrease more rapidly afterwards (trend change=-0.016, 95% CI: -0.026 to -0.005, p=0.003).

Ratio of public to private provision of hip/knee elective surgery at the Trust

There was evidence of a level change downwards in public provision compared to private provision after winter 2017 for both types of surgery, but particularly for hip replacements (hips level change=-0.741, 95% CI: -1.237 to -0.245, p=0.003; knees level change=-0.476, 95% CI: -1.026 to +0.074, p=0.09); see Figure 4.

Bed Occupancy

For hip and knee replacements, bed occupancy has reduced over time, although there wasn't evidence of this in the regression model for hip replacements, and there was a level change downwards (level change=0.834, 95% CI: 0.704 to 0.989, p=0.037) for knee surgery after winter 2017; see Figure 5.

For peer review only - http://bmjopen.bmj.com/site/about/guidelines.xhtml

Length of Stay

The average length of hospital stay was 5.5 days (SD: 5.9 days) for hip replacements and 5.2 days (SD: 5.0 days) for knee replacements in 2016, compared to 5.1 days (SD: 4.1 days) and 4.3 days (SD: 3.4 days) respectively in 2019 (see Supplementary Figure F2). However, there was no evidence in the regression models for a change after winter 2017.

Seasonality

Supplementary Table T2 shows seasonality results for each of our interrupted time series analyses. Hip and knee operations were clearly seasonal, with higher admissions in non-winter months compared to winter; 21% higher in the highest season (summer) for hips (summer=1.207, 95% CI: 1.094 to 1.332, p<0.001), and 31% higher in the highest season (spring) for knee replacements (spring=1.308, 95% CI: 1.157 to 1.479, p<0.001), excluding winter 2017. Bed occupancy for both types of operation was also seasonal, with lower occupancy in the winter months compared to all other seasons (see Supplementary Table T2); e.g. summer bed occupancy was 324 beds for hips and 291 beds for knees on average compared to winter bed occupancy of 225 beds for hips and 199 beds for knees on average. Length of stay was longer in spring than winter for hip replacements (spring=+0.502 days, 95% CI: 0.214 to 0.79, p=0.001), and longer in spring (+0.422 days, 95% CI: 0.073 to 0.771, p=0.018) and autumn (+0.396 days, 95% CI: 0.015 to 0.777, p=0.042) compared to winter for knee replacements.

The ratio of public to private provision was higher in the summer (1.56 for hips and 1.28 for knees) compared to winter (1.22 and 0.99, respectively) months (hips summer=+0.308, 95% CI 0.154 to 0.463, p<0.001; knees summer=+0.276, 95% CI: 0.035 to 0.517, p=0.025).

There was also some evidence of seasonality in the types of patients being admitted for hip and knee replacements. For hip replacements, the mean age of patients was 66 in winter compared to

For peer review only

68 in summer (summer=+2.09; 95% CI: 0.81 to 3.37, p = 0.001); a higher proportion were performed on women in the summer (64%) compared to winter (58%) months (summer=1.088, 95% CI: 1.001 to 1.183, p=0.048); and a higher proportion of people had 2+ comorbidities in the summer (15.9%) compared to winter (12.3%) months (summer=1.306, 95% CI: 1.096 to 1.557, p=0.003). For knee replacements, there was a higher proportion of more deprived people (quintiles 4 and 5) in the spring (37.6%) compared to the winter (30.2%) months (spring=1.224, 95% CI: 1.077 to 1.49, p=0.002).

DISCUSSION

Principal findings

The temporary cancellation of elective services during winter 2017 does appear to have had some impact on service provision at the Trust after that time. There was an immediate and sustained reduction in the number of knee replacements being done at the Trust and this was also reflected in the drop in bed occupancy for knee surgery. The average age for knee replacement and comorbidity of hip and knee surgery patients increased after winter 2017, whilst the proportion of more deprived people having knee replacements decreased, and the ratio of public to private provision of hip and knee replacements in the local area dropped after winter 2017. This suggests an NHS-funded outsourcing of less comorbid hip and knee replacement surgery to independent providers, and therefore on average the patients being treated at the Trust became older and more comorbid. There was a general decrease in capacity for elective surgery at the Trust (ratio of elective to emergency admissions), mostly driven by increasing non-elective admissions even before the COVID-19 pandemic. The winter 2017 cancellation may have been just one symptom of this overall pressure on elective surgery that underlies some of the longer-term changes in provision.

For peer review only

There was also some seasonality in service provision. It is no surprise that elective admissions and bed occupancy are lower in winter when the hospital requires capacity for an increase in unplanned admissions. There were also indications that people being admitted in winter were younger, less comorbid, and less deprived (particularly for knee surgery). Length of stay for hip and knee replacements was lower in winter compared to spring. This suggests the admission of younger, less comorbid patients during the winter months given the reduced elective capacity and delaying surgery for more comorbid patients to when capacity is higher in the following months.

Strengths and limitations

Trends analyses such as these, using data obtained from the EHR of a local hospital NHS Trust, are informative for clinicians and service managers in monitoring changes in planning and delivery of elective surgery, and could be regularly updated in near real time for monitoring. This concept might be informative for other commissioning groups / Trusts to adopt for monitoring of their own elective surgery and capacity. We report the experience of just one trust that is one of the larger elective orthopaedic centres, and hence the findings may not be generalisable to or reflect the experience of other trusts. Our findings are observational and report changes observed at the Trust following cancellation of elective services in winter 2017; further work would be needed to understand the impact of any changes on outcomes such as throughput of patients, waiting times, waiting lists, outcomes of surgery, costs, and equity of access to surgery. We should be aware that some results may reflect chance findings due to multiple testing and type 1 error. The trends in the data as plotted do not change substantially in sensitivity analyses accounting for different autocorrelation lags (Supplementary Tables T3-T4). The catchment area of the Trust is not exactly the same as the major local CCG and is difficult to define exactly. However, 89.4% of admissions at the Trust were for residents of the local CCG and we felt this was a reasonable approximation to estimate the ratio of public to private provision in the Trust catchment area. Our analyses only include NHS-funded surgery and not privately-funded, privately-provided surgery.

For peer review only - http://bmjopen.bmj.com/site/about/guidelines.xhtml

Comparison to other studies

A previous study[23] using data for England from Hospital Episode Statistics found increasing private provision of elective hip arthroplasties nationally from 2007/8 to 2012/13, particularly for less deprived people, which echoes our findings. More recent news stories have suggested that 20% of NHS-funded hip replacements and 29% of NHS-funded knee replacements were carried out by independent providers in 2016/17[24], and that independently-provided hip and knee replacement surgery (privately or NHS-funded) has now overtaken NHS provision.[25] A UK-wide study[6] using primary care data (CPRD) linked to hospital admissions found similar effects of patient characteristics (age, sex, comorbidity, and deprivation) on length of stay for primary hip and knee replacements, although they did not explore seasonality. A recent qualitative study[26] highlighted the negative financial and emotional impact of winter elective cancellations on patients and their families and recommended better advanced planning of elective operations to reduce these impacts.

Implications for clinicians and policy makers

Outsourcing of less complex hip and knee replacements to take advantage of spare capacity in non-NHS hospitals may be a good strategy to reduce waiting times and waiting lists for surgery and get the best results for patients given the evident capacity limitations. However, this would leave the NHS Trust to cope with more complex cases and has training implications because trainee surgeons are usually trained by first undertaking less-complex cases on healthier patients. There are also potential equity implications, if less complex cases have the option of surgery with shorter waiting times at independent providers, whilst more complex (and potentially more deprived) cases do not. We would need to consider the acceptability of this outsourcing to patients and practitioners, and the quality of patient outcomes.

For peer review only

There is an indication that some selection of patients for elective surgery depending on available capacity already takes place at the Trust. It is possible that this could become a more explicit strategy, based on evidence, to optimise the use of limited capacity in hospitals at different times of the year. However, this could mean that people placed earlier on the waiting list for surgery might get their surgery later due to such scheduling strategies, so acceptability to patients would need to be explored. We need to understand how the scheduling and possible outsourcing of elective surgery for different types of patients, depending on capacity, may impact on throughput of patients, waiting times, waiting lists, outcomes of surgery, costs, and equity of access to surgery. Inevitably outsourcing simpler patients to the independent sector will leave more complex patients being treated by NHS Trusts, which could have a detrimental impact on their service. An appropriate balance would need to be achieved to maximise the benefits for patients, and research is needed to understand what that balance is. Additionally, we need to understand whether this type of scheduling and outsourcing is acceptable to people waiting for hip and knee surgery as well as clinicians. These issues of optimising limited elective resources are in even sharper focus due to the backlog in waiting lists caused by the COVID-19 pandemic.

Unanswered questions and future research

We need to understand how the scheduling and possible outsourcing of elective surgery for different types of patients, depending on capacity, may impact on throughput of patients, waiting times, waiting lists, outcomes of surgery, costs, and equity of access to surgery. Inevitably outsourcing simpler patients to the independent sector will leave more complex patients being treated by NHS Trusts, which could have a detrimental impact on their service. An appropriate balance would need to be achieved to maximise the benefits for patients, and research is needed to understand what that balance is. Additionally, we need to understand whether this type of scheduling and outsourcing is acceptable to people waiting for hip and knee surgery as well as clinicians.

For peer review only - http://bmjopen.bmj.com/site/about/guidelines.xhtml

Conclusions

Declining elective capacity and seasonality has a marked effect on the provision of joint replacement, despite efficiency improvements in hospital treatment. The Trust has outsourced less complex patients to independent providers, and/or treated them during winter when capacity is most limited. There is a need to explore whether these are strategies that could be used explicitly to maximise the use of limited elective capacity, provide benefit to patients, and value for money for taxpayers.

For peer review only

Author Contributions

This publication is the work of the authors, who serve as guarantors for the contents of this paper. TJ contributed to study design, data cleaning, data analysis, interpretation of results and writing the manuscript. MTR contributed to study conceptualisation, supervision, interpretation of results and reviewing the manuscript. TK contributed to data curation, supervision, interpretation of results and reviewing the manuscript. AE contributed to data curation, interpretation of results and reviewing the manuscript. CP and EE contributed to interpretation of results and reviewing the manuscript. AB contributed to study conceptualisation, supervision, interpretation of results and reviewing the manuscript. AJ contributed to study conceptualisation and design, supervision, and writing the manuscript. TJ had full access to the data in the study and takes responsibility for the integrity of the data and the accuracy of the data analysis.

Acknowledgements

The study reported here would not have been possible without information provided by the NHS Trust, and the use of their hospital admissions data via the NIHR ARC West Partnership Agreement.

For peer review only - http://bmjopen.bmj.com/site/about/guidelines.xhtml

Funding

This research was funded by Health Data Research UK (HDR UK) Better Care South-West Partnership. EE, TJ, and MTR's time was supported by the National Institute for Health Research Applied Research Collaboration West (NIHR ARC West). AJ and CP were supported by the NIHR Biomedical Research Centre at University Hospitals Bristol and Weston NHS Foundation Trust and the University of Bristol. The views expressed in this article are those of the author(s) and not necessarily those of the NHS, the NIHR, the Department of Health and Social Care or HDR UK.

Ethical Approval

We were provided with pseudonymised hospital admissions data from the NHS Trust under the NIHR ARC West Partnership Agreement. The project received ethical approval from the University of Bristol Faculty of Health Sciences ethical review board on 3rd November 2020 (ref# 109024).

We were provided with routinely-collected Hospital Episode Statistics data under licence from NHS Digital (DARS-NIC-17875-X7K1V). The licence allows us to use the information under Section 261 of the Health and Social Care Act 2012, 2(b)(ii): "after taking into account the public interest as well as the interests of the relevant person, considers that it is appropriate for the information to be disseminated".

Data Sharing

The data from the NHS Trust was obtained under the NIHR ARC West Partnership Agreement. The agreement precludes us from sharing the raw data but it can be published and shared once aggregated to a non-identifiable level.

For peer review only - http://bmjopen.bmj.com/site/about/guidelines.xhtml

The data from Hospital Episode Statistics (HES) was obtained under licence (DARS-NIC-17875-X7K1V) from NHS Digital (previously the Health and Social Care Information Centre); *Copyright © 2022, re-used with the permission of The Health & Social Care Information Centre. All rights reserved*. The data are provided by patients and collected by the NHS as part of their care and support. HES data can be accessed via NHS Digital: https://digital.nhs.uk/services/data-access-request-service-dars

Transparency

The manuscript's guarantor (TJ) affirms that the manuscript is an honest, accurate, and transparent account of the study being reported; that no important aspects of the study have been omitted; and that any discrepancies from the study as originally planned have been explained.

Competing Interests

All authors have completed the ICMJE uniform disclosure form at www.icmje.org/coi_disclosure.pdf and declare: TJ, EE, and TR had financial support from NIHR ARC West for the submitted work; AJ has had financial support in the previous three years through institutional grants from NIHR, HDR UK, Versus Arthritis, Healthcare Quality Improvement Partnership (HQIP), Royal College of Physicians (RCP), and Health Foundation, had unpaid committee or leadership roles relating to musculoskeletal conditions for NIHR, Nuffield Foundation, Warwick CTU, and Versus Arthritis, and a paid expert panel role for Nuffield Foundation Oliver Bird Fund; no other financial relationships with any organisations that might have an interest in the submitted work in the previous three years; no other relationships or activities that could appear to have influenced the submitted work.

REFERENCES

1. Carr AJ, Robertsson O, Graves S, et al. Knee replacement. *Lancet* 2012;379(9823):1331-40. doi: 10.1016/S0140-6736(11)60752-6

For peer review only - http://bmjopen.bmj.com/site/about/guidelines.xhtml

2. Pivec R, Johnson AJ, Mears SC, et al. Hip arthroplasty. *Lancet* 2012;380(9855):1768-77. doi: 10.1016/S0140-6736(12)60607-2

3. Price AJ, Alvand A, Troelsen A, et al. Hip and knee replacement 2 Knee replacement. *Lancet* 2018;392(10158):1672-82. doi: Doi 10.1016/S0140-6736(18)32344-4

4. Ferguson RJ, Palmer AJ, Taylor A, et al. Hip replacement. *Lancet* 2018;392(10158):1662-71. doi: 10.1016/S0140-6736(18)31777-X [published Online First: 2018/11/30]

5. Culliford DJ, Maskell J, Beard DJ, et al. Temporal trends in hip and knee replacement in the United Kingdom 1991 TO 2006. *J Bone Joint Surg Br* 2010;92b(1):130-35. doi: 10.1302/0301-620x.92b1.22654

6. Burn E, Edwards CJ, Murray DW, et al. Trends and determinants of length of stay and hospital reimbursement following knee and hip replacement: evidence from linked primary care and NHS hospital records from 1997 to 2014. *Bmj Open* 2018;8(1) doi: 10.1136/bmjopen-2017-019146

7. Kehlet H. Fast-track hip and knee arthroplasty. *Lancet* 2013;381(9878):1600-02. doi: Doi 10.1016/S0140-6736(13)61003-X

8. Briggs T. A national review of adult elective orthopaedic services in England 2015. Available from: https://gettingitrightfirsttime.co.uk/wp-content/uploads/2017/06/GIRFT-National-Report-Mar15-Web.pdf.

9. Ward D, Wenzel L. A new trend in elective hip surgery 2019. Available from: https://www.kingsfund.org.uk/blog/2019/06/new-trend-elective-hip-surgery.

10. Fisher E, Merry L. Winter 2017/18: the worst ever for the NHS? 2018. Available from: https://www.nuffieldtrust.org.uk/news-item/winter-2017-18-the-worst-ever-for-the-nhs.

11. BMJ. Covid-19: “Life on hold” for NHS patients needing musculoskeletal care 2021. Available from: https://doi.org/10.1136/bmj.n1616.

12. Bernal JL, Cummins S, Gasparrini A. Interrupted time series regression for the evaluation of public health interventions: a tutorial. *Int J Epidemiol* 2017;46(1):348-55. doi: 10.1093/ije/dyw098

13. Kontopantelis E, Doran T, Springate DA, et al. Regression based quasi-experimental approach when randomisation is not an option: interrupted time series analysis. *Bmj-Brit Med J* 2015;350 doi: 10.1136/bmj.h2750

14. Leatherdale ST. Natural experiment methodology for research: a review of how different methods can support real-world research. *Int J Soc Res Method* 2019;22(1):19-35. doi: 10.1080/13645579.2018.1488449

15. Benchimol EI, Smeeth L, Guttmann A, et al. The REporting of studies Conducted using Observational Routinely-collected health Data (RECORD) Statement. *Plos Med* 2015;12(10) doi: 10.1371/journal.pmed.1001885

16. Herbert A, Wijlaars L, Zylbersztejn A, et al. Data Resource Profile: Hospital Episode Statistics Admitted Patient Care (HES APC). *Int J Epidemiol* 2017;46(4):1093-+. doi: 10.1093/ije/dyx015

17. Quan H, Sundararajan V, Halfon P, et al. Coding algorithms for defining comorbidities in ICD-9-CM and ICD-10 administrative data. *Med Care* 2005;43(11):1130-9. doi: 10.1097/01.mlr.0000182534.19832.83 [published Online First: 2005/10/15]

18. Office for National Statistics. LSOA (2011) to Clinical Commissioning Groups to Sustainability and Transformation Partnerships (April 2021) Lookup in England 2021. Available from: https://geoportal.statistics.gov.uk/datasets/ons::lsoa-2011-to-clinical-commissioning-groups-to-sustainability-and-transformation-partnerships-april-2021-lookup-in-england/explore.

19. Bhaskaran K, Gasparrini A, Hajat S, et al. Time series regression studies in environmental epidemiology. *Int J Epidemiol* 2013;42(4):1187-95. doi: 10.1093/ije/dyt092

20. Bottomley C, Scott JAG, Isham V. Analysing Interrupted Time Series with a Control. *Epidemiologic Methods* 2019;8(1) doi: 10.1515/em-2018-0010

For peer review only

For peer review only - http://bmjopen.bmj.com/site/about/guidelines.xhtml

21. Linden A. Combining synthetic controls and interrupted time series analysis to improve causal inference in program evaluation. *J Eval Clin Pract* 2018;24(2):447-53. doi: 10.1111/jep.12882
22. Dorward J, Khubone T, Gate K, et al. The impact of the COVID-19 lockdown on HIV care in 65 South African primary care clinics: an interrupted time series analysis. *Lancet Hiv* 2021;8(3):e158-e65. doi: 10.1016/S2352-3018(20)30359-3
23. Sutaria S, Kirkwood G, Pollock AM. An ecological study of NHS funded elective hip arthroplasties in England from 2003/04 to 2012/13. *J Roy Soc Med* 2019;112(7):292-303. doi: 10.1177/0141076819851701
24. The Independent. NHS privatisation fears after figures show third of knee replacements done by private sector 2018. Available from: https://www.independent.co.uk/news/uk/home-news/nhs-knee-hip-replacements-private-sector-a8549006.html.
25. Dench J. Private hospital overtakes NHS for hip and knee replacement 2021. Available from: https://londonnewstime.com/private-hospital-overtakes-nhs-for-hip-and-knee-replacement/479438/.
26. Herrod PJJ, Adiamah A, Boyd-Carson H, et al. Winter cancellations of elective surgical procedures in the UK: a questionnaire survey of patients on the economic and psychological impact. *Bmj Open* 2019;9(9) doi: 10.1136/bmjopen-2018-028753

For peer review only

For peer review only - http://bmjopen.bmj.com/site/about/guidelines.xhtml

FIGURE CAPTIONS

Figure 1. Elective hip (left panel) and knee (right panel) replacement admissions at the Trust
Note: grey area shows the winter 2017 cancellations and is excluded from the analysis

Figure 2. Average age on admission for hip (left panel) and knee (right panel) replacements at the Trust
Note: grey area shows the winter 2017 cancellations and is excluded from the analysis

Figure 3. Proportion of people having hip (left panel) and knee (right panel) replacements with 2+ Charlson comorbidities recorded
Note: grey area shows the winter 2017 cancellations and is excluded from the analysis

Figure 4. Ratio of public to private provision of elective hip (left panel) and knee (right panel) replacements for NHS patients in the Trust CCG
Note: grey area shows the winter 2017 cancellations and is excluded from the analysis

Figure 5. Bed occupancy for hip (left panel) and knee (right panel) replacements at the Trust
Note: grey area shows the winter 2017 cancellations and is excluded from the analysis

For peer review only

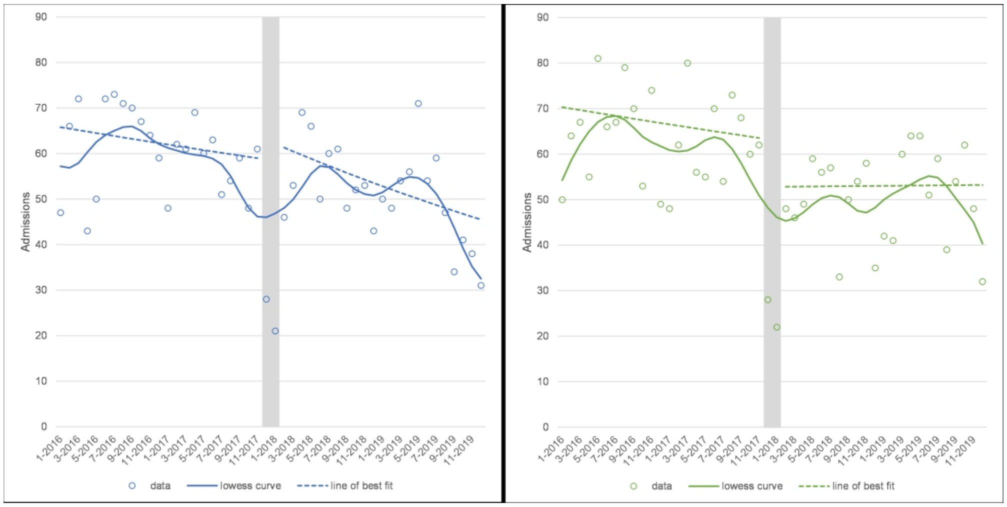

Figure 1. Elective hip (left panel) and knee (right panel) replacement admissions at the Trust
Note: grey area shows the winter 2017 cancellations and is excluded from the analysis

301x150mm (300 x 300 DPI)

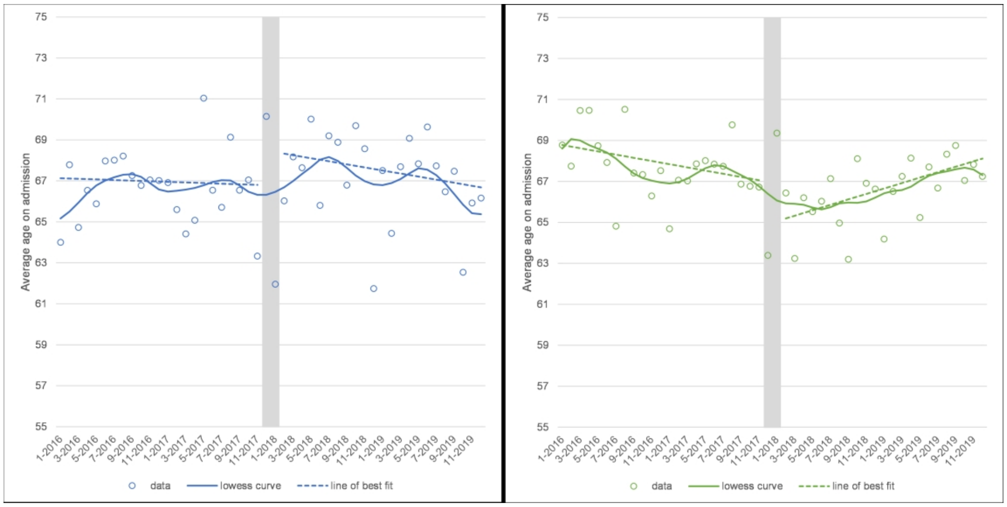

Figure 2. Average age on admission for hip (left panel) and knee (right panel) replacements at the Trust
Note: grey area shows the winter 2017 cancellations and is excluded from the analysis

301x150mm (300 x 300 DPI)

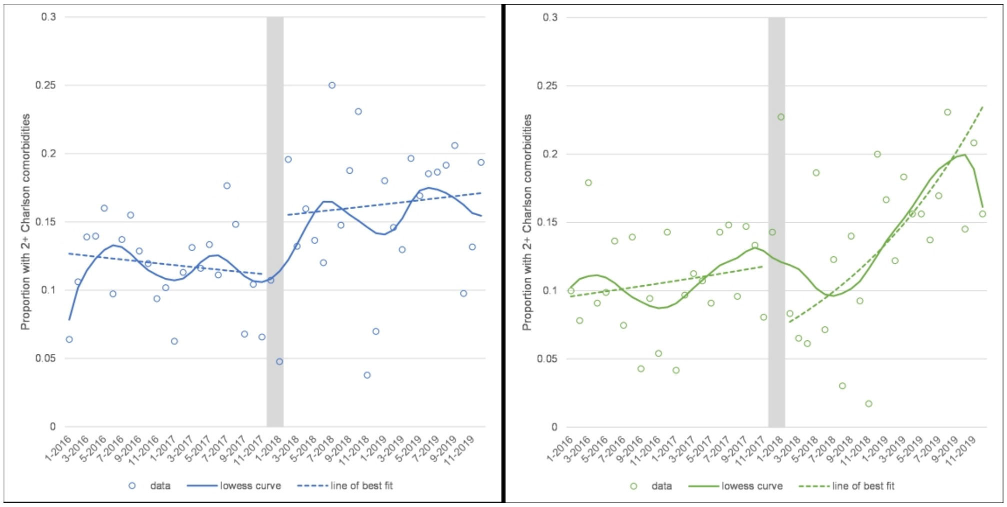

Figure 3. Proportion of people having hip (left panel) and knee (right panel) replacements with 2+ Charlson comorbidities recorded
Note: grey area shows the winter 2017 cancellations and is excluded from the analysis

301x150mm (300 x 300 DPI)

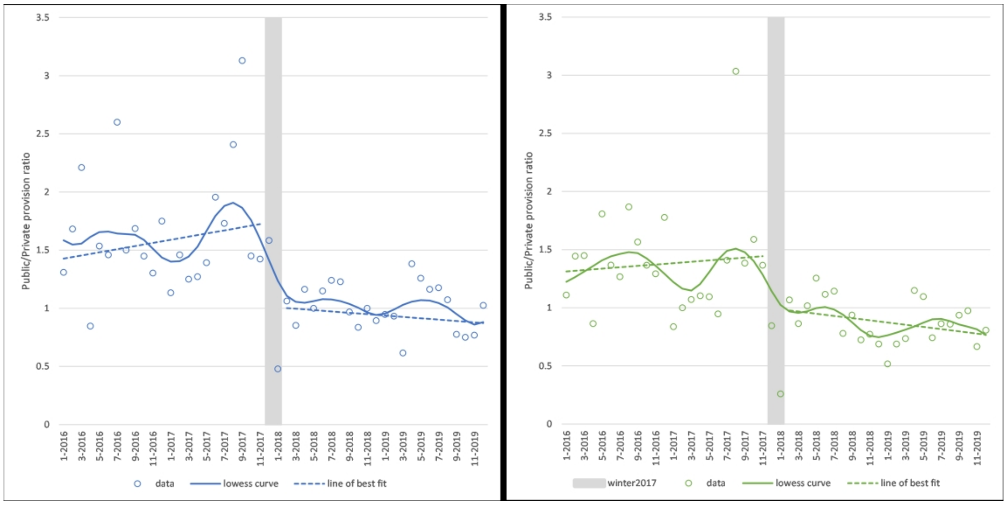

Figure 4. Ratio of public to private provision of elective hip (left panel) and knee (right panel) replacements for NHS patients in the Trust CCG
Note: grey area shows the winter 2017 cancellations and is excluded from the analysis

301x150mm (300 x 300 DPI)

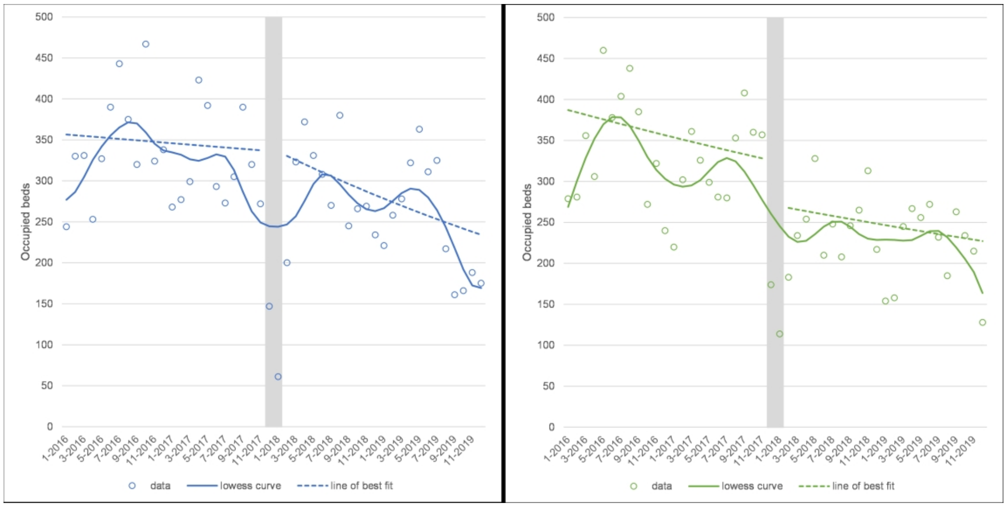

Figure 5. Bed occupancy for hip (left panel) and knee (right panel) replacements at the Trust
Note: grey area shows the winter 2017 cancellations and is excluded from the analysis

301x150mm (300 x 300 DPI)

Supplementary Figure F1. Ratio of elective to emergency hospital admissions for any reason at the Trust

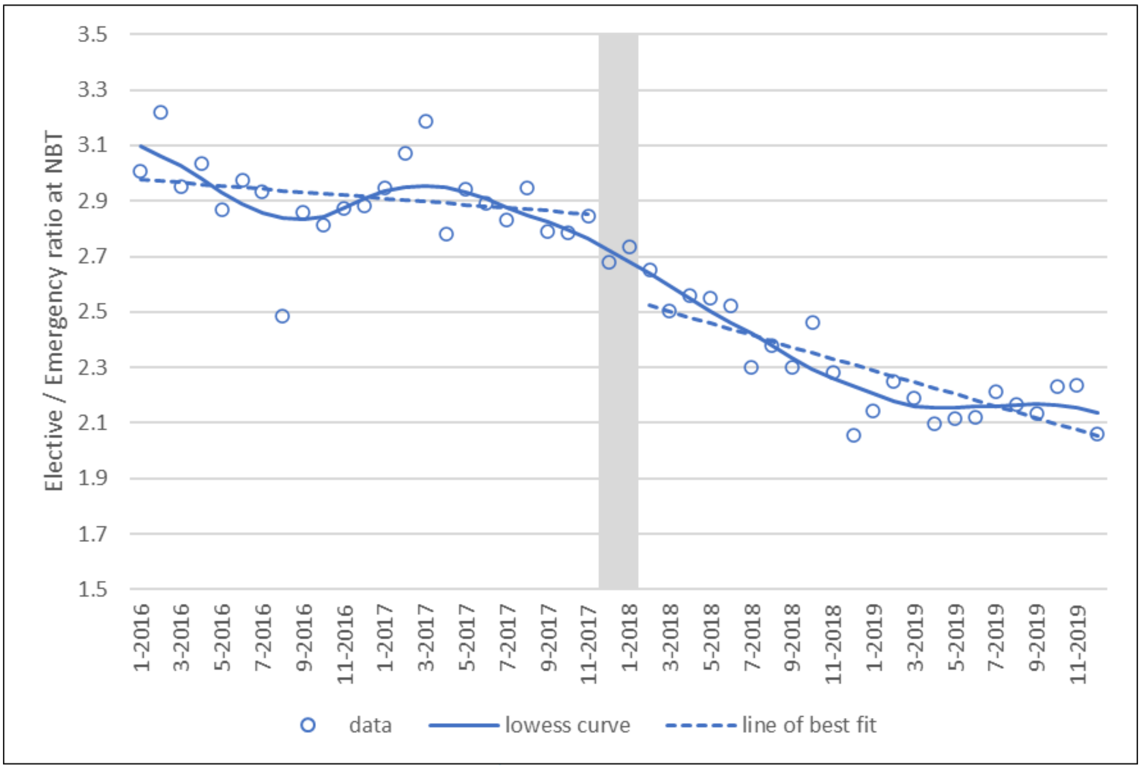

Note: grey area shows the winter 2017 cancellations and is excluded from the analysis

review only

Supplementary Figure F2. Average length of stay for primary hip (left) and knee (right) replacement operations at NBT

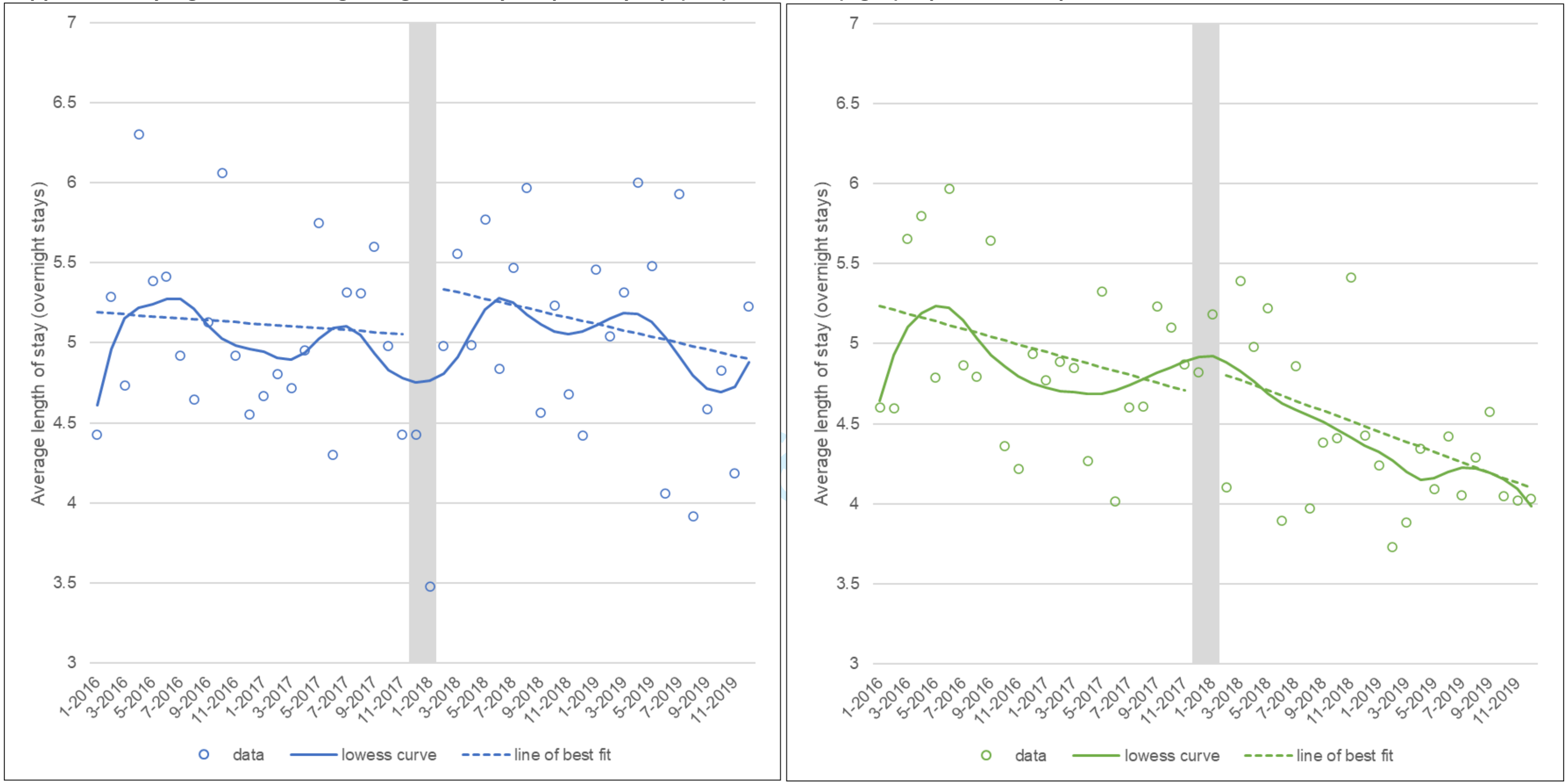

Note: grey area shows the winter 2017 cancellations and is excluded from the analysis

For peer review only

For peer review only - http://bmjopen.bmj.com/site/about/guidelines.xhtml

Supplementary Table T1. OPCS-4 codes used to identify primary hip and knee replacement operations

Category	Code	Description	Notes
Primary Total Hip Replacement	W37.1	Primary total prosthetic replacement of hip joint using cement	
	W37.8	Other specified total prosthetic replacement of hip joint using cement	
	W37.9	Unspecified total prosthetic replacement of hip joint using cement	
	W38.1	Primary total prosthetic replacement of hip joint not using cement	
	W38.8	Other specified total prosthetic replacement of hip joint not using cement	
	W38.9	Unspecified total prosthetic replacement of hip joint not using cement	
	W39.1	Primary total prosthetic replacement of hip joint NEC	
	W39.8	Other specified other total prosthetic replacement of hip joint	
	W39.9	Unspecified other total prosthetic replacement of hip joint	
	W43.1	Primary total prosthetic replacement of other joint using cement NEC	
	W43.8	Other specified total prosthetic replacement of other joint using cement NEC	
	W43.9	Unspecified total prosthetic replacement of other joint using cement NEC	
	W44.1	Primary total prosthetic replacement of other joint not using cement NEC	
	W44.8	Other specified total prosthetic replacement of other joint not using cement NEC	
	W44.9	Unspecified total prosthetic replacement of other joint not using cement NEC	
	W45.1	Other primary total prosthetic replacement of other joint NEC	
	W45.8	Other specified total prosthetic replacement of other joint NEC	
	W45.9	Unspecified total prosthetic replacement of other joint NEC	
	W52.1	Primary prosthetic replacement of articulation of bone using cement NEC	

For peer review only - http://bmjopen.bmj.com/site/about/guidelines.xhtml

W52.8	Other specified prosthetic replacement of articulation of bone using cement NEC
W52.9	Unspecified prosthetic replacement of articulation of bone using cement NEC
W53.1	Primary prosthetic replacement of articulation of bone not using cement NEC
W53.8	Other specified prosthetic replacement of articulation of bone not using cement NEC
W53.9	Unspecified prosthetic replacement of articulation of bone not using cement NEC
W54.1	Primary prosthetic replacement of articulation of bone NEC
W54.8	Other specified prosthetic replacement of articulation of bone NEC
W54.9	Unspecified prosthetic replacement of articulation of bone NEC
W93.1	Primary hybrid prosthetic replacement of hip joint using cemented acetabular component
W93.8	Other specified hybrid prosthetic replacement of hip joint using cemented acetabular component
W93.9	Unspecified hybrid prosthetic replacement of hip joint using cemented acetabular component
W94.1	Primary hybrid prosthetic replacement of hip joint using cemented femoral component
W94.8	Other specified hybrid prosthetic replacement of hip joint using cemented femoral component
W94.9	Unspecified hybrid prosthetic replacement of hip joint using cemented femoral component
W95.1	Primary hybrid prosthetic replacement of hip joint using cement NEC
W95.8	Other specified hybrid prosthetic replacement of hip joint using cement
W95.9	Unspecified hybrid prosthetic replacement of hip joint using cement

For peer review only

For peer review only - http://bmjopen.bmj.com/site/about/guidelines.xhtml

Primary Total Knee Replacement	W40.1	Primary total prosthetic replacement of knee joint using cement	
	W40.8	Other specified total prosthetic replacement of knee joint using cement	
	W40.9	Unspecified total prosthetic replacement of knee joint using cement	
	W41.1	Primary total prosthetic replacement of knee joint not using cement	
	W41.8	Other specified total prosthetic replacement of knee joint not using cement	
	W41.9	Unspecified total prosthetic replacement of knee joint not using cement	
	W42.1	Primary total prosthetic replacement of knee joint NEC	
	W42.8	Other specified other total prosthetic replacement of knee joint	
	W42.9	Unspecified other total prosthetic replacement of knee joint	
	O18.1	Primary hybrid prosthetic replacement of knee joint using cement	
	O18.8	Other specified hybrid prosthetic replacement of knee joint using cement	
	O18.9	Unspecified hybrid prosthetic replacement of knee joint using cement	
Resurfacing / Reconstruction	W58.1	Primary resurfacing arthroplasty of joint	Require combination with site + combination codes to ID
	W58.8	Other specified reconstruction of joint	Require combination with site + combination codes to ID
	W58.9	Unspecified other reconstruction of joint	Require combination with site + combination codes to ID
Primary unicondylar / unicompartmental knee operations	W52.1	Primary prosthetic replacement of articulation of bone using cement NEC	Require combination with site + combination codes to ID
	W52.8	Other specified prosthetic replacement of articulation of other bone using cement	Require combination with site + combination codes to ID
	W52.9	Unspecified prosthetic replacement of articulation of other bone using cement	Require combination with site + combination codes to ID
	W53.1	Primary prosthetic replacement of articulation of bone not using cement NEC	Require combination with site + combination codes to ID

For peer review only - http://bmjopen.bmj.com/site/about/guidelines.xhtml

For peer review only

W53.9	Unspecified prosthetic replacement of articulation of other bone not using cement	Require combination with site + combination codes to ID
W54.0	Conversion from previous prosthetic replacement of articulation of bone NEC	Require combination with site + combination codes to ID
W54.1	Primary prosthetic replacement of articulation of bone NEC	Require combination with site + combination codes to ID
W54.8	Other specified other prosthetic replacement of articulation of other bone	Require combination with site + combination codes to ID
W54.9	Unspecified other prosthetic replacement of articulation of other bone	Require combination with site + combination codes to ID

For peer review only

For peer review only - http://bmjopen.bmj.com/site/about/guidelines.xhtml

Supplementary Table T2. Interrupted time series model results with maximum auto-correlation lag 2

	pre-trend		level change		trend change		spring		summer		autumn	
	estimate (95% CI)	p	estimate (95% CI)	p	estimate (95% CI)	p	estimate (95% CI)	p	estimate (95% CI)	p	estimate (95% CI)	p
Hip Admissions	0.995 (0.986,1.004)	0.300	1.055 (0.912,1.221)	0.469	0.991 (0.977,1.006)	0.239	1.19 (1.034,1.37)	0.015	1.207 (1.094,1.332)	0.000	1.101 (0.982,1.234)	0.099
Hip Average Age	-0.015 (-0.102,0.072)	0.737	1.571 (-0.1,3.242)	0.065	-0.06 (-0.175,0.055)	0.307	1.52 (-0.07,3.111)	0.061	2.09 (0.811,3.368)	0.001	1.031 (-0.598,2.66)	0.215
Hip Prop Women	0.999 (0.995,1.003)	0.582	0.966 (0.861,1.083)	0.549	1.007 (0.999,1.016)	0.089	1.023 (0.941,1.111)	0.596	1.088 (1.001,1.183)	0.048	0.981 (0.888,1.083)	0.700
Hip Prop 2+ Charlson	0.994 (0.982,1.007)	0.380	1.411 (1.064,1.873)	0.017	1.01 (0.994,1.027)	0.220	1.195 (0.982,1.455)	0.075	1.306 (1.096,1.557)	0.003	1.003 (0.788,1.276)	0.982
Hip Prop High Deprivation	1.003 (0.993,1.013)	0.587	1.027 (0.869,1.214)	0.754	1.004 (0.986,1.022)	0.660	0.937 (0.815,1.076)	0.358	0.877 (0.752,1.022)	0.093	0.996 (0.84,1.18)	0.963
Hip LoS	-0.006 (-0.034,0.021)	0.660	0.312 (-0.193,0.818)	0.225	-0.014 (-0.047,0.02)	0.425	0.502 (0.214,0.79)	0.001	0.135 (-0.194,0.463)	0.422	0.1 (-0.288,0.488)	0.613
Hip LoS Age 16-59	-0.003 (-0.028,0.021)	0.788	0.379 (-0.094,0.851)	0.116	-0.026 (-0.065,0.013)	0.190	0.223 (-0.156,0.602)	0.249	0.384 (0.065,0.703)	0.018	0.219 (-0.183,0.622)	0.285
Hip LoS Age 60-69	-0.004 (-0.034,0.026)	0.818	0.013 (-0.807,0.832)	0.976	0 (-0.053,0.052)	0.988	0.107 (-0.44,0.654)	0.702	0.158 (-0.468,0.783)	0.621	0.162 (-0.487,0.811)	0.625
Hip LoS Age 70-79	0.006 (-0.058,0.069)	0.862	-0.753 (-2.003,0.497)	0.238	0.007 (-0.06,0.073)	0.847	-0.433 (-1.416,0.549)	0.387	-0.842 (-1.93,0.246)	0.129	-0.865 (-1.991,0.262)	0.132
Hip LoS Age 80+	-0.068 (-0.171,0.036)	0.199	2.109 (0.683,3.535)	0.004	-0.002 (-0.128,0.123)	0.971	1.506 (0.515,2.497)	0.003	0.222 (-0.773,1.217)	0.662	1.003 (-0.229,2.235)	0.111
Hip LoS Men	-0.007 (-0.055,0.041)	0.776	0.347 (-0.583,1.278)	0.464	-0.02 (-0.083,0.043)	0.536	0.293 (-0.126,0.712)	0.170	0.289 (-0.196,0.774)	0.243	0.458 (-0.111,1.026)	0.114
Hip LoS Women	-0.004 (-0.041,0.033)	0.842	0.235 (-0.308,0.778)	0.396	-0.014 (-0.055,0.027)	0.505	0.693 (0.309,1.077)	0.000	0.009 (-0.428,0.446)	0.966	-0.203 (-0.728,0.323)	0.449
Hip LoS Charlson 0	-0.011 (-0.042,0.019)	0.469	0.297 (-0.288,0.882)	0.319	-0.005 (-0.042,0.031)	0.782	0.912 (0.549,1.276)	0.000	0.178 (-0.148,0.503)	0.285	0.222 (-0.065,0.509)	0.129
Hip LoS Charlson 1	0.034 (-0.012,0.08)	0.146	0.238 (-0.709,1.184)	0.623	-0.077 (-0.145,-0.01)	0.024	0.191 (-0.418,0.801)	0.538	0.312 (-0.405,1.029)	0.393	0.014 (-0.578,0.606)	0.963
Hip LoS Charlson 2+	-0.123 (-0.259,0.013)	0.077	0.172 (-2.224,2.569)	0.888	0.116 (-0.016,0.249)	0.085	-0.789 (-2.141,0.563)	0.253	-0.783 (-2.087,0.521)	0.239	0.188 (-2.051,2.427)	0.869
Hip LoS Dep 1	0.004 (-0.032,0.04)	0.829	0.553 (-0.391,1.496)	0.251	-0.017 (-0.068,0.035)	0.529	0.784 (0.107,1.462)	0.023	0.42 (-0.024,0.863)	0.064	0.299 (-0.358,0.956)	0.373
Hip LoS Dep 2	-0.038 (-0.083,0.006)	0.092	0.822 (-0.392,2.035)	0.184	-0.015 (-0.086,0.055)	0.670	1.509 (0.759,2.258)	0.000	0.66 (-0.246,1.567)	0.154	0.589 (-0.417,1.594)	0.251
Hip LoS Dep 3	-0.034 (-0.107,0.038)	0.353	0.617 (-0.897,2.131)	0.424	0.01 (-0.076,0.095)	0.827	-0.216 (-1.224,0.792)	0.675	-0.424 (-1.378,0.53)	0.384	-0.701 (-1.536,0.135)	0.100
Hip LoS Dep 4	0.081 (-0.005,0.166)	0.064	-0.473 (-2.181,1.236)	0.588	-0.112 (-0.215,-0.009)	0.034	-0.374 (-1.65,0.903)	0.566	-1.204 (-2.351,-0.057)	0.040	-0.491 (-1.918,0.935)	0.500
Hip LoS Dep 5	0.015 (-0.086,0.116)	0.773	-0.72 (-2.059,0.62)	0.292	0 (-0.098,0.099)	0.999	0.621 (-0.084,1.327)	0.084	0.689 (-0.31,1.689)	0.176	0.816 (-0.784,2.416)	0.317
Hip Bed Occ	0.997 (0.987,1.008)	0.643	1 (0.844,1.186)	0.997	0.987 (0.97,1.005)	0.149	1.291 (1.11,1.501)	0.001	1.283 (1.125,1.463)	0.000	1.146 (0.988,1.328)	0.071
Hip Public Private	0.013 (-0.015,0.041)	0.377	-0.741 (-1.237,-0.245)	0.003	-0.019 (-0.05,0.011)	0.218	-0.008 (-0.212,0.196)	0.939	0.308 (0.154,0.463)	0.000	0.038 (-0.166,0.241)	0.718
Knee Admissions	0.995 (0.99,1.001)	0.106	0.843 (0.728,0.976)	0.022	1.005 (0.996,1.014)	0.256	1.308 (1.157,1.479)	0.000	1.26 (1.138,1.396)	0.000	1.286 (1.164,1.42)	0.000
Knee Average Age	-0.078 (-0.157,0.001)	0.054	-1.632 (-2.988,-0.276)	0.018	0.211 (0.117,0.305)	0.000	0.926 (-0.112,1.965)	0.080	0.953 (-0.021,1.927)	0.055	0.354 (-0.531,1.24)	0.433
Knee Prop Women	1.004 (0.998,1.01)	0.150	0.96 (0.85,1.084)	0.513	0.994 (0.986,1.003)	0.193	1.037 (0.963,1.117)	0.336	1.017 (0.924,1.118)	0.735	1.036 (0.958,1.12)	0.375
Knee Prop 2+ Charlson	1.009 (0.993,1.026)	0.249	0.638 (0.455,0.894)	0.009	1.042 (1.017,1.067)	0.001	1.156 (0.911,1.467)	0.234	1.074 (0.849,1.359)	0.551	0.909 (0.628,1.315)	0.612
Knee Prop High Deprivation	1.005 (0.997,1.013)	0.189	0.968 (0.786,1.191)	0.758	0.986 (0.974,0.998)	0.021	1.224 (1.077,1.39)	0.002	1.075 (0.942,1.227)	0.282	1.031 (0.902,1.178)	0.656
Knee LoS	-0.024 (-0.049,0.001)	0.058	0.176 (-0.279,0.63)	0.449	-0.008 (-0.036,0.02)	0.566	0.422 (0.073,0.771)	0.018	0.15 (-0.166,0.467)	0.352	0.396 (0.015,0.777)	0.042

For peer review only - http://bmjopen.bmj.com/site/about/guidelines.xhtml

Knee LoS Age 16-59	-0.016 (-0.037,0.006)	0.151	0.403 (-0.045,0.852)	0.078	-0.028 (-0.06,0.005)	0.096	0.484 (0.13,0.839)	0.007	0.422 (-0.021,0.866)	0.062	0.475 (0.19,0.76)	0.001
Knee LoS Age 16-59	0.007 (-0.028,0.043)	0.684	-0.295 (-1.015,0.426)	0.423	-0.026 (-0.072,0.02)	0.262	0.069 (-0.46,0.599)	0.797	-0.194 (-0.687,0.3)	0.441	-0.096 (-0.725,0.532)	0.764
Knee LoS Age 70-79	-0.009 (-0.057,0.038)	0.705	0.77 (0.002,1.538)	0.049	-0.054 (-0.11,0.003)	0.061	-0.086 (-0.715,0.542)	0.787	-0.552 (-1.163,0.059)	0.077	0.367 (-0.352,1.085)	0.317
Knee LoS Age 80+	-0.051 (-0.118,0.015)	0.131	-0.562 (-2.298,1.173)	0.525	0.028 (-0.081,0.137)	0.612	1.521 (0.03,3.011)	0.046	1.37 (0.169,2.57)	0.025	1.361 (0.496,2.227)	0.002
Knee LoS Men	-0.007 (-0.031,0.018)	0.581	0.096 (-0.405,0.597)	0.708	-0.033 (-0.068,0.002)	0.064	0.082 (-0.367,0.531)	0.720	0.157 (-0.262,0.575)	0.464	0.205 (-0.221,0.631)	0.346
Knee LoS Women	-0.042 (-0.084,0)	0.052	0.252 (-0.461,0.966)	0.488	0.017 (-0.029,0.063)	0.478	0.635 (0.048,1.223)	0.034	0.15 (-0.397,0.698)	0.590	0.49 (-0.17,1.151)	0.146
Knee LoS Charlson 0	-0.021 (-0.046,0.004)	0.095	0.067 (-0.362,0.495)	0.760	-0.011 (-0.045,0.024)	0.545	0.645 (0.334,0.956)	0.000	0.193 (-0.111,0.497)	0.213	0.415 (0.069,0.76)	0.019
Knee LoS Charlson 1	-0.03 (-0.074,0.015)	0.191	0.47 (-0.391,1.331)	0.284	-0.029 (-0.083,0.025)	0.298	0.455 (-0.043,0.953)	0.073	0.324 (-0.297,0.945)	0.307	1.058 (0.367,1.748)	0.003
Knee LoS Charlson 2+	-0.04 (-0.107,0.028)	0.247	0.407 (-2.28,3.095)	0.766	-0.022 (-0.196,0.151)	0.802	-0.627 (-1.698,0.443)	0.251	-0.949 (-2.342,0.444)	0.182	-1.433 (-2.229,-0.637)	0.000
Knee LoS Dep 1	-0.009 (-0.06,0.041)	0.712	-0.035 (-0.726,0.656)	0.920	-0.008 (-0.073,0.056)	0.800	0.257 (-0.47,0.985)	0.488	0.096 (-0.619,0.811)	0.793	-0.127 (-0.725,0.472)	0.678
Knee LoS Dep 2	-0.019 (-0.06,0.022)	0.358	0.199 (-0.699,1.097)	0.664	-0.043 (-0.098,0.012)	0.124	0.018 (-0.776,0.812)	0.965	-0.731 (-1.49,0.029)	0.059	0.05 (-0.822,0.922)	0.911
Knee LoS Dep 3	0.014 (-0.036,0.064)	0.586	-0.426 (-1.355,0.503)	0.369	-0.036 (-0.106,0.033)	0.302	0.976 (0.061,1.892)	0.036	0.909 (-0.034,1.852)	0.059	0.547 (-0.217,1.312)	0.160
Knee LoS Dep 4	-0.064 (-0.123,-0.004)	0.035	0.634 (-0.293,1.56)	0.180	0.033 (-0.039,0.105)	0.373	0.669 (-0.187,1.525)	0.126	0.617 (0.02,1.214)	0.043	0.852 (-0.208,1.912)	0.115
Knee LoS Dep 5	-0.035 (-0.09,0.021)	0.224	0.709 (-0.303,1.721)	0.170	0.015 (-0.046,0.076)	0.628	0.123 (-0.73,0.976)	0.778	-0.387 (-1.281,0.507)	0.397	1.104 (0.077,2.131)	0.035
Knee Bed Occ	0.993 (0.984,1.002)	0.103	0.834 (0.704,0.989)	0.037	1 (0.989,1.011)	0.993	1.42 (1.297,1.556)	0.000	1.373 (1.218,1.547)	0.000	1.465 (1.312,1.635)	0.000
Knee Public Private	0.006 (-0.02,0.031)	0.667	-0.476 (-1.026,0.074)	0.090	-0.015 (-0.04,0.009)	0.225	0.113 (-0.089,0.314)	0.274	0.276 (0.035,0.517)	0.025	0.131 (-0.071,0.332)	0.205
Elec Emerg Ratio	-0.005 (-0.013,0.002)	0.171	-0.322 (-0.446,-0.198)	0.000	-0.016 (-0.026,-0.005)	0.003	-0.008 (-0.12,0.103)	0.886	-0.053 (-0.182,0.077)	0.424	-0.028 (-0.151,0.096)	0.661

For peer review only

For peer review only - http://bmjopen.bmj.com/site/about/guidelines.xhtml

Supplementary Table T3. Interrupted time series model results with maximum auto-correlation lag 0

	pre-trend		level change		trend change		spring		summer		autumn	
	estimate (95% CI)	p	estimate (95% CI)	p	estimate (95% CI)	p	estimate (95% CI)	p	estimate (95% CI)	p	estimate (95% CI)	p
Hip Admissions	0.995 (0.985,1.005)	0.323	1.055 (0.902,1.235)	0.503	0.991 (0.978,1.005)	0.220	1.19 (1.036,1.367)	0.014	1.207 (1.088,1.339)	0.000	1.101 (0.983,1.234)	0.098
Hip Average Age	-0.015 (-0.134,0.104)	0.806	1.571 (-0.863,4.004)	0.206	-0.06 (-0.208,0.088)	0.427	1.52 (-0.079,3.119)	0.062	2.09 (0.685,3.494)	0.004	1.031 (-0.505,2.567)	0.188
Hip Prop Women	0.999 (0.993,1.004)	0.674	0.966 (0.826,1.13)	0.662	1.007 (0.997,1.017)	0.158	1.023 (0.937,1.116)	0.617	1.088 (0.995,1.189)	0.064	0.981 (0.878,1.096)	0.730
Hip Prop 2+ Charlson	0.994 (0.98,1.009)	0.455	1.411 (0.969,2.055)	0.072	1.01 (0.989,1.031)	0.344	1.195 (0.971,1.471)	0.092	1.306 (1.043,1.637)	0.020	1.003 (0.734,1.37)	0.986
Hip Prop High Deprivation	1.003 (0.991,1.014)	0.631	1.027 (0.846,1.247)	0.787	1.004 (0.983,1.025)	0.704	0.937 (0.819,1.072)	0.342	0.877 (0.746,1.03)	0.110	0.996 (0.831,1.193)	0.965
Hip LoS	-0.006 (-0.038,0.026)	0.704	0.312 (-0.262,0.887)	0.287	-0.014 (-0.058,0.03)	0.542	0.502 (0.132,0.872)	0.008	0.135 (-0.292,0.562)	0.537	0.1 (-0.287,0.487)	0.612
Hip LoS Age 16-59	-0.003 (-0.033,0.026)	0.821	0.379 (-0.165,0.922)	0.172	-0.026 (-0.067,0.015)	0.210	0.223 (-0.17,0.617)	0.266	0.384 (0.053,0.715)	0.023	0.219 (-0.168,0.607)	0.267
Hip LoS Age 60-69	-0.004 (-0.046,0.039)	0.871	0.013 (-1.026,1.052)	0.981	0 (-0.069,0.068)	0.990	0.107 (-0.592,0.805)	0.765	0.158 (-0.514,0.829)	0.645	0.162 (-0.588,0.912)	0.672
Hip LoS Age 70-79	0.006 (-0.067,0.078)	0.879	-0.753 (-2.031,0.525)	0.248	0.007 (-0.069,0.082)	0.865	-0.433 (-1.583,0.716)	0.460	-0.842 (-2.028,0.344)	0.164	-0.865 (-2.164,0.435)	0.192
Hip LoS Age 80+	-0.068 (-0.193,0.058)	0.291	2.109 (0.536,3.682)	0.009	-0.002 (-0.16,0.155)	0.977	1.506 (-0.007,3.018)	0.051	0.222 (-1.036,1.48)	0.729	1.003 (-0.375,2.38)	0.154
Hip LoS Men	-0.007 (-0.056,0.042)	0.783	0.347 (-0.58,1.275)	0.463	-0.02 (-0.084,0.044)	0.543	0.293 (-0.283,0.87)	0.319	0.289 (-0.314,0.892)	0.347	0.458 (-0.15,1.066)	0.140
Hip LoS Women	-0.004 (-0.048,0.041)	0.868	0.235 (-0.563,1.033)	0.564	-0.014 (-0.071,0.043)	0.634	0.693 (0.164,1.222)	0.010	0.009 (-0.543,0.562)	0.973	-0.203 (-0.727,0.322)	0.448
Hip LoS Charlson 0	-0.011 (-0.047,0.024)	0.532	0.297 (-0.428,1.023)	0.422	-0.005 (-0.049,0.039)	0.817	0.912 (0.522,1.302)	0.000	0.178 (-0.232,0.587)	0.396	0.222 (-0.135,0.579)	0.223
Hip LoS Charlson 1	0.034 (-0.023,0.091)	0.240	0.238 (-0.704,1.179)	0.621	-0.077 (-0.157,0.002)	0.056	0.191 (-0.562,0.945)	0.619	0.312 (-0.397,1.022)	0.388	0.014 (-0.544,0.572)	0.960
Hip LoS Charlson 2+	-0.123 (-0.266,0.02)	0.092	0.172 (-2.155,2.5)	0.885	0.116 (-0.04,0.273)	0.144	-0.789 (-2.218,0.64)	0.279	-0.783 (-2.108,0.541)	0.246	0.188 (-1.779,2.156)	0.851
Hip LoS Dep 1	0.004 (-0.039,0.047)	0.857	0.553 (-0.509,1.614)	0.307	-0.017 (-0.078,0.045)	0.599	0.784 (0.118,1.451)	0.021	0.42 (-0.084,0.923)	0.102	0.299 (-0.356,0.954)	0.371
Hip LoS Dep 2	-0.038 (-0.087,0.01)	0.124	0.822 (-0.528,2.172)	0.233	-0.015 (-0.103,0.072)	0.730	1.509 (0.797,2.22)	0.000	0.66 (-0.225,1.546)	0.144	0.589 (-0.26,1.437)	0.174
Hip LoS Dep 3	-0.034 (-0.096,0.027)	0.275	0.617 (-1.066,2.3)	0.472	0.01 (-0.09,0.109)	0.851	-0.216 (-1.343,0.912)	0.708	-0.424 (-1.475,0.627)	0.429	-0.701 (-1.667,0.266)	0.155
Hip LoS Dep 4	0.081 (-0.023,0.185)	0.129	-0.473 (-2.643,1.698)	0.670	-0.112 (-0.258,0.034)	0.134	-0.374 (-1.695,0.947)	0.579	-1.204 (-2.409,0.001)	0.050	-0.491 (-1.978,0.995)	0.517
Hip LoS Dep 5	0.015 (-0.112,0.142)	0.819	-0.72 (-2.66,1.221)	0.467	0 (-0.135,0.135)	0.999	0.621 (-0.412,1.655)	0.239	0.689 (-0.485,1.864)	0.250	0.816 (-0.891,2.524)	0.349
Hip Bed Occ	0.997 (0.987,1.009)	0.653	1 (0.824,1.215)	0.997	0.987 (0.972,1.002)	0.097	1.291 (1.125,1.48)	0.000	1.283 (1.129,1.458)	0.000	1.146 (0.984,1.334)	0.080
Hip Public Private	0.013 (-0.015,0.041)	0.377	-0.741 (-1.237,-0.245)	0.003	-0.019 (-0.05,0.011)	0.218	-0.008 (-0.212,0.196)	0.939	0.308 (0.154,0.463)	0.000	0.038 (-0.166,0.241)	0.718
Knee Admissions	0.995 (0.987,1.004)	0.274	0.843 (0.702,1.013)	0.068	1.005 (0.993,1.017)	0.422	1.308 (1.154,1.483)	0.000	1.26 (1.114,1.426)	0.000	1.286 (1.147,1.441)	0.000
Knee Average Age	-0.078 (-0.157,0.001)	0.053	-1.632 (-3.299,0.035)	0.055	0.211 (0.102,0.32)	0.000	0.926 (-0.129,1.981)	0.085	0.953 (-0.257,2.163)	0.123	0.354 (-0.777,1.485)	0.540
Knee Prop Women	1.004 (0.998,1.011)	0.182	0.96 (0.84,1.097)	0.551	0.994 (0.986,1.003)	0.184	1.037 (0.939,1.146)	0.474	1.017 (0.908,1.138)	0.776	1.036 (0.928,1.157)	0.529
Knee Prop 2+ Charlson	1.009 (0.988,1.031)	0.384	0.638 (0.392,1.037)	0.070	1.042 (1.008,1.077)	0.015	1.156 (0.891,1.501)	0.276	1.074 (0.829,1.393)	0.589	0.909 (0.636,1.299)	0.600
Knee Prop High Deprivation	1.005 (0.997,1.014)	0.237	0.968 (0.784,1.195)	0.761	0.986 (0.974,0.998)	0.023	1.224 (1.099,1.363)	0.000	1.075 (0.956,1.209)	0.225	1.031 (0.911,1.166)	0.631
Knee LoS	-0.024 (-0.052,0.004)	0.089	0.176 (-0.352,0.703)	0.515	-0.008 (-0.041,0.025)	0.625	0.422 (0.117,0.726)	0.007	0.15 (-0.174,0.475)	0.364	0.396 (0.027,0.765)	0.036

For peer review only

Knee LoS Age 16-59	-0.016 (-0.048,0.017)	0.352	0.403 (-0.286,1.093)	0.252	-0.028 (-0.074,0.019)	0.246	0.484 (0.111,0.857)	0.011	0.422 (-0.111,0.956)	0.121	0.475 (0.056,0.893)	0.026
Knee LoS Age 16-59	0.007 (-0.026,0.041)	0.664	-0.295 (-1.22,0.63)	0.532	-0.026 (-0.085,0.033)	0.384	0.069 (-0.465,0.604)	0.799	-0.194 (-0.754,0.366)	0.497	-0.096 (-0.673,0.48)	0.743
Knee LoS Age 70-79	-0.009 (-0.072,0.054)	0.775	0.77 (-0.267,1.807)	0.145	-0.054 (-0.128,0.02)	0.154	-0.086 (-0.782,0.609)	0.808	-0.552 (-1.267,0.163)	0.130	0.367 (-0.528,1.261)	0.421
Knee LoS Age 80+	-0.051 (-0.154,0.051)	0.327	-0.562 (-2.568,1.443)	0.583	0.028 (-0.105,0.161)	0.678	1.521 (-0.076,3.118)	0.062	1.37 (-0.178,2.918)	0.083	1.361 (-0.071,2.794)	0.063
Knee LoS Men	-0.007 (-0.037,0.023)	0.656	0.096 (-0.512,0.704)	0.758	-0.033 (-0.075,0.009)	0.121	0.082 (-0.382,0.547)	0.729	0.157 (-0.272,0.585)	0.474	0.205 (-0.265,0.675)	0.392
Knee LoS Women	-0.042 (-0.088,0.004)	0.071	0.252 (-0.495,1)	0.508	0.017 (-0.036,0.07)	0.536	0.635 (0.107,1.164)	0.019	0.15 (-0.365,0.666)	0.567	0.49 (-0.022,1.003)	0.061
Knee LoS Charlson 0	-0.021 (-0.054,0.012)	0.214	0.067 (-0.49,0.624)	0.814	-0.011 (-0.056,0.034)	0.643	0.645 (0.278,1.013)	0.001	0.193 (-0.146,0.533)	0.265	0.415 (-0.078,0.907)	0.099
Knee LoS Charlson 1	-0.03 (-0.081,0.022)	0.260	0.47 (-0.741,1.681)	0.447	-0.029 (-0.093,0.036)	0.384	0.455 (-0.111,1.021)	0.115	0.324 (-0.426,1.074)	0.398	1.058 (0.262,1.854)	0.009
Knee LoS Charlson 2+	-0.04 (-0.111,0.032)	0.276	0.407 (-2.137,2.951)	0.754	-0.022 (-0.184,0.14)	0.788	-0.627 (-1.883,0.628)	0.327	-0.949 (-2.145,0.247)	0.120	-1.433 (-2.278,-0.589)	0.001
Knee LoS Dep 1	-0.009 (-0.064,0.045)	0.733	-0.035 (-0.918,0.847)	0.937	-0.008 (-0.082,0.066)	0.826	0.257 (-0.443,0.958)	0.471	0.096 (-0.699,0.89)	0.813	-0.127 (-0.786,0.532)	0.706
Knee LoS Dep 2	-0.019 (-0.065,0.026)	0.413	0.199 (-1.031,1.429)	0.751	-0.043 (-0.116,0.031)	0.253	0.018 (-0.822,0.858)	0.967	-0.731 (-1.538,0.077)	0.076	0.05 (-0.819,0.919)	0.910
Knee LoS Dep 3	0.014 (-0.05,0.078)	0.670	-0.426 (-1.892,1.04)	0.569	-0.036 (-0.116,0.043)	0.369	0.976 (-0.09,2.043)	0.073	0.909 (-0.134,1.952)	0.088	0.547 (-0.408,1.503)	0.261
Knee LoS Dep 4	-0.064 (-0.143,0.015)	0.113	0.634 (-0.591,1.858)	0.310	0.033 (-0.057,0.123)	0.474	0.669 (-0.193,1.531)	0.128	0.617 (-0.032,1.266)	0.063	0.852 (-0.138,1.842)	0.092
Knee LoS Dep 5	-0.035 (-0.116,0.047)	0.405	0.709 (-0.701,2.119)	0.324	0.015 (-0.076,0.106)	0.745	0.123 (-0.882,1.128)	0.810	-0.387 (-1.379,0.606)	0.445	1.104 (-0.268,2.476)	0.115
Knee Bed Occ	0.993 (0.984,1.001)	0.074	0.834 (0.711,0.979)	0.027	1 (0.989,1.011)	0.993	1.42 (1.269,1.59)	0.000	1.373 (1.217,1.548)	0.000	1.465 (1.287,1.667)	0.000
Knee Public Private	0.006 (-0.02,0.031)	0.667	-0.476 (-1.026,0.074)	0.090	-0.015 (-0.04,0.009)	0.225	0.113 (-0.089,0.314)	0.274	0.276 (0.035,0.517)	0.025	0.131 (-0.071,0.332)	0.205
Elec Emerg Ratio	-0.005 (-0.013,0.002)	0.167	-0.322 (-0.451,-0.192)	0.000	-0.016 (-0.025,-0.006)	0.001	-0.008 (-0.117,0.101)	0.884	-0.053 (-0.174,0.068)	0.392	-0.028 (-0.128,0.073)	0.591

For peer review only

For peer review only - http://bmjopen.bmj.com/site/about/guidelines.xhtml

Supplementary Table T4. Interrupted time series model results with maximum auto-correlation lag 5

	pre-trend		level change		trend change		spring		summer		autumn	
	estimate (95% CI)	p	estimate (95% CI)	p	estimate (95% CI)	p	estimate (95% CI)	p	estimate (95% CI)	p	estimate (95% CI)	p
Hip Admissions	0.995 (0.987,1.003)	0.249	1.055 (0.917,1.215)	0.454	0.991 (0.978,1.005)	0.217	1.19 (1.04,1.362)	0.012	1.207 (1.104,1.32)	0.000	1.101 (0.993,1.221)	0.069
Hip Average Age	-0.015 (-0.094,0.065)	0.714	1.571 (0.241,2.901)	0.021	-0.06 (-0.168,0.048)	0.277	1.52 (-0.178,3.218)	0.079	2.09 (0.818,3.361)	0.001	1.031 (-0.639,2.701)	0.226
Hip Prop Women	0.999 (0.995,1.002)	0.503	0.966 (0.895,1.042)	0.367	1.007 (1.001,1.014)	0.027	1.023 (0.932,1.122)	0.636	1.088 (1,1.184)	0.051	0.981 (0.879,1.094)	0.727
Hip Prop 2+ Charlson	0.994 (0.985,1.004)	0.261	1.411 (1.116,1.785)	0.004	1.01 (1,1.02)	0.042	1.195 (0.979,1.459)	0.079	1.306 (1.111,1.535)	0.001	1.003 (0.792,1.27)	0.981
Hip Prop High Deprivation	1.003 (0.994,1.012)	0.551	1.027 (0.883,1.195)	0.729	1.004 (0.99,1.018)	0.577	0.937 (0.805,1.091)	0.400	0.877 (0.752,1.022)	0.092	0.996 (0.847,1.171)	0.961
Hip LoS	-0.006 (-0.029,0.017)	0.605	0.312 (-0.12,0.745)	0.157	-0.014 (-0.042,0.015)	0.346	0.502 (0.286,0.718)	0.000	0.135 (-0.191,0.461)	0.418	0.1 (-0.332,0.532)	0.650
Hip LoS Age 16-59	-0.003 (-0.026,0.02)	0.773	0.379 (-0.046,0.803)	0.080	-0.026 (-0.062,0.01)	0.152	0.223 (-0.113,0.559)	0.193	0.384 (0.09,0.679)	0.011	0.219 (-0.191,0.63)	0.295
Hip LoS Age 60-69	-0.004 (-0.031,0.024)	0.799	0.013 (-0.766,0.791)	0.975	0 (-0.051,0.05)	0.987	0.107 (-0.33,0.543)	0.632	0.158 (-0.451,0.766)	0.611	0.162 (-0.352,0.676)	0.537
Hip LoS Age 70-79	0.006 (-0.054,0.065)	0.853	-0.753 (-1.944,0.438)	0.215	0.007 (-0.056,0.069)	0.836	-0.433 (-1.343,0.476)	0.350	-0.842 (-1.91,0.226)	0.122	-0.865 (-2.063,0.334)	0.157
Hip LoS Age 80+	-0.068 (-0.147,0.012)	0.097	2.109 (0.803,3.414)	0.002	-0.002 (-0.111,0.107)	0.967	1.506 (0.499,2.513)	0.003	0.222 (-0.799,1.243)	0.670	1.003 (-0.111,2.116)	0.078
Hip LoS Men	-0.007 (-0.047,0.033)	0.732	0.347 (-0.44,1.135)	0.387	-0.02 (-0.073,0.033)	0.458	0.293 (-0.117,0.704)	0.161	0.289 (-0.193,0.771)	0.239	0.458 (-0.163,1.079)	0.149
Hip LoS Women	-0.004 (-0.035,0.028)	0.816	0.235 (-0.271,0.741)	0.362	-0.014 (-0.046,0.018)	0.391	0.693 (0.395,0.991)	0.000	0.009 (-0.421,0.439)	0.966	-0.203 (-0.762,0.357)	0.477
Hip LoS Charlson 0	-0.011 (-0.039,0.016)	0.418	0.297 (-0.242,0.837)	0.280	-0.005 (-0.035,0.025)	0.738	0.912 (0.617,1.208)	0.000	0.178 (-0.123,0.478)	0.247	0.222 (-0.066,0.51)	0.131
Hip LoS Charlson 1	0.034 (0.003,0.065)	0.030	0.238 (-0.604,1.08)	0.580	-0.077 (-0.137,-0.018)	0.011	0.191 (-0.385,0.767)	0.515	0.312 (-0.431,1.056)	0.410	0.014 (-0.563,0.591)	0.962
Hip LoS Charlson 2+	-0.123 (-0.239,-0.007)	0.038	0.172 (-1.97,2.314)	0.875	0.116 (0.005,0.228)	0.040	-0.789 (-2.189,0.611)	0.269	-0.783 (-2.119,0.553)	0.250	0.188 (-2.176,2.552)	0.876
Hip LoS Dep 1	0.004 (-0.029,0.037)	0.814	0.553 (-0.303,1.408)	0.206	-0.017 (-0.065,0.032)	0.501	0.784 (0.131,1.437)	0.019	0.42 (-0.034,0.874)	0.070	0.299 (-0.401,0.999)	0.402
Hip LoS Dep 2	-0.038 (-0.083,0.006)	0.092	0.822 (-0.167,1.81)	0.103	-0.015 (-0.078,0.047)	0.630	1.509 (0.737,2.28)	0.000	0.66 (-0.202,1.522)	0.133	0.589 (-0.461,1.638)	0.272
Hip LoS Dep 3	-0.034 (-0.102,0.033)	0.319	0.617 (-0.789,2.023)	0.390	0.01 (-0.072,0.091)	0.819	-0.216 (-1.011,0.579)	0.595	-0.424 (-1.293,0.445)	0.339	-0.701 (-1.351,-0.05)	0.035
Hip LoS Dep 4	0.081 (0.017,0.144)	0.013	-0.473 (-1.858,0.913)	0.504	-0.112 (-0.189,-0.035)	0.004	-0.374 (-1.59,0.842)	0.547	-1.204 (-2.355,-0.053)	0.040	-0.491 (-1.989,1.007)	0.520
Hip LoS Dep 5	0.015 (-0.056,0.085)	0.682	-0.72 (-1.647,0.208)	0.128	0 (-0.07,0.07)	0.998	0.621 (-0.104,1.347)	0.093	0.689 (-0.25,1.629)	0.150	0.816 (-0.696,2.329)	0.290
Hip Bed Occ	0.997 (0.987,1.008)	0.627	1 (0.862,1.161)	0.996	0.987 (0.97,1.004)	0.128	1.291 (1.116,1.492)	0.001	1.283 (1.14,1.444)	0.000	1.146 (0.99,1.326)	0.068
Hip Public Private	0.013 (-0.015,0.041)	0.377	-0.741 (-1.237,-0.245)	0.003	-0.019 (-0.05,0.011)	0.218	-0.008 (-0.212,0.196)	0.939	0.308 (0.154,0.463)	0.000	0.038 (-0.166,0.241)	0.718
Knee Admissions	0.995 (0.992,0.999)	0.016	0.843 (0.761,0.934)	0.001	1.005 (0.998,1.012)	0.170	1.308 (1.154,1.482)	0.000	1.26 (1.16,1.369)	0.000	1.286 (1.178,1.403)	0.000
Knee Average Age	-0.078 (-0.152,-0.005)	0.037	-1.632 (-2.825,-0.439)	0.007	0.211 (0.128,0.293)	0.000	0.926 (-0.072,1.924)	0.069	0.953 (0.077,1.829)	0.033	0.354 (-0.422,1.13)	0.371
Knee Prop Women	1.004 (0.999,1.009)	0.086	0.96 (0.861,1.071)	0.465	0.994 (0.987,1.001)	0.097	1.037 (0.973,1.105)	0.260	1.017 (0.922,1.121)	0.743	1.036 (0.966,1.111)	0.319
Knee Prop 2+ Charlson	1.009 (0.993,1.026)	0.250	0.638 (0.468,0.869)	0.004	1.042 (1.021,1.064)	0.000	1.156 (0.931,1.436)	0.190	1.074 (0.841,1.372)	0.567	0.909 (0.626,1.321)	0.617
Knee Prop High Deprivation	1.005 (0.999,1.011)	0.107	0.968 (0.835,1.122)	0.664	0.986 (0.977,0.995)	0.003	1.224 (1.082,1.384)	0.001	1.075 (0.95,1.217)	0.251	1.031 (0.924,1.149)	0.586
Knee LoS	-0.024 (-0.047,-0.001)	0.042	0.176 (-0.2,0.551)	0.360	-0.008 (-0.031,0.015)	0.490	0.422 (0.033,0.81)	0.033	0.15 (-0.199,0.499)	0.398	0.396 (-0.011,0.803)	0.057

For peer review only - http://bmjopen.bmj.com/site/about/guidelines.xhtml

Knee LoS Age 16-59	-0.016 (-0.032,0.001)	0.068	0.403 (0.008,0.799)	0.046	-0.028 (-0.059,0.003)	0.081	0.484 (0.198,0.77)	0.001	0.422 (-0.01,0.855)	0.056	0.475 (0.216,0.734)	0.000
Knee LoS Age 16-59	0.007 (-0.018,0.033)	0.571	-0.295 (-0.854,0.265)	0.302	-0.026 (-0.062,0.009)	0.147	0.069 (-0.455,0.593)	0.795	-0.194 (-0.652,0.265)	0.407	-0.096 (-0.713,0.52)	0.759
Knee LoS Age 70-79	-0.009 (-0.045,0.027)	0.616	0.77 (0.101,1.439)	0.024	-0.054 (-0.096,-0.012)	0.012	-0.086 (-0.736,0.563)	0.794	-0.552 (-1.116,0.012)	0.055	0.367 (-0.3,1.034)	0.281
Knee LoS Age 80+	-0.051 (-0.097,-0.006)	0.026	-0.562 (-1.849,0.724)	0.391	0.028 (-0.062,0.119)	0.541	1.521 (0.119,2.923)	0.034	1.37 (0.391,2.349)	0.006	1.361 (0.617,2.106)	0.000
Knee LoS Men	-0.007 (-0.028,0.014)	0.514	0.096 (-0.297,0.488)	0.633	-0.033 (-0.062,-0.004)	0.024	0.082 (-0.416,0.58)	0.746	0.157 (-0.244,0.558)	0.444	0.205 (-0.149,0.559)	0.256
Knee LoS Women	-0.042 (-0.079,-0.005)	0.027	0.252 (-0.356,0.861)	0.416	0.017 (-0.018,0.052)	0.350	0.635 (-0.036,1.307)	0.064	0.15 (-0.454,0.754)	0.625	0.49 (-0.258,1.239)	0.199
Knee LoS Charlson 0	-0.021 (-0.044,0.002)	0.077	0.067 (-0.312,0.446)	0.730	-0.011 (-0.036,0.015)	0.419	0.645 (0.331,0.959)	0.000	0.193 (-0.129,0.516)	0.240	0.415 (0.075,0.754)	0.017
Knee LoS Charlson 1	-0.03 (-0.064,0.005)	0.092	0.47 (-0.199,1.139)	0.169	-0.029 (-0.067,0.01)	0.145	0.455 (-0.097,1.007)	0.106	0.324 (-0.341,0.988)	0.340	1.058 (0.384,1.731)	0.002
Knee LoS Charlson 2+	-0.04 (-0.092,0.012)	0.134	0.407 (-1.956,2.771)	0.736	-0.022 (-0.184,0.14)	0.789	-0.627 (-1.625,0.37)	0.218	-0.949 (-2.374,0.476)	0.192	-1.433 (-2.175,-0.691)	0.000
Knee LoS Dep 1	-0.009 (-0.05,0.031)	0.644	-0.035 (-0.656,0.586)	0.911	-0.008 (-0.059,0.043)	0.750	0.257 (-0.541,1.056)	0.527	0.096 (-0.581,0.772)	0.782	-0.127 (-0.724,0.47)	0.677
Knee LoS Dep 2	-0.019 (-0.055,0.017)	0.300	0.199 (-0.57,0.968)	0.612	-0.043 (-0.086,0)	0.049	0.018 (-0.731,0.767)	0.962	-0.731 (-1.507,0.046)	0.065	0.05 (-0.887,0.987)	0.917
Knee LoS Dep 3	0.014 (-0.031,0.059)	0.544	-0.426 (-1.102,0.25)	0.217	-0.036 (-0.095,0.022)	0.220	0.976 (0.281,1.672)	0.006	0.909 (-0.067,1.886)	0.068	0.547 (-0.12,1.215)	0.108
Knee LoS Dep 4	-0.064 (-0.127,-0.001)	0.047	0.634 (-0.165,1.432)	0.120	0.033 (-0.04,0.106)	0.380	0.669 (-0.076,1.414)	0.079	0.617 (0.007,1.227)	0.047	0.852 (-0.274,1.978)	0.138
Knee LoS Dep 5	-0.035 (-0.087,0.018)	0.194	0.709 (-0.225,1.643)	0.137	0.015 (-0.042,0.072)	0.606	0.123 (-0.701,0.947)	0.770	-0.387 (-1.304,0.531)	0.409	1.104 (0.188,2.02)	0.018
Knee Bed Occ	0.993 (0.986,0.999)	0.032	0.834 (0.728,0.956)	0.009	1 (0.991,1.009)	0.991	1.42 (1.308,1.542)	0.000	1.373 (1.213,1.553)	0.000	1.465 (1.318,1.627)	0.000
Knee Public Private	0.006 (-0.02,0.031)	0.667	-0.476 (-1.026,0.074)	0.090	-0.015 (-0.04,0.009)	0.225	0.113 (-0.089,0.314)	0.274	0.276 (0.035,0.517)	0.025	0.131 (-0.071,0.332)	0.205
Elec Emerg Ratio	-0.005 (-0.013,0.003)	0.198	-0.322 (-0.455,-0.189)	0.000	-0.016 (-0.026,-0.005)	0.004	-0.008 (-0.107,0.091)	0.872	-0.053 (-0.18,0.074)	0.414	-0.028 (-0.163,0.108)	0.690

For peer review only - http://bmjopen.bmj.com/site/about/guidelines.xhtml

The RECORD statement – checklist of items, extended from the STROBE statement, that should be reported in observational studies using routinely collected health data.

	Item No.	STROBE items	Location in manuscript where items are reported	RECORD items	Location in manuscript where items are reported
**Title and abstract**					
	1	(a) Indicate the study's design with a commonly used term in the title or the abstract (b) Provide in the abstract an informative and balanced summary of what was done and what was found		RECORD 1.1: The type of data used should be specified in the title or abstract. When possible, the name of the databases used should be included.<br><br>RECORD 1.2: If applicable, the geographic region and timeframe within which the study took place should be reported in the title or abstract.<br><br>RECORD 1.3: If linkage between databases was conducted for the study, this should be clearly stated in the title or abstract.	Abstract P2 L35-37<br><br>Abstract P2 L35-38
**Introduction**					
Background rationale	2	Explain the scientific background and rationale for the investigation being reported			Introduction P4-5 L76-113
Objectives	3	State specific objectives, including any prespecified hypotheses			Introduction P5 L107-113
**Methods**					
Study Design	4	Present key elements of study design early in the paper			Methods P5 L116-119
Setting	5	Describe the setting, locations, and relevant dates, including periods of recruitment, exposure, follow-up, and data collection			Methods P5 L116-119 Methods P7-8 L70-176

For peer review only

Participants	6	*(a) Cohort study* - Give the eligibility criteria, and the sources and methods of selection of participants. Describe methods of follow-up<br>*Case-control study* - Give the eligibility criteria, and the sources and methods of case ascertainment and control selection. Give the rationale for the choice of cases and controls<br>*Cross-sectional study* - Give the eligibility criteria, and the sources and methods of selection of participants<br><br>*(b) Cohort study* - For matched studies, give matching criteria and number of exposed and unexposed<br>*Case-control study* - For matched studies, give matching criteria and the number of controls per case		RECORD 6.1: The methods of study population selection (such as codes or algorithms used to identify subjects) should be listed in detail. If this is not possible, an explanation should be provided.<br><br>RECORD 6.2: Any validation studies of the codes or algorithms used to select the population should be referenced. If validation was conducted for this study and not published elsewhere, detailed methods and results should be provided.<br><br>RECORD 6.3: If the study involved linkage of databases, consider use of a flow diagram or other graphical display to demonstrate the data linkage process, including the number of individuals with linked data at each stage.	Methods P6 L141-147 Supplementary Table T1
Variables	7	Clearly define all outcomes, exposures, predictors, potential confounders, and effect modifiers. Give diagnostic criteria, if applicable.		RECORD 7.1: A complete list of codes and algorithms used to classify exposures, outcomes, confounders, and effect modifiers should be provided. If these cannot be reported, an explanation should be provided.	Methods P6-7 L141-169
Data sources/ measurement	8	For each variable of interest, give sources of data and details of methods of assessment (measurement).<br>Describe comparability of assessment methods if there is more than one group			Methods P5-6 L120-140

For peer review only

Bias	9	Describe any efforts to address potential sources of bias			Methods P7-8 L170-185,
Study size	10	Explain how the study size was arrived at			Methods P6 L141-147
Quantitative variables	11	Explain how quantitative variables were handled in the analyses. If applicable, describe which groupings were chosen, and why			Methods P6-7 L141-169
Statistical methods	12	(a) Describe all statistical methods, including those used to control for confounding<br>(b) Describe any methods used to examine subgroups and interactions<br>(c) Explain how missing data were addressed<br>(d) *Cohort study* - If applicable, explain how loss to follow-up was addressed<br>*Case-control study* - If applicable, explain how matching of cases and controls was addressed<br>*Cross-sectional study* - If applicable, describe analytical methods taking account of sampling strategy<br>(e) Describe any sensitivity analyses			Methods P7-8 L170-185
Data access and cleaning methods		..		RECORD 12.1: Authors should describe the extent to which the investigators had access to the database population used to create the study population.	Data Sharing P17 L392-400

For peer review only

				RECORD 12.2: Authors should provide information on the data cleaning methods used in the study.	Methods P6-7 L141-169
Linkage		..		RECORD 12.3: State whether the study included person-level, institutional-level, or other data linkage across two or more databases. The methods of linkage and methods of linkage quality evaluation should be provided.	N/A
**Results**					
Participants	13	(a) Report the numbers of individuals at each stage of the study (*e.g.*, numbers potentially eligible, examined for eligibility, confirmed eligible, included in the study, completing follow-up, and analysed)<br>(b) Give reasons for non-participation at each stage.<br>(c) Consider use of a flow diagram		RECORD 13.1: Describe in detail the selection of the persons included in the study (*i.e.,* study population selection) including filtering based on data quality, data availability and linkage. The selection of included persons can be described in the text and/or by means of the study flow diagram.	Methods P6 L141-147<br><br>Results P8 L191-194
Descriptive data	14	(a) Give characteristics of study participants (*e.g.*, demographic, clinical, social) and information on exposures and potential confounders<br>(b) Indicate the number of participants with missing data for each variable of interest<br>(c) *Cohort study* - summarise follow-up time (*e.g.*, average and total amount)			Results P8 L191-194
Outcome data	15	*Cohort study* - Report numbers of outcome events or summary measures over time<br>*Case-control study* - Report numbers in each exposure			Results P8-9 L195-206

For peer review only

		category, or summary measures of exposure<br>*Cross-sectional study* - Report numbers of outcome events or summary measures			
Main results	16	(a) Give unadjusted estimates and, if applicable, confounder-adjusted estimates and their precision (e.g., 95% confidence interval). Make clear which confounders were adjusted for and why they were included<br>(b) Report category boundaries when continuous variables were categorized<br>(c) If relevant, consider translating estimates of relative risk into absolute risk for a meaningful time period			Results P9-11 L196-267
Other analyses	17	Report other analyses done—e.g., analyses of subgroups and interactions, and sensitivity analyses			Results P9-11 L196-267
**Discussion**					
Key results	18	Summarise key results with reference to study objectives			Discussion P11-12, L268-288
Limitations	19	Discuss limitations of the study, taking into account sources of potential bias or imprecision. Discuss both direction and magnitude of any potential bias		RECORD 19.1: Discuss the implications of using data that were not created or collected to answer the specific research question(s). Include discussion of misclassification bias, unmeasured confounding, missing data, and changing eligibility over time, as they pertain to the study being reported.	Discussion P12-13 L289-303
Interpretation	20	Give a cautious overall interpretation of results considering objectives,			Discussion P13-14 L317-340

For peer review only

		limitations, multiplicity of analyses, results from similar studies, and other relevant evidence			
Generalisability	21	Discuss the generalisability (external validity) of the study results			Discussion P13 L294-303
**Other Information**					
Funding	22	Give the source of funding and the role of the funders for the present study and, if applicable, for the original study on which the present article is based			Funding Statement P16 L375-380
Accessibility of protocol, raw data, and programming code		..		RECORD 22.1: Authors should provide information on how to access any supplemental information such as the study protocol, raw data, or programming code.	Methods P8 L186-188 Data Sharing P17 L392-400

*Reference: Benchimol EI, Smeeth L, Guttmann A, Harron K, Moher D, Petersen I, Sørensen HT, von Elm E, Langan SM, the RECORD Working Committee. The REporting of studies Conducted using Observational Routinely-collected health Data (RECORD) Statement. *PLoS Medicine* 2015; in press.

*Checklist is protected under Creative Commons Attribution (CC BY) license.

For peer review only

BMJ Open

The impact of pausing elective hip and knee replacement surgery during winter 2017 on subsequent service provision at a major NHS Trust: a descriptive observational study using interrupted time series

Journal:	*BMJ Open*
Manuscript ID	bmjopen-2022-066398.R2
Article Type:	Original research
Date Submitted by the Author:	30-Mar-2023
Complete List of Authors:	Jones, Tim; University Hospitals Bristol and Weston NHS Foundation Trust, NIHR ARC West; University of Bristol, Department of Population Health Sciences<br>Penfold, Chris; University Hospitals Bristol and Weston NHS Foundation Trust, NIHR ARC West; University of Bristol, Musculoskeletal Research Unit<br>Redaniel, Maria Theresa; University Hospitals Bristol and Weston NHS Foundation Trust, NIHR ARC West; University of Bristol, Population Health Sciences, Bristol Medical School<br>Eyles, Emily; University Hospitals Bristol and Weston NHS Foundation Trust, NIHR ARC West; University of Bristol, Population Health Sciences, Bristol Medical School<br>Keen, Tim; North Bristol NHS Trust<br>Elliott, Andrew; North Bristol NHS Trust<br>Blom, AW; University of Bristol, Translational Health Sciences, Bristol Medical School<br>Judge, Andrew; University of Bristol, Musculoskeletal Research Unit
**Primary Subject Heading**:	Health services research
Secondary Subject Heading:	Surgery
Keywords:	Knee < ORTHOPAEDIC & TRAUMA SURGERY, Hip < ORTHOPAEDIC & TRAUMA SURGERY, Orthopaedic & trauma surgery < SURGERY, Human resource management < HEALTH SERVICES ADMINISTRATION & MANAGEMENT

SCHOLARONE™
Manuscripts

I, the Submitting Author has the right to grant and does grant on behalf of all authors of the Work (as defined in the below author licence), an exclusive licence and/or a non-exclusive licence for contributions from authors who are: i) UK Crown employees; ii) where BMJ has agreed a CC-BY licence shall apply, and/or iii) in accordance with the terms applicable for US Federal Government officers or employees acting as part of their official duties; on a worldwide, perpetual, irrevocable, royalty-free basis to BMJ Publishing Group Ltd ("BMJ") its licensees and where the relevant Journal is co-owned by BMJ to the co-owners of the Journal, to publish the Work in this journal and any other BMJ products and to exploit all rights, as set out in our licence.

The Submitting Author accepts and understands that any supply made under these terms is made by BMJ to the Submitting Author unless you are acting as an employee on behalf of your employer or a postgraduate student of an affiliated institution which is paying any applicable article publishing charge ("APC") for Open Access articles. Where the Submitting Author wishes to make the Work available on an Open Access basis (and intends to pay the relevant APC), the terms of reuse of such Open Access shall be governed by a Creative Commons licence – details of these licences and which Creative Commons licence will apply to this Work are set out in our licence referred to above.

Other than as permitted in any relevant BMJ Author's Self Archiving Policies, I confirm this Work has not been accepted for publication elsewhere, is not being considered for publication elsewhere and does not duplicate material already published. I confirm all authors consent to publication of this Work and authorise the granting of this licence.

For peer review only

Title: The impact of pausing elective hip and knee replacement surgery during winter 2017 on subsequent service provision at a major NHS Trust: a descriptive observational study using interrupted time series

Authors: Tim Jones[1,2,3], Christopher Penfold[1,2,3], Maria Theresa Redaniel[1,2], Emily Eyles[1,2], Tim Keen[4], Andrew Elliott[4], Ashley Blom[2,3], Andrew Judge[3,5]

[1] NIHR ARC West, University Hospitals Bristol NHS Foundation Trust, Bristol, BS1 2NT, UK

[2] Department of Population Health Sciences, Bristol Medical School, University of Bristol, BS8 2PS, UK

[3] Musculoskeletal Research Unit, Translational Health Sciences, Bristol Medical School, University of Bristol, Learning and Research Building, Level 1, Southmead Hospital, Bristol, BS10 5NB, UK

[4] North Bristol NHS Trust, Southmead Hospital, Westbury-on-Trym, Bristol, BS10 5NB, UK

[5] National Institute for Health Research Bristol Biomedical Research Centre, University Hospitals Bristol and Weston NHS Foundation Trust and University of Bristol, Bristol, UK

Corresponding author:

Tim Jones
Research Fellow
NIHR ARC West,
9th Floor Whitefriars,
Lewin's Mead
Bristol, BS1 2NT
United Kingdom

Tel: ++44 (0)117 342 1265
Timothy.Jones@bristol.ac.uk

Word count [3,957]

For peer review only - http://bmjopen.bmj.com/site/about/guidelines.xhtml

ABSTRACT

Objectives: To explore the impact of a temporary cancellation of elective surgery in winter 2017 on trends in primary hip and knee replacement at a major NHS Trust, and whether lessons can be learnt about efficient surgery provision.

Design and Setting: Observational descriptive study using interrupted time series analysis of hospital records to explore trends in primary hip and knee replacement surgery at a major NHS Trust, as well as patient characteristics, 2016-2019.

Intervention: A temporary cancellation of elective services for two months in winter 2017

Outcomes: NHS-funded hospital admissions for primary hip or knee replacement, length of stay and bed occupancy. Additionally, we explored the ratio of elective to emergency admissions at the Trust as a measure of elective capacity, and the ratio of public to private provision of NHS-funded hip and knee surgery.

Results: After winter 2017 there was a sustained reduction in the number of knee replacements, a decrease in the proportion of most deprived people having knee replacements, and an increase in average age for knee replacement and comorbidity for both types of surgery. The ratio of public to private provision dropped after winter 2017, and elective capacity generally has reduced over time. There was clear seasonality in provision of elective surgery, with less-complex patients admitted during winter.

Conclusions: Declining elective capacity and seasonality has a marked effect on the provision of joint replacement, despite efficiency improvements in hospital treatment. The Trust has outsourced less complex patients to independent providers, and/or treated them during winter when capacity is most limited. There is a need to explore whether these are strategies that could be used explicitly to maximise the use of limited elective capacity, provide benefit to patients, and value for money for taxpayers.

For peer review only - http://bmjopen.bmj.com/site/about/guidelines.xhtml

STRENGTH AND LIMITATIONS OF THIS STUDY

- Trends analyses using data obtained from the electronic health records of a local hospital NHS Trust are informative for clinicians and service managers in monitoring changes in planning and delivery of elective surgery, and could be regularly updated in near real time for monitoring.
- The inclusion of wider hospital admissions data beyond the NHS Trust allows us to estimate the proportion of people within the Trust catchment area having NHS-funded treatment at independent providers.
- We report the experience of one NHS Trust that is one of the larger elective orthopaedic centres - the findings may not be generalisable to or reflect the experience of other trusts.
- Our study does not include privately funded, privately provided hip and knee surgery which may also have been changing over time.

For peer review only

INTRODUCTION

Primary hip and knee replacement operations are common planned elective surgical procedures. They are highly clinically effective for improving symptoms of pain and functional limitations, and have been shown to be safe and cost-effective.[1-4] Around 100,000 hip[4] and over 100,000 knee operations[3] are carried out each year in the UK. Demand for these operations has been increasing substantially in recent decades[5] with an ageing population, rising levels of obesity, and widening indications for surgery in younger patient groups.[3 4]

Orthopaedic services have become more efficient over time, with length of hospital stay for primary hip and knee replacements reducing from around 15 days in 1997 to roughly 5.5 days in 2014.[6] This is largely due to the introduction of 'fast track' surgery and enhanced recovery services,[7] which reduce length of stay whilst maintaining patient safety and outcomes of surgery.[6] However, over the past decade there has also been a reduction in the numbers of hospital beds and operating theatres available for hip and knee replacement patients.[8] Waiting lists for orthopaedic procedures have been growing over time, and the average time people wait for treatment once on the waiting list has also increased.[9]

Pressures on elective surgery are exacerbated during winter, when resources for planned surgery are often displaced by more acute, unplanned hospital admissions.[8] At the end of 2017, this led to all planned elective hip and knee replacement operations in England being cancelled for the whole of January.[10] Even before the COVID-19 pandemic, over half a million people were already on the waiting list.[11] Patients are having to wait longer with deteriorating severe pain and functional limitation, affecting their health and quality of life. The COVID-19 pandemic has had an even greater impact on cancelling planned elective surgery, with over 635,000 people waiting for hip and knee replacements in April 2021, more than 10% of these waiting over a year, and over a third waiting longer than the 18 week target.[11]

For peer review only

The winter of 2017 provides a form of 'natural experiment', where elective capacity was intentionally reduced close to zero. A natural experimental design is a valid methodological approach to evaluate the impact of a range of events, policies and interventions which are not under the control of researchers.[12] Researchers can use the variation in exposure that natural experiments generate to analyse their impact on health outcomes. This provides a form of quasi-experimental study, where we can explore trends in provision of elective surgery before and after Winter 2017, which is a robust approach to explore real-world impact when randomisation is not possible.[13 14]

Our aim was to understand what happens after common, planned elective surgery is temporarily cancelled, and how this might inform optimum planning of elective surgery when capacity is limited, such as following the COVID-19 pandemic. We used interrupted time series analysis to model trends in elective hip and knee replacement surgery for a major NHS Trust from 2016 to 2019 and see how these were impacted by the withdrawal of elective surgery in winter 2017. We explored these trends by patient factors (age, sex, deprivation, number of comorbidities) and seasonality to see when demand was highest for different patient groups.

METHODS

This study is a longitudinal observational descriptive study using routinely collected administrative information about patients admitted to a major NHS Trust for elective hip and knee replacements, 2016 to 2019. It was developed and reported according to the RECORD extension[15] to STROBE guidelines for observational studies using routinely collected data.

Data Sources

We used two data sources for our analyses. The first was an extract of elective primary hip and knee replacement inpatient admissions identified from the Trust's electronic medical records (EMR) between 1st January 2016 and 31st December 2019. Up to 29 diagnoses were provided per entry using the International Classification of Diseases version 10 (ICD-10), and up to 11 procedures were provided

For peer review only - http://bmjopen.bmj.com/site/about/guidelines.xhtml

per entry using the Office of Population Censuses and Surveys Classification of Interventions and Procedures version 4 (OPCS-4). The extract included patient demographics such as age, sex, deprivation quintile, and comorbidities; and other characteristics of the hospital admissions such as length of stay. This data source was used for all analyses of hip and knee replacements at the Trust, including those relating to patient demographics, length of stay, and bed occupancy.

The second data source was pseudonymised national admitted patient care Hospital Episode Statistics (HES-APC) between 1st January 2016 and 31st December 2019. HES-APC is a routinely collected dataset that records all episodes of admitted (day case or inpatient) care provided to patients at NHS hospitals in England and to NHS-funded patients treated in independent hospitals.[16] Each episode represents a period of care under one consultant team. Up to 20 diagnoses and 24 clinical procedures are recorded per episode using ICD-10 codes and OPCS-4 codes, respectively. HES also includes the Lower Super Output Area (LSOA; an area of around 1,500 people) of residence for each patient, which can be linked to CCG of residence. This data source was used to estimate elective capacity overall at the Trust, and the ratio of public/private provision of hip and knee replacements in the catchment area for the Trust (see details below), which could not be gathered from the extract provided from the Trust EMR.

Hospital admissions for hip and knee replacements

Hospital admissions for elective hip and knee replacements were identified by entries with a primary procedure code representing primary hip or knee replacement (Supplementary Table T1) using the Trust EMR. We used this information to explore summary characteristics of the hospital admissions over time (overall counts of admissions, average age, proportion of women, proportion with 2+ comorbidities, proportion in the two most deprived quintiles) stratified by primary hip or knee replacements.

Length of stay and bed occupancy

For peer review only - http://bmjopen.bmj.com/site/about/guidelines.xhtml

We used the average number of overnight stays in hospital (days) for length of stay, trimmed at 30 days to exclude a small number of outliers (n=32, 0.6%). Trimming allowed us to model averages assuming a roughly normal distribution which we felt was more easily interpretable. Bed occupancy was the total number of beds used overnight for hip and knee replacement patients.

Comorbidity of Admissions

For each admission, we counted the number of conditions from the Charlson comorbidity index[17] recorded in the diagnosis fields. The Charlson index provides a summary of weighted scores relating to different comorbidities and has been shown to be associated with mortality. Admissions were categorised into those with zero, one, and two or more Charlson comorbidities.

Ratio of Elective to Emergency Admissions

To estimate the ratio of elective to emergency admissions for all purposes at the Trust (as a proxy for elective capacity), we extracted all hospital admissions from HES-APC with the Trust as a provider and categorised them into elective and emergency (admission method beginning with '1' or '2', respectively).

Ratio of Public to Private Provision of Hip and Knee Replacements

To estimate the ratio of public to private provision of NHS-funded elective hip and knee surgery for the Trust catchment area, we extracted all hospital admissions for primary hip and knee replacements (codes in Supplementary Table T1) for residents of the major local clinical commissioning groups (CCGs) from HES-APC (using 2021 CCG boundaries after local CCGs had merged into one CCG[18]), and categorised providers into public and private (provider code beginning with 'R' or 'N', respectively).

Statistical Analysis

We explored the change in trend for the following outcomes before/after the winter 2017 cancellation of elective surgery, stratified by primary hip and knee replacements: number of hospital admissions;

For peer review only

average age of patients; proportion of women; proportion with 2+ comorbidities; proportion in more deprived deprivation quintiles (4 and 5); average length of stay; bed occupancy; and ratio of public to private provision of surgery. Additionally, we explored the overall ratio of elective to emergency admissions at the hospital for any purpose without stratification. For each of the outcomes, we conducted interrupted time series (ITS) analyses using segmented regression models comparing hospital admissions in the 'before' period (January 2016 to November 2017) to the 'after' period (February 2018 to December 2019). We excluded the winter 2017 period when admissions were very low (December 2017 and January 2018). The ITS analyses explored the 'pre-trend' before winter 2017, and how this trend changed after winter 2017[12 19], allowing for an immediate 'level change' up or down in February 2018, and a longer-term 'trend change' in the slope afterwards. We explored seasonality in the data by including indicator variables for spring, summer, and autumn[19] compared to winter as a baseline, and adjusted for serial autocorrelation using Newey-West standard errors with a maximum lag of two[20-22]. For count or proportion outcomes (number of admissions, proportion women, proportion with 2+ comorbidities, proportion in top two deprivation quintiles, bed occupancy) segmented Poisson regression models were fit to the data, whilst for averages/ratios (average age, average length of stay, ratio of elective to emergency admissions, ratio of public to private provision) segmented linear regression models were fit, using the 'glm' command in Stata. Sensitivity analyses were conducted adjusting the maximum lag for serial autocorrelation to zero and five; this would not affect point estimates but could alter standard errors, confidence intervals, and p-values.

All statistical analyses were conducted using Stata/MP version 16.1. Smoothed trends were fit to the data on all plots using the 'lowess' command with bandwidth 0.3. Stata code is available at: https://github.com/jonestim2002/hdr_uk_hospital_efficiency

Patient and public involvement

For peer review only - http://bmjopen.bmj.com/site/about/guidelines.xhtml

Initial research ideas for the grant application of which this work is part were presented to the public in a workshop and suggestions and comments were incorporated in the protocol. Feedback during the workshop was positive, with participants agreeing with the research objectives and the identified need.

RESULTS

Descriptive information and demographics

A total of 2,623 patients had a hip replacement and 2,674 had a knee replacement at the Trust in the 4 years between 2016 and 2019. The mean age of patients was 67 years and 60% were women for both types of operations.

Trend changes after winter 2017

Table 1 shows the results of our interrupted time series analyses for all outcomes, including the trend before winter 2017 (pre-trend), any immediate change after winter 2017 (level change) and any change in the slope after winter 2017 (trend change). These are described in more detail below.

Table 1. Interrupted time series model results

	pre-trend		level change		trend change	
	estimate (95% CI)	p	estimate (95% CI)	p	estimate (95% CI)	p
Hip Admissions	1 (0.99,1)	0.300	1.06 (0.91,1.22)	0.469	0.99 (0.98,1.01)	0.239
Hip Age*	-0.01 (-0.1,0.07)	0.737	1.57 (-0.1,3.24)	0.065	-0.06 (-0.17,0.05)	0.307
Hip Prop Women	1 (0.99,1)	0.582	0.97 (0.86,1.08)	0.549	1.01 (1,1.02)	0.089
Hip Charlson	0.99 (0.98,1.01)	0.380	1.41 (1.06,1.87)	0.017	1.01 (0.99,1.03)	0.220
Hip Deprivation	1 (0.99,1.01)	0.587	1.03 (0.87,1.21)	0.754	1 (0.99,1.02)	0.660
Hip LoS*	-0.01 (-0.03,0.02)	0.660	0.31 (-0.19,0.82)	0.225	-0.01 (-0.05,0.02)	0.425
Hip Bed Occ	1 (0.99,1.01)	0.643	1 (0.84,1.19)	0.997	0.99 (0.97,1)	0.149
Hip Public Private*	0.01 (-0.02,0.04)	0.377	-0.74 (-1.24,-0.25)	0.003	-0.02 (-0.05,0.01)	0.218
Knee Admissions	1 (0.99,1)	0.106	0.84 (0.73,0.98)	0.022	1 (1,1.01)	0.256
Knee Age*	-0.08 (-0.16,0)	0.054	-1.63 (-2.99,-0.28)	0.018	0.21 (0.12,0.31)	0.000

For peer review only - http://bmjopen.bmj.com/site/about/guidelines.xhtml

Knee Prop Women	1 (1,1.01)	0.150	0.96 (0.85,1.08)	0.513	0.99 (0.99,1)	0.193
Knee Charlson	1.01 (0.99,1.03)	0.249	0.64 (0.46,0.89)	0.009	1.04 (1.02,1.07)	0.001
Knee Deprivation	1.01 (1,1.01)	0.189	0.97 (0.79,1.19)	0.758	0.99 (0.97,1)	0.021
Knee LoS*	-0.02 (-0.05,0)	0.058	0.18 (-0.28,0.63)	0.449	-0.01 (-0.04,0.02)	0.566
Knee Bed Occ	0.99 (0.98,1)	0.103	0.83 (0.7,0.99)	0.037	1 (0.99,1.01)	0.993
Knee Public Private*	0.01 (-0.02,0.03)	0.667	-0.48 (-1.03,0.07)	0.090	-0.02 (-0.04,0.01)	0.225
Elec Emerg Ratio*	-0.01 (-0.01,0)	0.171	-0.32 (-0.45,-0.2)	0.000	-0.02 (-0.03,-0.01)	0.003

*Notes: *Linear regression model (additive) rather than Poisson regression model (multiplicative). Shaded cells indicate p < 0.05.*

Trends in hip and knee elective hospital admissions over time

The overall numbers of elective primary hip and knee replacement operations gradually declined over the study period from 63 hip and 65 knee replacements per month in 2016 to 49 hip and 51 knee replacements per month in 2019. Whilst there was a drop off in winter 2017, after elective surgery was re-started hip replacements resumed at similar numbers and continued to decline along a similar trajectory. Numbers of knee replacements dropped by 16% after winter 2017 (level change=0.843, 95% CI: 0.728 to 0.976, p=0.022), and the slope appeared to level off, although there was little evidence for this in the regression model (trend change=1.005, 95% CI: 0.996 to 1.014, p=0.256); see Figure 1 and Supplementary Table T2.

Age on Admission

There was a change in the trend in average age for knee replacements after winter 2017 (trend change=+0.21, 95% CI: 0.12 to 0.31, p < 0.001) towards treating older patients over time (+1.59 years of age per year); see Figure 2.

Comorbidity of Admissions

There was a level change upwards in the proportion having hip replacements with 2+ comorbidities after winter 2017 (level change=1.411, 95% CI: 1.064 to 1.873, p=0.017), and an upward slope change for knee replacements (trend change=1.042, 95% CI: 1.017 to 1.067, p=0.001); see Figure 3.

For peer review only

For peer review only - http://bmjopen.bmj.com/site/about/guidelines.xhtml

Deprivation

There was evidence of a reducing proportion of the most deprived people having knee replacements after winter 2017 (trend change=0.986, 95% CI: 0.974 to 0.998, p=0.021).

Ratio of elective admissions to emergency admissions at the Trust

There was an overall downward trend in the ratio of elective to emergency admissions at the Trust, from an average of 2.91 (SD: 0.17) electives for every emergency in 2016 to 2.16 (SD: 0.06) in 2019; see Supplementary Figure F1. The ratio reduced after winter 2017 (level change=-0.322, 95% CI: -0.446 to -0.198, p<0.001), and started to decrease more rapidly afterwards (trend change=-0.016, 95% CI: -0.026 to -0.005, p=0.003).

Ratio of public to private provision of hip/knee elective surgery at the Trust

There was evidence of a level change downwards in public provision compared to private provision after winter 2017 for both types of surgery, but particularly for hip replacements (hips level change=-0.741, 95% CI: -1.237 to -0.245, p=0.003; knees level change=-0.476, 95% CI: -1.026 to +0.074, p=0.09); see Figure 4.

Bed Occupancy

For hip and knee replacements, bed occupancy has reduced over time, although there wasn't evidence of this in the regression model for hip replacements, and there was a level change downwards (level change=0.834, 95% CI: 0.704 to 0.989, p=0.037) for knee surgery after winter 2017; see Figure 5.

For peer review only - http://bmjopen.bmj.com/site/about/guidelines.xhtml

Length of Stay

The average length of hospital stay was 5.5 days (SD: 5.9 days) for hip replacements and 5.2 days (SD: 5.0 days) for knee replacements in 2016, compared to 5.1 days (SD: 4.1 days) and 4.3 days (SD: 3.4 days) respectively in 2019 (see Supplementary Figure F2). However, there was no evidence in the regression models for a change after winter 2017.

Seasonality

Supplementary Table T2 shows seasonality results for each of our interrupted time series analyses. Hip and knee operations were clearly seasonal, with higher admissions in non-winter months compared to winter; 21% higher in the highest season (summer) for hips (summer=1.207, 95% CI: 1.094 to 1.332, p<0.001), and 31% higher in the highest season (spring) for knee replacements (spring=1.308, 95% CI: 1.157 to 1.479, p<0.001), excluding winter 2017. Bed occupancy for both types of operation was also seasonal, with lower occupancy in the winter months compared to all other seasons (see Supplementary Table T2); e.g. summer bed occupancy was 324 beds for hips and 291 beds for knees on average compared to winter bed occupancy of 225 beds for hips and 199 beds for knees on average. Length of stay was longer in spring than winter for hip replacements (spring=+0.502 days, 95% CI: 0.214 to 0.79, p=0.001), and longer in spring (+0.422 days, 95% CI: 0.073 to 0.771, p=0.018) and autumn (+0.396 days, 95% CI: 0.015 to 0.777, p=0.042) compared to winter for knee replacements.

The ratio of public to private provision was higher in the summer (1.56 for hips and 1.28 for knees) compared to winter (1.22 and 0.99, respectively) months (hips summer=+0.308, 95% CI 0.154 to 0.463, p<0.001; knees summer=+0.276, 95% CI: 0.035 to 0.517, p=0.025).

There was also some evidence of seasonality in the types of patients being admitted for hip and knee replacements. For hip replacements, the mean age of patients was 66 in winter compared to

For peer review only

68 in summer (summer=+2.09; 95% CI: 0.81 to 3.37, p = 0.001); a higher proportion were performed on women in the summer (64%) compared to winter (58%) months (summer=1.088, 95% CI: 1.001 to 1.183, p=0.048); and a higher proportion of people had 2+ comorbidities in the summer (15.9%) compared to winter (12.3%) months (summer=1.306, 95% CI: 1.096 to 1.557, p=0.003). For knee replacements, there was a higher proportion of more deprived people (quintiles 4 and 5) in the spring (37.6%) compared to the winter (30.2%) months (spring=1.224, 95% CI: 1.077 to 1.49, p=0.002).

DISCUSSION

Principal findings

The temporary cancellation of elective services during winter 2017 does appear to have had some impact on service provision at the Trust after that time. There was an immediate and sustained reduction in the number of knee replacements being done at the Trust and this was also reflected in the drop in bed occupancy for knee surgery. The average age for knee replacement and comorbidity of hip and knee surgery patients increased after winter 2017, whilst the proportion of more deprived people having knee replacements decreased, and the ratio of public to private provision of hip and knee replacements in the local area dropped after winter 2017. This suggests an NHS-funded outsourcing of less comorbid hip and knee replacement surgery to independent providers, and therefore on average the patients being treated at the Trust became older and more comorbid. There was a general decrease in capacity for elective surgery at the Trust (ratio of elective to emergency admissions), mostly driven by increasing non-elective admissions even before the COVID-19 pandemic. The winter 2017 cancellation may have been just one symptom of this overall pressure on elective surgery that underlies some of the longer-term changes in provision.

For peer review only - http://bmjopen.bmj.com/site/about/guidelines.xhtml

There was also some seasonality in service provision. It is no surprise that elective admissions and bed occupancy are lower in winter when the hospital requires capacity for an increase in unplanned admissions. There were also indications that people being admitted in winter were younger, less comorbid, and less deprived (particularly for knee surgery). Length of stay for hip and knee replacements was lower in winter compared to spring. This suggests the admission of younger, less comorbid patients during the winter months given the reduced elective capacity and delaying surgery for more comorbid patients to when capacity is higher in the following months.

Strengths and limitations

Trends analyses such as these, using data obtained from the EHR of a local hospital NHS Trust, are informative for clinicians and service managers in monitoring changes in planning and delivery of elective surgery, and could be regularly updated in near real time for monitoring. This concept might be informative for other commissioning groups / Trusts to adopt for monitoring of their own elective surgery and capacity. We report the experience of just one trust that is one of the larger elective orthopaedic centres, and hence the findings may not be generalisable to or reflect the experience of other trusts. Our findings are observational and report changes observed at the Trust following cancellation of elective services in winter 2017; further work would be needed to understand the impact of any changes on outcomes such as throughput of patients, waiting times, waiting lists, outcomes of surgery, costs, and equity of access to surgery. There is likely to be some correlation between the covariables explored; for example, older people tend to be less deprived and have more comorbidities, which may account for some of our results. Some of the increase in age at operation after winter 2017 may be due to increased waiting times for surgery. We should be aware that some results may reflect chance findings due to multiple testing and type 1 error. The trends in the data as plotted do not change substantially in sensitivity analyses accounting for different autocorrelation lags (Supplementary Tables T3-T4). The catchment area of the Trust is not exactly the same as the major local CCG and is difficult to define exactly. However, 89.4% of admissions at

For peer review only - http://bmjopen.bmj.com/site/about/guidelines.xhtml

the Trust were for residents of the local CCG and we felt this was a reasonable approximation to estimate the ratio of public to private provision in the Trust catchment area. Our analyses only include NHS-funded surgery and not privately-funded, privately-provided surgery.

Comparison to other studies

A previous study[23] using data for England from Hospital Episode Statistics found increasing private provision of elective hip arthroplasties nationally from 2007/8 to 2012/13, particularly for less deprived people, which echoes our findings. More recent news stories have suggested that 20% of NHS-funded hip replacements and 29% of NHS-funded knee replacements were carried out by independent providers in 2016/17[24], and that independently-provided hip and knee replacement surgery (privately or NHS-funded) has now overtaken NHS provision.[25] A UK-wide study[6] using primary care data (CPRD) linked to hospital admissions found similar effects of patient characteristics (age, sex, comorbidity, and deprivation) on length of stay for primary hip and knee replacements, although they did not explore seasonality. A recent qualitative study[26] highlighted the negative financial and emotional impact of winter elective cancellations on patients and their families and recommended better advanced planning of elective operations to reduce these impacts.

Implications for clinicians and policy makers

Outsourcing of less complex hip and knee replacements to take advantage of spare capacity in non-NHS hospitals may be a good strategy to reduce waiting times and waiting lists for surgery and get the best results for patients given the evident capacity limitations. It is a strategy that has already been used in other NHS Trusts,[27] and outsourcing more generally is recognised by the British Medical Association as a short-term solution to reducing waiting lists, although they recommend this goes alongside a longer-term commitment to increased NHS capacity.[28] The evidence is unclear regarding the impact of private provision on quality of care for patients and value for money for the public sector,[29 30] with some studies indicating potentially lower quality of healthcare.[31] There are questions

For peer review only - http://bmjopen.bmj.com/site/about/guidelines.xhtml

about how much it increases staff capacity because some staff transfer from public to private practice.[28 29] It would also leave the NHS Trust to cope with more complex cases,[30] which could have a detrimental impact on their service.

There are training implications of outsourcing because trainee surgeons are usually trained in NHS hospitals by first undertaking less-complex cases on healthier patients. Trainees can find they are redeployed away from training to cover for a lack of trained staff in the public sector, which may be detrimental to their training and potentially harmful for patients.[32 33] The Royal College of Surgeons offers guidance around appropriate redeployment of trainees.[34]

There are also potential equity implications of outsourcing, if less complex cases have the option of surgery with shorter waiting times at independent providers, whilst more complex (and potentially more deprived) cases do not. We would need to consider the acceptability of this outsourcing to patients and practitioners, and the quality of patient outcomes.

There is an indication that some selection of patients for elective surgery depending on available capacity already takes place at the Trust. It is possible that this could become a more explicit strategy, based on evidence, to optimise the use of limited capacity in hospitals at different times of the year. However, this could mean that people placed earlier on the waiting list for surgery might get their surgery later due to such scheduling strategies, so acceptability to patients would need to be explored. We need to understand how the scheduling and possible outsourcing of elective surgery for different types of patients, depending on capacity, may impact on throughput of patients, waiting times, waiting lists, outcomes of surgery, costs, and equity of access to surgery. An appropriate balance would need to be achieved to maximise the benefits for patients, and research is needed to understand what that balance is. Additionally, we need to understand whether this type of scheduling and outsourcing is acceptable to people waiting for hip and knee surgery as well as clinicians. These issues of optimising limited elective resources are in even sharper focus due to the backlog in waiting lists caused by the COVID-19 pandemic.

For peer review only - http://bmjopen.bmj.com/site/about/guidelines.xhtml

Unanswered questions and future research

We need to understand how the scheduling and possible outsourcing of elective surgery for different types of patients, depending on capacity, may impact on throughput of patients, waiting times, waiting lists, outcomes of surgery, costs, and equity of access to surgery. Inevitably outsourcing simpler patients to the independent sector will leave more complex patients being treated by NHS Trusts, which could have a detrimental impact on their service. An appropriate balance would need to be achieved to maximise the benefits for patients, and research is needed to understand what that balance is. Additionally, we need to understand whether this type of scheduling and outsourcing is acceptable to people waiting for hip and knee surgery as well as clinicians.

Conclusions

Declining elective capacity and seasonality has a marked effect on the provision of joint replacement, despite efficiency improvements in hospital treatment. The Trust has outsourced less complex patients to independent providers, and/or treated them during winter when capacity is most limited. There is a need to explore whether these are strategies that could be used explicitly to maximise the use of limited elective capacity, provide benefit to patients, and value for money for taxpayers.

Author Contributions

This publication is the work of the authors, who serve as guarantors for the contents of this paper. TJ contributed to study design, data cleaning, data analysis, interpretation of results and writing the manuscript. MTR contributed to study conceptualisation, supervision, interpretation of results and reviewing the manuscript. TK contributed to data curation, supervision, interpretation of results and reviewing the manuscript. AE contributed to data curation, interpretation of results and reviewing

For peer review only - http://bmjopen.bmj.com/site/about/guidelines.xhtml

the manuscript. CP and EE contributed to interpretation of results and reviewing the manuscript. AB contributed to study conceptualisation, supervision, interpretation of results and reviewing the manuscript. AJ contributed to study conceptualisation and design, supervision, and writing the manuscript. TJ had full access to the data in the study and takes responsibility for the integrity of the data and the accuracy of the data analysis.

Acknowledgements

The study reported here would not have been possible without information provided by the NHS Trust, and the use of their hospital admissions data via the NIHR ARC West Partnership Agreement.

Funding

This research was funded by Health Data Research UK (HDR UK) Better Care South-West Partnership. EE, TJ, and MTR's time was supported by the National Institute for Health Research Applied Research Collaboration West (NIHR ARC West). AJ and CP were supported by the NIHR Biomedical Research Centre at University Hospitals Bristol and Weston NHS Foundation Trust and the University of Bristol. The views expressed in this article are those of the author(s) and not necessarily those of the NHS, the NIHR, the Department of Health and Social Care or HDR UK.

Ethical Approval

Not applicable / No human participants included.

Data Sharing

For peer review only

The data from the NHS Trust was obtained under the NIHR ARC West Partnership Agreement. The agreement precludes us from sharing the raw data but it can be published and shared once aggregated to a non-identifiable level.

The data from Hospital Episode Statistics (HES) was obtained under licence (DARS-NIC-17875-X7K1V) from NHS Digital (previously the Health and Social Care Information Centre); *Copyright © 2022, re-used with the permission of The Health & Social Care Information Centre. All rights reserved*. The data are provided by patients and collected by the NHS as part of their care and support. HES data can be accessed via NHS Digital: https://digital.nhs.uk/services/data-access-request-service-dars

Transparency

The manuscript's guarantor (TJ) affirms that the manuscript is an honest, accurate, and transparent account of the study being reported; that no important aspects of the study have been omitted; and that any discrepancies from the study as originally planned have been explained.

Competing Interests

All authors have completed the ICMJE uniform disclosure form at www.icmje.org/coi_disclosure.pdf and declare: TJ, EE, and TR had financial support from NIHR ARC West for the submitted work; AJ has had financial support in the previous three years through institutional grants from NIHR, HDR UK, Versus Arthritis, Healthcare Quality Improvement Partnership (HQIP), Royal College of Physicians (RCP), and Health Foundation, had unpaid committee or leadership roles relating to musculoskeletal conditions for NIHR, Nuffield Foundation, Warwick CTU, and Versus Arthritis, and a paid expert panel role for Nuffield Foundation Oliver Bird Fund; no other financial relationships with any organisations that might have an interest in the submitted work in the previous three years; no other relationships or activities that could appear to have influenced the submitted work.

For peer review only - http://bmjopen.bmj.com/site/about/guidelines.xhtml

REFERENCES

1. Carr AJ, Robertsson O, Graves S, et al. Knee replacement. *Lancet* 2012;379(9823):1331-40. doi: 10.1016/S0140-6736(11)60752-6
2. Pivec R, Johnson AJ, Mears SC, et al. Hip arthroplasty. *Lancet* 2012;380(9855):1768-77. doi: 10.1016/S0140-6736(12)60607-2
3. Price AJ, Alvand A, Troelsen A, et al. Hip and knee replacement 2 Knee replacement. *Lancet* 2018;392(10158):1672-82. doi: Doi 10.1016/S0140-6736(18)32344-4
4. Ferguson RJ, Palmer AJ, Taylor A, et al. Hip replacement. *Lancet* 2018;392(10158):1662-71. doi: 10.1016/S0140-6736(18)31777-X [published Online First: 2018/11/30]
5. Culliford DJ, Maskell J, Beard DJ, et al. Temporal trends in hip and knee replacement in the United Kingdom 1991 TO 2006. *J Bone Joint Surg Br* 2010;92b(1):130-35. doi: 10.1302/0301-620x.92b1.22654
6. Burn E, Edwards CJ, Murray DW, et al. Trends and determinants of length of stay and hospital reimbursement following knee and hip replacement: evidence from linked primary care and NHS hospital records from 1997 to 2014. *Bmj Open* 2018;8(1) doi: 10.1136/bmjopen-2017-019146
7. Kehlet H. Fast-track hip and knee arthroplasty. *Lancet* 2013;381(9878):1600-02. doi: Doi 10.1016/S0140-6736(13)61003-X
8. Briggs T. A national review of adult elective orthopaedic services in England 2015 [Available from: https://gettingitrightfirsttime.co.uk/wp-content/uploads/2017/06/GIRFT-National-Report-Mar15-Web.pdf.
9. Ward D, Wenzel L. A new trend in elective hip surgery 2019 [Available from: https://www.kingsfund.org.uk/blog/2019/06/new-trend-elective-hip-surgery.
10. Fisher E, Merry L. Winter 2017/18: the worst ever for the NHS? 2018 [Available from: https://www.nuffieldtrust.org.uk/news-item/winter-2017-18-the-worst-ever-for-the-nhs.
11. BMJ. Covid-19: “Life on hold” for NHS patients needing musculoskeletal care 2021 [Available from: https://doi.org/10.1136/bmj.n1616.
12. Bernal JL, Cummins S, Gasparrini A. Interrupted time series regression for the evaluation of public health interventions: a tutorial. *Int J Epidemiol* 2017;46(1):348-55. doi: 10.1093/ije/dyw098
13. Kontopantelis E, Doran T, Springate DA, et al. Regression based quasi-experimental approach when randomisation is not an option: interrupted time series analysis. *Bmj-Brit Med J* 2015;350 doi: 10.1136/bmj.h2750
14. Leatherdale ST. Natural experiment methodology for research: a review of how different methods can support real-world research. *Int J Soc Res Method* 2019;22(1):19-35. doi: 10.1080/13645579.2018.1488449
15. Benchimol EI, Smeeth L, Guttmann A, et al. The REporting of studies Conducted using Observational Routinely-collected health Data (RECORD) Statement. *Plos Med* 2015;12(10) doi: 10.1371/journal.pmed.1001885
16. Herbert A, Wijlaars L, Zylbersztejn A, et al. Data Resource Profile: Hospital Episode Statistics Admitted Patient Care (HES APC). *Int J Epidemiol* 2017;46(4):1093-+. doi: 10.1093/ije/dyx015
17. Quan H, Sundararajan V, Halfon P, et al. Coding algorithms for defining comorbidities in ICD-9-CM and ICD-10 administrative data. *Med Care* 2005;43(11):1130-9. doi: 10.1097/01.mlr.0000182534.19832.83 [published Online First: 2005/10/15]
18. Office for National Statistics. LSOA (2011) to Clinical Commissioning Groups to Sustainability and Transformation Partnerships (April 2021) Lookup in England 2021 [Available from: https://geoportal.statistics.gov.uk/datasets/ons::lsoa-2011-to-clinical-commissioning-

For peer review only - http://bmjopen.bmj.com/site/about/guidelines.xhtml

groups-to-sustainability-and-transformation-partnerships-april-2021-lookup-in-england/explore.

19. Bhaskaran K, Gasparrini A, Hajat S, et al. Time series regression studies in environmental epidemiology. *Int J Epidemiol* 2013;42(4):1187-95. doi: 10.1093/ije/dyt092
20. Bottomley C, Scott JAG, Isham V. Analysing Interrupted Time Series with a Control. *Epidemiologic Methods* 2019;8(1) doi: 10.1515/em-2018-0010
21. Linden A. Combining synthetic controls and interrupted time series analysis to improve causal inference in program evaluation. *J Eval Clin Pract* 2018;24(2):447-53. doi: 10.1111/jep.12882
22. Dorward J, Khubone T, Gate K, et al. The impact of the COVID-19 lockdown on HIV care in 65 South African primary care clinics: an interrupted time series analysis. *Lancet Hiv* 2021;8(3):e158-e65. doi: 10.1016/S2352-3018(20)30359-3
23. Sutaria S, Kirkwood G, Pollock AM. An ecological study of NHS funded elective hip arthroplasties in England from 2003/04 to 2012/13. *J Roy Soc Med* 2019;112(7):292-303. doi: 10.1177/0141076819851701
24. The Independent. NHS privatisation fears after figures show third of knee replacements done by private sector, 2018. Available from: https://www.independent.co.uk/news/uk/home-news/nhs-knee-hip-replacements-private-sector-a8549006.html.
25. Dench J. Private hospital overtakes NHS for hip and knee replacement, 2021. Available from: https://londonnewstime.com/private-hospital-overtakes-nhs-for-hip-and-knee-replacement/479438/.
26. Herrod PJJ, Adiamah A, Boyd-Carson H, et al. Winter cancellations of elective surgical procedures in the UK: a questionnaire survey of patients on the economic and psychological impact. *Bmj Open* 2019;9(9) doi: 10.1136/bmjopen-2018-028753
27. University Hospitals of Leicester NHS Trust. Impact of Emergency Activity on Planned Surgery Leicester, 2019. Available from: https://cabinet.leicester.gov.uk/documents/s98190/HWB%20-%20Emergency%20and%20the%20impact%20on%20Planned%20Surgeries.pdf accessed March 2023.
28. British Medical Association. Outsourced: the role of the independent sector in the NHS, 2022. Available from: https://www.bma.org.uk/media/5378/bma-nhs-outsourcing-report-march-2022.pdf.
29. Pollock AM, Godden S. Independent sector treatment centres: evidence so far. *Bmj-Brit Med J* 2008;336(7641):421-24. doi: DOI 10.1136/bmj.39470.505556.80
30. Cooper Z, Gibbons S, Skellern M. Does competition from private surgical centres improve public hospitals' performance? Evidence from the English National Health Service. *J Public Econ* 2018;166:63-80. doi: 10.1016/j.jpubeco.2018.08.002
31. Goodair B, Reeves A. Outsourcing health-care services to the private sector and treatable mortality rates in England, 2013-20: an observational study of NHS privatisation. *Lancet Public Health* 2022;7(7):E638-E46.
32. Association of Surgeons in Training. Winter Pressures affecting Surgeons in Training, 2018. Available from: https://www.asit.org/resources/archived-articles-documents/2018/winter-pressures-affecting-surgeons-in-training/res1289.
33. British Orthopaedic Association. BOA statement on Training and Winter Pressures, 2018. Available from: https://www.boa.ac.uk/resources/boa-statement-on-training-and-winter-pressures.html.
34. Royal College of Surgeons. Guidance to help manage winter pressures, 2018. Available from: https://www.rcseng.ac.uk/news-and-events/news/archive/guidance-to-help-manage-winter-pressures/.

For peer review only

For peer review only - http://bmjopen.bmj.com/site/about/guidelines.xhtml

FIGURE CAPTIONS

Figure 1. Elective hip (left panel) and knee (right panel) replacement admissions at the Trust
Note: grey area shows the winter 2017 cancellations and is excluded from the analysis

Figure 2. Average age on admission for hip (left panel) and knee (right panel) replacements at the Trust
Note: grey area shows the winter 2017 cancellations and is excluded from the analysis

Figure 3. Proportion of people having hip (left panel) and knee (right panel) replacements with 2+ Charlson comorbidities recorded
Note: grey area shows the winter 2017 cancellations and is excluded from the analysis

Figure 4. Ratio of public to private provision of elective hip (left panel) and knee (right panel) replacements for NHS patients in the Trust CCG
Note: grey area shows the winter 2017 cancellations and is excluded from the analysis

Figure 5. Bed occupancy for hip (left panel) and knee (right panel) replacements at the Trust
Note: grey area shows the winter 2017 cancellations and is excluded from the analysis

For peer review only

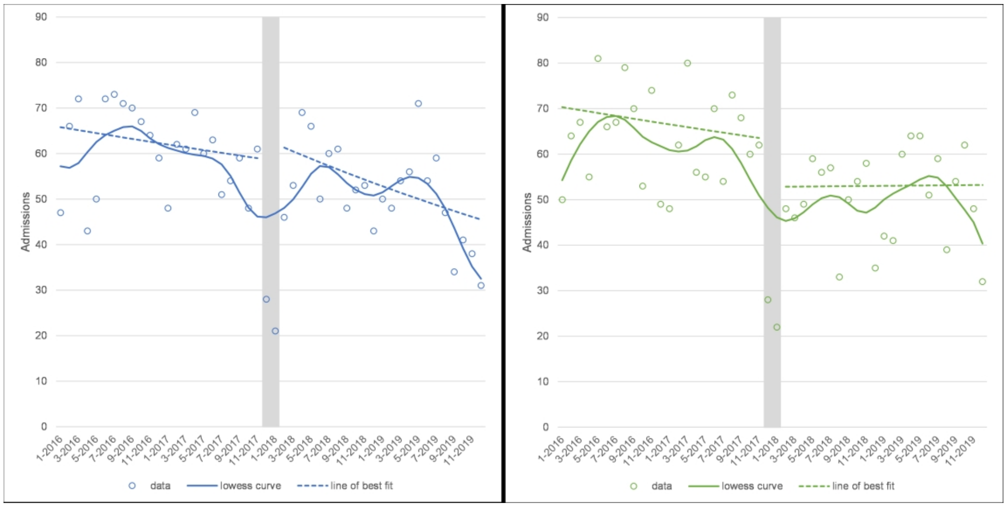

Figure 1. Elective hip (left panel) and knee (right panel) replacement admissions at the Trust
Note: grey area shows the winter 2017 cancellations and is excluded from the analysis

301x150mm (300 x 300 DPI)

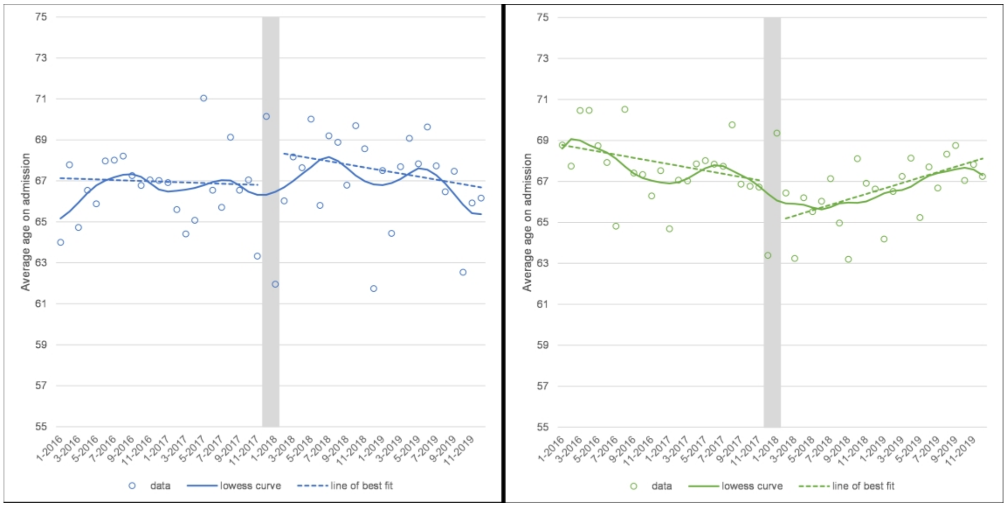

Figure 2. Average age on admission for hip (left panel) and knee (right panel) replacements at the Trust
Note: grey area shows the winter 2017 cancellations and is excluded from the analysis

301x150mm (300 x 300 DPI)

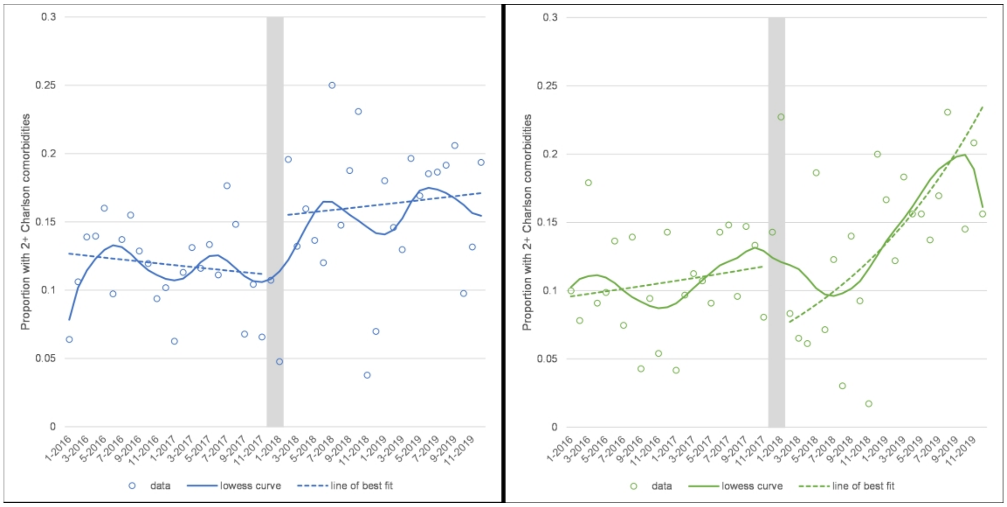

Figure 3. Proportion of people having hip (left panel) and knee (right panel) replacements with 2+ Charlson comorbidities recorded
Note: grey area shows the winter 2017 cancellations and is excluded from the analysis

301x150mm (300 x 300 DPI)

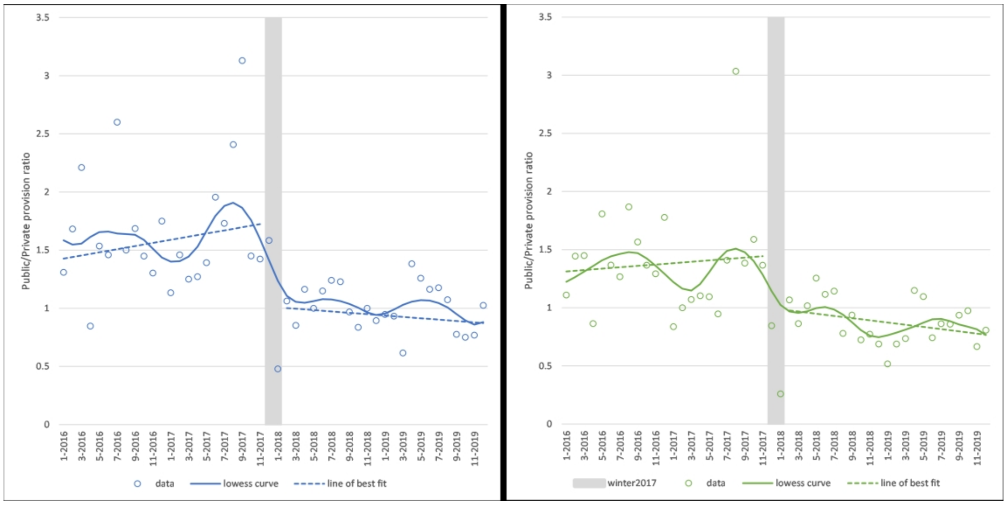

Figure 4. Ratio of public to private provision of elective hip (left panel) and knee (right panel) replacements for NHS patients in the Trust CCG
Note: grey area shows the winter 2017 cancellations and is excluded from the analysis

301x150mm (300 x 300 DPI)

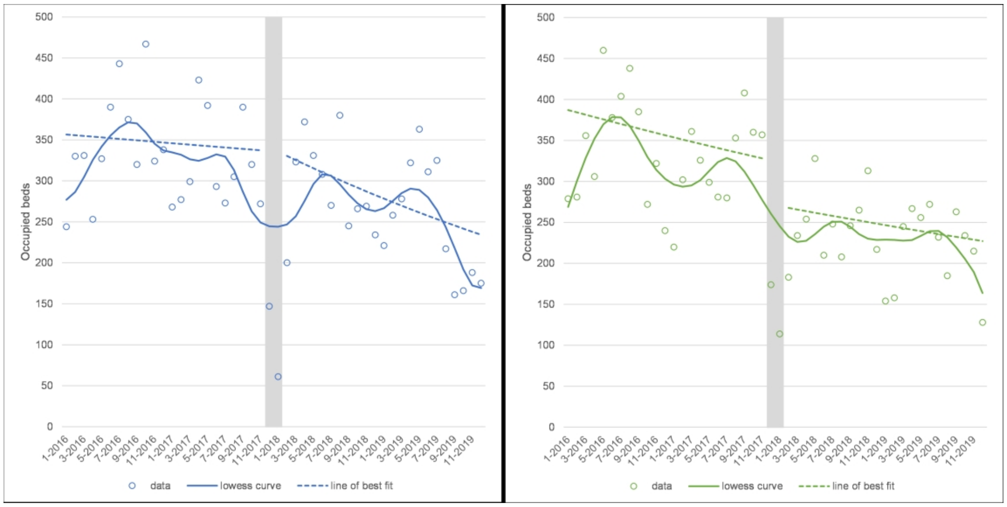

Figure 5. Bed occupancy for hip (left panel) and knee (right panel) replacements at the Trust
Note: grey area shows the winter 2017 cancellations and is excluded from the analysis

301x150mm (300 x 300 DPI)

Supplementary Figure F1. Ratio of elective to emergency hospital admissions for any reason at the Trust

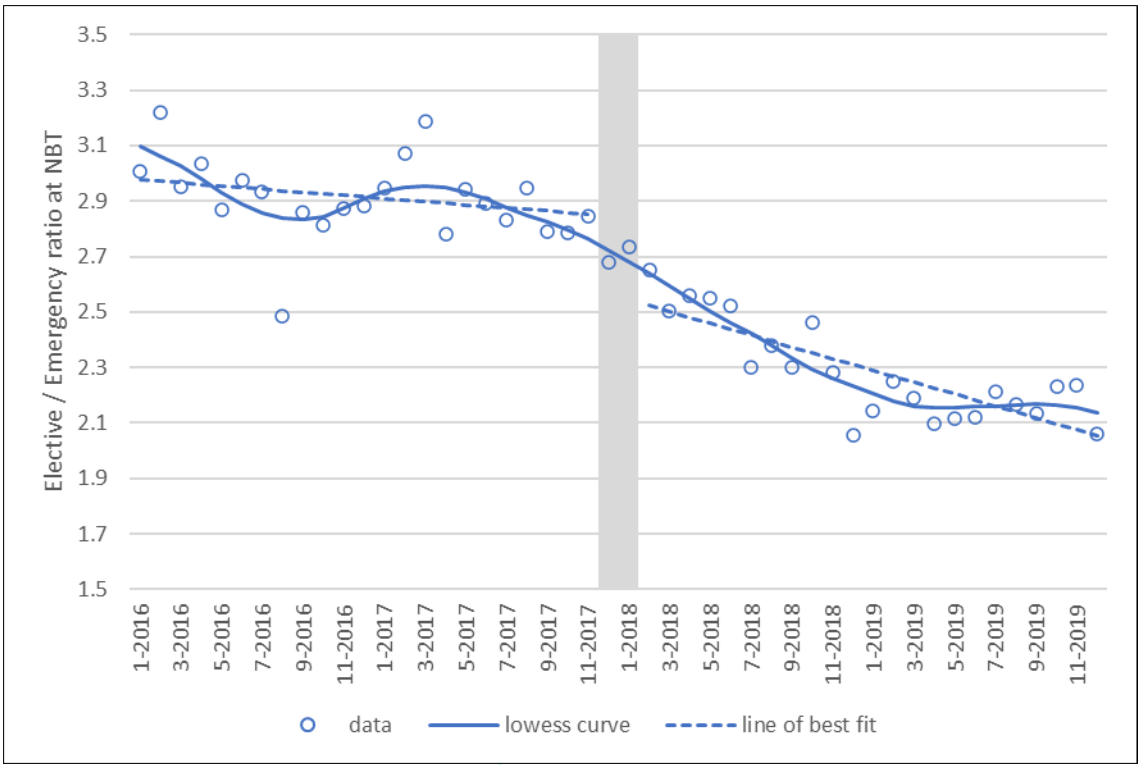

Note: grey area shows the winter 2017 cancellations and is excluded from the analysis

For peer review only

Supplementary Figure F2. Average length of stay for primary hip (left) and knee (right) replacement operations at NBT

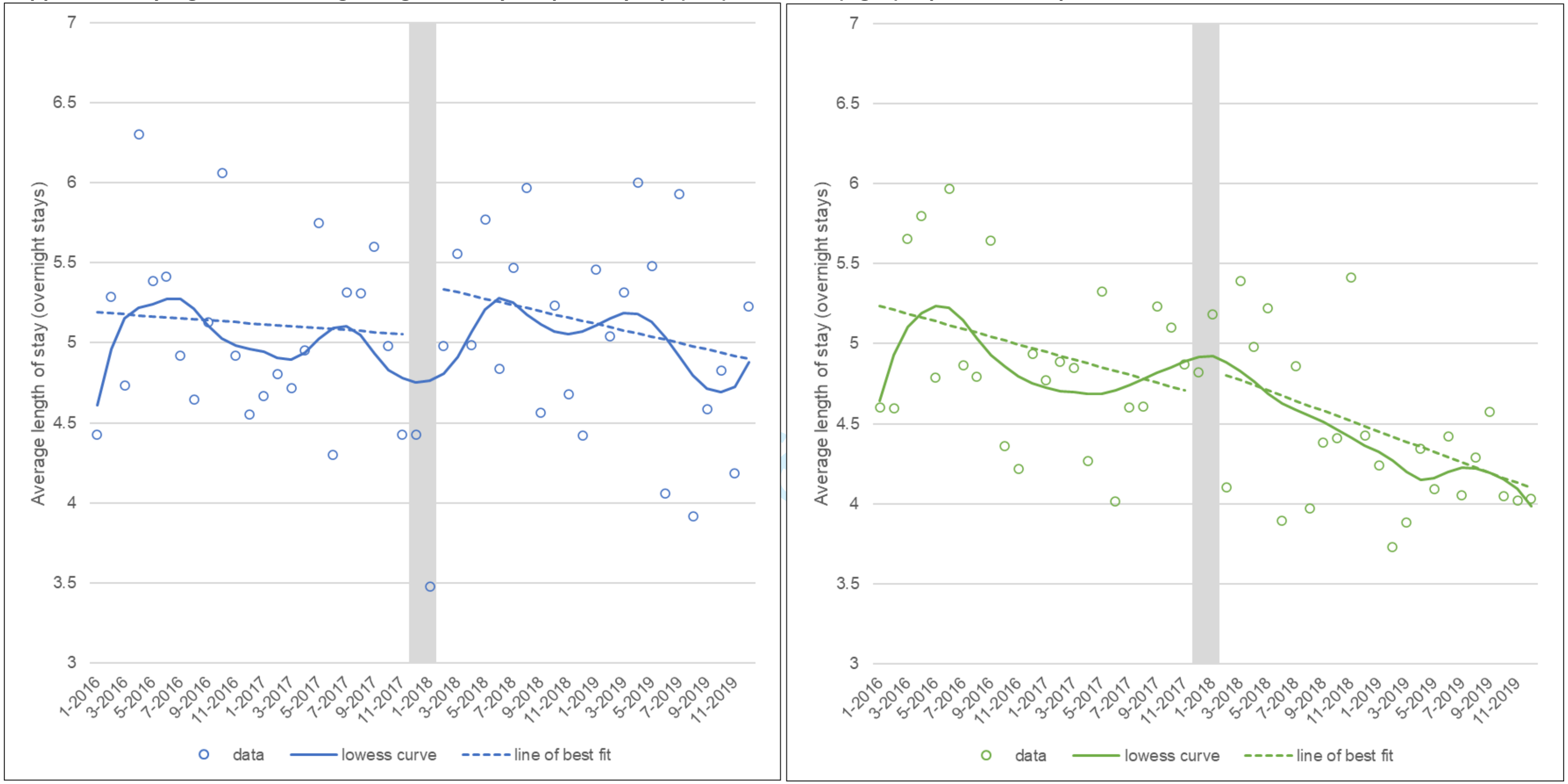

Note: grey area shows the winter 2017 cancellations and is excluded from the analysis

For peer review only

For peer review only - http://bmjopen.bmj.com/site/about/guidelines.xhtml

Supplementary Table T1. OPCS-4 codes used to identify primary hip and knee replacement operations

Category	Code	Description	Notes
Primary Total Hip Replacement	W37.1	Primary total prosthetic replacement of hip joint using cement	
	W37.8	Other specified total prosthetic replacement of hip joint using cement	
	W37.9	Unspecified total prosthetic replacement of hip joint using cement	
	W38.1	Primary total prosthetic replacement of hip joint not using cement	
	W38.8	Other specified total prosthetic replacement of hip joint not using cement	
	W38.9	Unspecified total prosthetic replacement of hip joint not using cement	
	W39.1	Primary total prosthetic replacement of hip joint NEC	
	W39.8	Other specified other total prosthetic replacement of hip joint	
	W39.9	Unspecified other total prosthetic replacement of hip joint	
	W43.1	Primary total prosthetic replacement of other joint using cement NEC	
	W43.8	Other specified total prosthetic replacement of other joint using cement NEC	
	W43.9	Unspecified total prosthetic replacement of other joint using cement NEC	
	W44.1	Primary total prosthetic replacement of other joint not using cement NEC	
	W44.8	Other specified total prosthetic replacement of other joint not using cement NEC	
	W44.9	Unspecified total prosthetic replacement of other joint not using cement NEC	
	W45.1	Other primary total prosthetic replacement of other joint NEC	
	W45.8	Other specified total prosthetic replacement of other joint NEC	
	W45.9	Unspecified total prosthetic replacement of other joint NEC	
	W52.1	Primary prosthetic replacement of articulation of bone using cement NEC	

For peer review only - http://bmjopen.bmj.com/site/about/guidelines.xhtml

W52.8	Other specified prosthetic replacement of articulation of bone using cement NEC
W52.9	Unspecified prosthetic replacement of articulation of bone using cement NEC
W53.1	Primary prosthetic replacement of articulation of bone not using cement NEC
W53.8	Other specified prosthetic replacement of articulation of bone not using cement NEC
W53.9	Unspecified prosthetic replacement of articulation of bone not using cement NEC
W54.1	Primary prosthetic replacement of articulation of bone NEC
W54.8	Other specified prosthetic replacement of articulation of bone NEC
W54.9	Unspecified prosthetic replacement of articulation of bone NEC
W93.1	Primary hybrid prosthetic replacement of hip joint using cemented acetabular component
W93.8	Other specified hybrid prosthetic replacement of hip joint using cemented acetabular component
W93.9	Unspecified hybrid prosthetic replacement of hip joint using cemented acetabular component
W94.1	Primary hybrid prosthetic replacement of hip joint using cemented femoral component
W94.8	Other specified hybrid prosthetic replacement of hip joint using cemented femoral component
W94.9	Unspecified hybrid prosthetic replacement of hip joint using cemented femoral component
W95.1	Primary hybrid prosthetic replacement of hip joint using cement NEC
W95.8	Other specified hybrid prosthetic replacement of hip joint using cement
W95.9	Unspecified hybrid prosthetic replacement of hip joint using cement

For peer review only

Primary Total Knee Replacement	W40.1	Primary total prosthetic replacement of knee joint using cement	
	W40.8	Other specified total prosthetic replacement of knee joint using cement	
	W40.9	Unspecified total prosthetic replacement of knee joint using cement	
	W41.1	Primary total prosthetic replacement of knee joint not using cement	
	W41.8	Other specified total prosthetic replacement of knee joint not using cement	
	W41.9	Unspecified total prosthetic replacement of knee joint not using cement	
	W42.1	Primary total prosthetic replacement of knee joint NEC	
	W42.8	Other specified other total prosthetic replacement of knee joint	
	W42.9	Unspecified other total prosthetic replacement of knee joint	
	O18.1	Primary hybrid prosthetic replacement of knee joint using cement	
	O18.8	Other specified hybrid prosthetic replacement of knee joint using cement	
	O18.9	Unspecified hybrid prosthetic replacement of knee joint using cement	
Resurfacing / Reconstruction	W58.1	Primary resurfacing arthroplasty of joint	Require combination with site + combination codes to ID
	W58.8	Other specified reconstruction of joint	Require combination with site + combination codes to ID
	W58.9	Unspecified other reconstruction of joint	Require combination with site + combination codes to ID
Primary unicondylar / unicompartmental knee operations	W52.1	Primary prosthetic replacement of articulation of bone using cement NEC	Require combination with site + combination codes to ID
	W52.8	Other specified prosthetic replacement of articulation of other bone using cement	Require combination with site + combination codes to ID
	W52.9	Unspecified prosthetic replacement of articulation of other bone using cement	Require combination with site + combination codes to ID
	W53.1	Primary prosthetic replacement of articulation of bone not using cement NEC	Require combination with site + combination codes to ID

For peer review only - http://bmjopen.bmj.com/site/about/guidelines.xhtml

W53.9	Unspecified prosthetic replacement of articulation of other bone not using cement	Require combination with site + combination codes to ID
W54.0	Conversion from previous prosthetic replacement of articulation of bone NEC	Require combination with site + combination codes to ID
W54.1	Primary prosthetic replacement of articulation of bone NEC	Require combination with site + combination codes to ID
W54.8	Other specified other prosthetic replacement of articulation of other bone	Require combination with site + combination codes to ID
W54.9	Unspecified other prosthetic replacement of articulation of other bone	Require combination with site + combination codes to ID

For peer review only

Supplementary Table T2. Interrupted time series model results with maximum auto-correlation lag 2

	pre-trend		level change		trend change		spring		summer		autumn	
	estimate (95% CI)	p	estimate (95% CI)	p	estimate (95% CI)	p	estimate (95% CI)	p	estimate (95% CI)	p	estimate (95% CI)	p
Hip Admissions	0.995 (0.986,1.004)	0.300	1.055 (0.912,1.221)	0.469	0.991 (0.977,1.006)	0.239	1.19 (1.034,1.37)	0.015	1.207 (1.094,1.332)	0.000	1.101 (0.982,1.234)	0.099
Hip Average Age	-0.015 (-0.102,0.072)	0.737	1.571 (-0.1,3.242)	0.065	-0.06 (-0.175,0.055)	0.307	1.52 (-0.07,3.111)	0.061	2.09 (0.811,3.368)	0.001	1.031 (-0.598,2.66)	0.215
Hip Prop Women	0.999 (0.995,1.003)	0.582	0.966 (0.861,1.083)	0.549	1.007 (0.999,1.016)	0.089	1.023 (0.941,1.111)	0.596	1.088 (1.001,1.183)	0.048	0.981 (0.888,1.083)	0.700
Hip Prop 2+ Charlson	0.994 (0.982,1.007)	0.380	1.411 (1.064,1.873)	0.017	1.01 (0.994,1.027)	0.220	1.195 (0.982,1.455)	0.075	1.306 (1.096,1.557)	0.003	1.003 (0.788,1.276)	0.982
Hip Prop High Deprivation	1.003 (0.993,1.013)	0.587	1.027 (0.869,1.214)	0.754	1.004 (0.986,1.022)	0.660	0.937 (0.815,1.076)	0.358	0.877 (0.752,1.022)	0.093	0.996 (0.84,1.18)	0.963
Hip LoS	-0.006 (-0.034,0.021)	0.660	0.312 (-0.193,0.818)	0.225	-0.014 (-0.047,0.02)	0.425	0.502 (0.214,0.79)	0.001	0.135 (-0.194,0.463)	0.422	0.1 (-0.288,0.488)	0.613
Hip LoS Age 16-59	-0.003 (-0.028,0.021)	0.788	0.379 (-0.094,0.851)	0.116	-0.026 (-0.065,0.013)	0.190	0.223 (-0.156,0.602)	0.249	0.384 (0.065,0.703)	0.018	0.219 (-0.183,0.622)	0.285
Hip LoS Age 60-69	-0.004 (-0.034,0.026)	0.818	0.013 (-0.807,0.832)	0.976	0 (-0.053,0.052)	0.988	0.107 (-0.44,0.654)	0.702	0.158 (-0.468,0.783)	0.621	0.162 (-0.487,0.811)	0.625
Hip LoS Age 70-79	0.006 (-0.058,0.069)	0.862	-0.753 (-2.003,0.497)	0.238	0.007 (-0.06,0.073)	0.847	-0.433 (-1.416,0.549)	0.387	-0.842 (-1.93,0.246)	0.129	-0.865 (-1.991,0.262)	0.132
Hip LoS Age 80+	-0.068 (-0.171,0.036)	0.199	2.109 (0.683,3.535)	0.004	-0.002 (-0.128,0.123)	0.971	1.506 (0.515,2.497)	0.003	0.222 (-0.773,1.217)	0.662	1.003 (-0.229,2.235)	0.111
Hip LoS Men	-0.007 (-0.055,0.041)	0.776	0.347 (-0.583,1.278)	0.464	-0.02 (-0.083,0.043)	0.536	0.293 (-0.126,0.712)	0.170	0.289 (-0.196,0.774)	0.243	0.458 (-0.111,1.026)	0.114
Hip LoS Women	-0.004 (-0.041,0.033)	0.842	0.235 (-0.308,0.778)	0.396	-0.014 (-0.055,0.027)	0.505	0.693 (0.309,1.077)	0.000	0.009 (-0.428,0.446)	0.966	-0.203 (-0.728,0.323)	0.449
Hip LoS Charlson 0	-0.011 (-0.042,0.019)	0.469	0.297 (-0.288,0.882)	0.319	-0.005 (-0.042,0.031)	0.782	0.912 (0.549,1.276)	0.000	0.178 (-0.148,0.503)	0.285	0.222 (-0.065,0.509)	0.129
Hip LoS Charlson 1	0.034 (-0.012,0.08)	0.146	0.238 (-0.709,1.184)	0.623	-0.077 (-0.145,-0.01)	0.024	0.191 (-0.418,0.801)	0.538	0.312 (-0.405,1.029)	0.393	0.014 (-0.578,0.606)	0.963
Hip LoS Charlson 2+	-0.123 (-0.259,0.013)	0.077	0.172 (-2.224,2.569)	0.888	0.116 (-0.016,0.249)	0.085	-0.789 (-2.141,0.563)	0.253	-0.783 (-2.087,0.521)	0.239	0.188 (-2.051,2.427)	0.869
Hip LoS Dep 1	0.004 (-0.032,0.04)	0.829	0.553 (-0.391,1.496)	0.251	-0.017 (-0.068,0.035)	0.529	0.784 (0.107,1.462)	0.023	0.42 (-0.024,0.863)	0.064	0.299 (-0.358,0.956)	0.373
Hip LoS Dep 2	-0.038 (-0.083,0.006)	0.092	0.822 (-0.392,2.035)	0.184	-0.015 (-0.086,0.055)	0.670	1.509 (0.759,2.258)	0.000	0.66 (-0.246,1.567)	0.154	0.589 (-0.417,1.594)	0.251
Hip LoS Dep 3	-0.034 (-0.107,0.038)	0.353	0.617 (-0.897,2.131)	0.424	0.01 (-0.076,0.095)	0.827	-0.216 (-1.224,0.792)	0.675	-0.424 (-1.378,0.53)	0.384	-0.701 (-1.536,0.135)	0.100
Hip LoS Dep 4	0.081 (-0.005,0.166)	0.064	-0.473 (-2.181,1.236)	0.588	-0.112 (-0.215,-0.009)	0.034	-0.374 (-1.65,0.903)	0.566	-1.204 (-2.351,-0.057)	0.040	-0.491 (-1.918,0.935)	0.500
Hip LoS Dep 5	0.015 (-0.086,0.116)	0.773	-0.72 (-2.059,0.62)	0.292	0 (-0.098,0.099)	0.999	0.621 (-0.084,1.327)	0.084	0.689 (-0.31,1.689)	0.176	0.816 (-0.784,2.416)	0.317
Hip Bed Occ	0.997 (0.987,1.008)	0.643	1 (0.844,1.186)	0.997	0.987 (0.97,1.005)	0.149	1.291 (1.11,1.501)	0.001	1.283 (1.125,1.463)	0.000	1.146 (0.988,1.328)	0.071
Hip Public Private	0.013 (-0.015,0.041)	0.377	-0.741 (-1.237,-0.245)	0.003	-0.019 (-0.05,0.011)	0.218	-0.008 (-0.212,0.196)	0.939	0.308 (0.154,0.463)	0.000	0.038 (-0.166,0.241)	0.718
Knee Admissions	0.995 (0.99,1.001)	0.106	0.843 (0.728,0.976)	0.022	1.005 (0.996,1.014)	0.256	1.308 (1.157,1.479)	0.000	1.26 (1.138,1.396)	0.000	1.286 (1.164,1.42)	0.000
Knee Average Age	-0.078 (-0.157,0.001)	0.054	-1.632 (-2.988,-0.276)	0.018	0.211 (0.117,0.305)	0.000	0.926 (-0.112,1.965)	0.080	0.953 (-0.021,1.927)	0.055	0.354 (-0.531,1.24)	0.433
Knee Prop Women	1.004 (0.998,1.01)	0.150	0.96 (0.85,1.084)	0.513	0.994 (0.986,1.003)	0.193	1.037 (0.963,1.117)	0.336	1.017 (0.924,1.118)	0.735	1.036 (0.958,1.12)	0.375
Knee Prop 2+ Charlson	1.009 (0.993,1.026)	0.249	0.638 (0.455,0.894)	0.009	1.042 (1.017,1.067)	0.001	1.156 (0.911,1.467)	0.234	1.074 (0.849,1.359)	0.551	0.909 (0.628,1.315)	0.612
Knee Prop High Deprivation	1.005 (0.997,1.013)	0.189	0.968 (0.786,1.191)	0.758	0.986 (0.974,0.998)	0.021	1.224 (1.077,1.39)	0.002	1.075 (0.942,1.227)	0.282	1.031 (0.902,1.178)	0.656
Knee LoS	-0.024 (-0.049,0.001)	0.058	0.176 (-0.279,0.63)	0.449	-0.008 (-0.036,0.02)	0.566	0.422 (0.073,0.771)	0.018	0.15 (-0.166,0.467)	0.352	0.396 (0.015,0.777)	0.042

For peer review only - http://bmjopen.bmj.com/site/about/guidelines.xhtml

Knee LoS Age 16-59	-0.016 (-0.037,0.006)	0.151	0.403 (-0.045,0.852)	0.078	-0.028 (-0.06,0.005)	0.096	0.484 (0.13,0.839)	0.007	0.422 (-0.021,0.866)	0.062	0.475 (0.19,0.76)	0.001
Knee LoS Age 16-59	0.007 (-0.028,0.043)	0.684	-0.295 (-1.015,0.426)	0.423	-0.026 (-0.072,0.02)	0.262	0.069 (-0.46,0.599)	0.797	-0.194 (-0.687,0.3)	0.441	-0.096 (-0.725,0.532)	0.764
Knee LoS Age 70-79	-0.009 (-0.057,0.038)	0.705	0.77 (0.002,1.538)	0.049	-0.054 (-0.11,0.003)	0.061	-0.086 (-0.715,0.542)	0.787	-0.552 (-1.163,0.059)	0.077	0.367 (-0.352,1.085)	0.317
Knee LoS Age 80+	-0.051 (-0.118,0.015)	0.131	-0.562 (-2.298,1.173)	0.525	0.028 (-0.081,0.137)	0.612	1.521 (0.03,3.011)	0.046	1.37 (0.169,2.57)	0.025	1.361 (0.496,2.227)	0.002
Knee LoS Men	-0.007 (-0.031,0.018)	0.581	0.096 (-0.405,0.597)	0.708	-0.033 (-0.068,0.002)	0.064	0.082 (-0.367,0.531)	0.720	0.157 (-0.262,0.575)	0.464	0.205 (-0.221,0.631)	0.346
Knee LoS Women	-0.042 (-0.084,0)	0.052	0.252 (-0.461,0.966)	0.488	0.017 (-0.029,0.063)	0.478	0.635 (0.048,1.223)	0.034	0.15 (-0.397,0.698)	0.590	0.49 (-0.17,1.151)	0.146
Knee LoS Charlson 0	-0.021 (-0.046,0.004)	0.095	0.067 (-0.362,0.495)	0.760	-0.011 (-0.045,0.024)	0.545	0.645 (0.334,0.956)	0.000	0.193 (-0.111,0.497)	0.213	0.415 (0.069,0.76)	0.019
Knee LoS Charlson 1	-0.03 (-0.074,0.015)	0.191	0.47 (-0.391,1.331)	0.284	-0.029 (-0.083,0.025)	0.298	0.455 (-0.043,0.953)	0.073	0.324 (-0.297,0.945)	0.307	1.058 (0.367,1.748)	0.003
Knee LoS Charlson 2+	-0.04 (-0.107,0.028)	0.247	0.407 (-2.28,3.095)	0.766	-0.022 (-0.196,0.151)	0.802	-0.627 (-1.698,0.443)	0.251	-0.949 (-2.342,0.444)	0.182	-1.433 (-2.229,-0.637)	0.000
Knee LoS Dep 1	-0.009 (-0.06,0.041)	0.712	-0.035 (-0.726,0.656)	0.920	-0.008 (-0.073,0.056)	0.800	0.257 (-0.47,0.985)	0.488	0.096 (-0.619,0.811)	0.793	-0.127 (-0.725,0.472)	0.678
Knee LoS Dep 2	-0.019 (-0.06,0.022)	0.358	0.199 (-0.699,1.097)	0.664	-0.043 (-0.098,0.012)	0.124	0.018 (-0.776,0.812)	0.965	-0.731 (-1.49,0.029)	0.059	0.05 (-0.822,0.922)	0.911
Knee LoS Dep 3	0.014 (-0.036,0.064)	0.586	-0.426 (-1.355,0.503)	0.369	-0.036 (-0.106,0.033)	0.302	0.976 (0.061,1.892)	0.036	0.909 (-0.034,1.852)	0.059	0.547 (-0.217,1.312)	0.160
Knee LoS Dep 4	-0.064 (-0.123,-0.004)	0.035	0.634 (-0.293,1.56)	0.180	0.033 (-0.039,0.105)	0.373	0.669 (-0.187,1.525)	0.126	0.617 (0.02,1.214)	0.043	0.852 (-0.208,1.912)	0.115
Knee LoS Dep 5	-0.035 (-0.09,0.021)	0.224	0.709 (-0.303,1.721)	0.170	0.015 (-0.046,0.076)	0.628	0.123 (-0.73,0.976)	0.778	-0.387 (-1.281,0.507)	0.397	1.104 (0.077,2.131)	0.035
Knee Bed Occ	0.993 (0.984,1.002)	0.103	0.834 (0.704,0.989)	0.037	1 (0.989,1.011)	0.993	1.42 (1.297,1.556)	0.000	1.373 (1.218,1.547)	0.000	1.465 (1.312,1.635)	0.000
Knee Public Private	0.006 (-0.02,0.031)	0.667	-0.476 (-1.026,0.074)	0.090	-0.015 (-0.04,0.009)	0.225	0.113 (-0.089,0.314)	0.274	0.276 (0.035,0.517)	0.025	0.131 (-0.071,0.332)	0.205
Elec Emerg Ratio	-0.005 (-0.013,0.002)	0.171	-0.322 (-0.446,-0.198)	0.000	-0.016 (-0.026,-0.005)	0.003	-0.008 (-0.12,0.103)	0.886	-0.053 (-0.182,0.077)	0.424	-0.028 (-0.151,0.096)	0.661

For peer review only

For peer review only - http://bmjopen.bmj.com/site/about/guidelines.xhtml

Supplementary Table T3. Interrupted time series model results with maximum auto-correlation lag 0

	pre-trend		level change		trend change		spring		summer		autumn	
	estimate (95% CI)	p	estimate (95% CI)	p	estimate (95% CI)	p	estimate (95% CI)	p	estimate (95% CI)	p	estimate (95% CI)	p
Hip Admissions	0.995 (0.985,1.005)	0.323	1.055 (0.902,1.235)	0.503	0.991 (0.978,1.005)	0.220	1.19 (1.036,1.367)	0.014	1.207 (1.088,1.339)	0.000	1.101 (0.983,1.234)	0.098
Hip Average Age	-0.015 (-0.134,0.104)	0.806	1.571 (-0.863,4.004)	0.206	-0.06 (-0.208,0.088)	0.427	1.52 (-0.079,3.119)	0.062	2.09 (0.685,3.494)	0.004	1.031 (-0.505,2.567)	0.188
Hip Prop Women	0.999 (0.993,1.004)	0.674	0.966 (0.826,1.13)	0.662	1.007 (0.997,1.017)	0.158	1.023 (0.937,1.116)	0.617	1.088 (0.995,1.189)	0.064	0.981 (0.878,1.096)	0.730
Hip Prop 2+ Charlson	0.994 (0.98,1.009)	0.455	1.411 (0.969,2.055)	0.072	1.01 (0.989,1.031)	0.344	1.195 (0.971,1.471)	0.092	1.306 (1.043,1.637)	0.020	1.003 (0.734,1.37)	0.986
Hip Prop High Deprivation	1.003 (0.991,1.014)	0.631	1.027 (0.846,1.247)	0.787	1.004 (0.983,1.025)	0.704	0.937 (0.819,1.072)	0.342	0.877 (0.746,1.03)	0.110	0.996 (0.831,1.193)	0.965
Hip LoS	-0.006 (-0.038,0.026)	0.704	0.312 (-0.262,0.887)	0.287	-0.014 (-0.058,0.03)	0.542	0.502 (0.132,0.872)	0.008	0.135 (-0.292,0.562)	0.537	0.1 (-0.287,0.487)	0.612
Hip LoS Age 16-59	-0.003 (-0.033,0.026)	0.821	0.379 (-0.165,0.922)	0.172	-0.026 (-0.067,0.015)	0.210	0.223 (-0.17,0.617)	0.266	0.384 (0.053,0.715)	0.023	0.219 (-0.168,0.607)	0.267
Hip LoS Age 60-69	-0.004 (-0.046,0.039)	0.871	0.013 (-1.026,1.052)	0.981	0 (-0.069,0.068)	0.990	0.107 (-0.592,0.805)	0.765	0.158 (-0.514,0.829)	0.645	0.162 (-0.588,0.912)	0.672
Hip LoS Age 70-79	0.006 (-0.067,0.078)	0.879	-0.753 (-2.031,0.525)	0.248	0.007 (-0.069,0.082)	0.865	-0.433 (-1.583,0.716)	0.460	-0.842 (-2.028,0.344)	0.164	-0.865 (-2.164,0.435)	0.192
Hip LoS Age 80+	-0.068 (-0.193,0.058)	0.291	2.109 (0.536,3.682)	0.009	-0.002 (-0.16,0.155)	0.977	1.506 (-0.007,3.018)	0.051	0.222 (-1.036,1.48)	0.729	1.003 (-0.375,2.38)	0.154
Hip LoS Men	-0.007 (-0.056,0.042)	0.783	0.347 (-0.58,1.275)	0.463	-0.02 (-0.084,0.044)	0.543	0.293 (-0.283,0.87)	0.319	0.289 (-0.314,0.892)	0.347	0.458 (-0.15,1.066)	0.140
Hip LoS Women	-0.004 (-0.048,0.041)	0.868	0.235 (-0.563,1.033)	0.564	-0.014 (-0.071,0.043)	0.634	0.693 (0.164,1.222)	0.010	0.009 (-0.543,0.562)	0.973	-0.203 (-0.727,0.322)	0.448
Hip LoS Charlson 0	-0.011 (-0.047,0.024)	0.532	0.297 (-0.428,1.023)	0.422	-0.005 (-0.049,0.039)	0.817	0.912 (0.522,1.302)	0.000	0.178 (-0.232,0.587)	0.396	0.222 (-0.135,0.579)	0.223
Hip LoS Charlson 1	0.034 (-0.023,0.091)	0.240	0.238 (-0.704,1.179)	0.621	-0.077 (-0.157,0.002)	0.056	0.191 (-0.562,0.945)	0.619	0.312 (-0.397,1.022)	0.388	0.014 (-0.544,0.572)	0.960
Hip LoS Charlson 2+	-0.123 (-0.266,0.02)	0.092	0.172 (-2.155,2.5)	0.885	0.116 (-0.04,0.273)	0.144	-0.789 (-2.218,0.64)	0.279	-0.783 (-2.108,0.541)	0.246	0.188 (-1.779,2.156)	0.851
Hip LoS Dep 1	0.004 (-0.039,0.047)	0.857	0.553 (-0.509,1.614)	0.307	-0.017 (-0.078,0.045)	0.599	0.784 (0.118,1.451)	0.021	0.42 (-0.084,0.923)	0.102	0.299 (-0.356,0.954)	0.371
Hip LoS Dep 2	-0.038 (-0.087,0.01)	0.124	0.822 (-0.528,2.172)	0.233	-0.015 (-0.103,0.072)	0.730	1.509 (0.797,2.22)	0.000	0.66 (-0.225,1.546)	0.144	0.589 (-0.26,1.437)	0.174
Hip LoS Dep 3	-0.034 (-0.096,0.027)	0.275	0.617 (-1.066,2.3)	0.472	0.01 (-0.09,0.109)	0.851	-0.216 (-1.343,0.912)	0.708	-0.424 (-1.475,0.627)	0.429	-0.701 (-1.667,0.266)	0.155
Hip LoS Dep 4	0.081 (-0.023,0.185)	0.129	-0.473 (-2.643,1.698)	0.670	-0.112 (-0.258,0.034)	0.134	-0.374 (-1.695,0.947)	0.579	-1.204 (-2.409,0.001)	0.050	-0.491 (-1.978,0.995)	0.517
Hip LoS Dep 5	0.015 (-0.112,0.142)	0.819	-0.72 (-2.66,1.221)	0.467	0 (-0.135,0.135)	0.999	0.621 (-0.412,1.655)	0.239	0.689 (-0.485,1.864)	0.250	0.816 (-0.891,2.524)	0.349
Hip Bed Occ	0.997 (0.987,1.009)	0.653	1 (0.824,1.215)	0.997	0.987 (0.972,1.002)	0.097	1.291 (1.125,1.48)	0.000	1.283 (1.129,1.458)	0.000	1.146 (0.984,1.334)	0.080
Hip Public Private	0.013 (-0.015,0.041)	0.377	-0.741 (-1.237,-0.245)	0.003	-0.019 (-0.05,0.011)	0.218	-0.008 (-0.212,0.196)	0.939	0.308 (0.154,0.463)	0.000	0.038 (-0.166,0.241)	0.718
Knee Admissions	0.995 (0.987,1.004)	0.274	0.843 (0.702,1.013)	0.068	1.005 (0.993,1.017)	0.422	1.308 (1.154,1.483)	0.000	1.26 (1.114,1.426)	0.000	1.286 (1.147,1.441)	0.000
Knee Average Age	-0.078 (-0.157,0.001)	0.053	-1.632 (-3.299,0.035)	0.055	0.211 (0.102,0.32)	0.000	0.926 (-0.129,1.981)	0.085	0.953 (-0.257,2.163)	0.123	0.354 (-0.777,1.485)	0.540
Knee Prop Women	1.004 (0.998,1.011)	0.182	0.96 (0.84,1.097)	0.551	0.994 (0.986,1.003)	0.184	1.037 (0.939,1.146)	0.474	1.017 (0.908,1.138)	0.776	1.036 (0.928,1.157)	0.529
Knee Prop 2+ Charlson	1.009 (0.988,1.031)	0.384	0.638 (0.392,1.037)	0.070	1.042 (1.008,1.077)	0.015	1.156 (0.891,1.501)	0.276	1.074 (0.829,1.393)	0.589	0.909 (0.636,1.299)	0.600
Knee Prop High Deprivation	1.005 (0.997,1.014)	0.237	0.968 (0.784,1.195)	0.761	0.986 (0.974,0.998)	0.023	1.224 (1.099,1.363)	0.000	1.075 (0.956,1.209)	0.225	1.031 (0.911,1.166)	0.631
Knee LoS	-0.024 (-0.052,0.004)	0.089	0.176 (-0.352,0.703)	0.515	-0.008 (-0.041,0.025)	0.625	0.422 (0.117,0.726)	0.007	0.15 (-0.174,0.475)	0.364	0.396 (0.027,0.765)	0.036

Knee LoS Age 16-59	-0.016 (-0.048,0.017)	0.352	0.403 (-0.286,1.093)	0.252	-0.028 (-0.074,0.019)	0.246	0.484 (0.111,0.857)	0.011	0.422 (-0.111,0.956)	0.121	0.475 (0.056,0.893)	0.026
Knee LoS Age 16-59	0.007 (-0.026,0.041)	0.664	-0.295 (-1.22,0.63)	0.532	-0.026 (-0.085,0.033)	0.384	0.069 (-0.465,0.604)	0.799	-0.194 (-0.754,0.366)	0.497	-0.096 (-0.673,0.48)	0.743
Knee LoS Age 70-79	-0.009 (-0.072,0.054)	0.775	0.77 (-0.267,1.807)	0.145	-0.054 (-0.128,0.02)	0.154	-0.086 (-0.782,0.609)	0.808	-0.552 (-1.267,0.163)	0.130	0.367 (-0.528,1.261)	0.421
Knee LoS Age 80+	-0.051 (-0.154,0.051)	0.327	-0.562 (-2.568,1.443)	0.583	0.028 (-0.105,0.161)	0.678	1.521 (-0.076,3.118)	0.062	1.37 (-0.178,2.918)	0.083	1.361 (-0.071,2.794)	0.063
Knee LoS Men	-0.007 (-0.037,0.023)	0.656	0.096 (-0.512,0.704)	0.758	-0.033 (-0.075,0.009)	0.121	0.082 (-0.382,0.547)	0.729	0.157 (-0.272,0.585)	0.474	0.205 (-0.265,0.675)	0.392
Knee LoS Women	-0.042 (-0.088,0.004)	0.071	0.252 (-0.495,1)	0.508	0.017 (-0.036,0.07)	0.536	0.635 (0.107,1.164)	0.019	0.15 (-0.365,0.666)	0.567	0.49 (-0.022,1.003)	0.061
Knee LoS Charlson 0	-0.021 (-0.054,0.012)	0.214	0.067 (-0.49,0.624)	0.814	-0.011 (-0.056,0.034)	0.643	0.645 (0.278,1.013)	0.001	0.193 (-0.146,0.533)	0.265	0.415 (-0.078,0.907)	0.099
Knee LoS Charlson 1	-0.03 (-0.081,0.022)	0.260	0.47 (-0.741,1.681)	0.447	-0.029 (-0.093,0.036)	0.384	0.455 (-0.111,1.021)	0.115	0.324 (-0.426,1.074)	0.398	1.058 (0.262,1.854)	0.009
Knee LoS Charlson 2+	-0.04 (-0.111,0.032)	0.276	0.407 (-2.137,2.951)	0.754	-0.022 (-0.184,0.14)	0.788	-0.627 (-1.883,0.628)	0.327	-0.949 (-2.145,0.247)	0.120	-1.433 (-2.278,-0.589)	0.001
Knee LoS Dep 1	-0.009 (-0.064,0.045)	0.733	-0.035 (-0.918,0.847)	0.937	-0.008 (-0.082,0.066)	0.826	0.257 (-0.443,0.958)	0.471	0.096 (-0.699,0.89)	0.813	-0.127 (-0.786,0.532)	0.706
Knee LoS Dep 2	-0.019 (-0.065,0.026)	0.413	0.199 (-1.031,1.429)	0.751	-0.043 (-0.116,0.031)	0.253	0.018 (-0.822,0.858)	0.967	-0.731 (-1.538,0.077)	0.076	0.05 (-0.819,0.919)	0.910
Knee LoS Dep 3	0.014 (-0.05,0.078)	0.670	-0.426 (-1.892,1.04)	0.569	-0.036 (-0.116,0.043)	0.369	0.976 (-0.09,2.043)	0.073	0.909 (-0.134,1.952)	0.088	0.547 (-0.408,1.503)	0.261
Knee LoS Dep 4	-0.064 (-0.143,0.015)	0.113	0.634 (-0.591,1.858)	0.310	0.033 (-0.057,0.123)	0.474	0.669 (-0.193,1.531)	0.128	0.617 (-0.032,1.266)	0.063	0.852 (-0.138,1.842)	0.092
Knee LoS Dep 5	-0.035 (-0.116,0.047)	0.405	0.709 (-0.701,2.119)	0.324	0.015 (-0.076,0.106)	0.745	0.123 (-0.882,1.128)	0.810	-0.387 (-1.379,0.606)	0.445	1.104 (-0.268,2.476)	0.115
Knee Bed Occ	0.993 (0.984,1.001)	0.074	0.834 (0.711,0.979)	0.027	1 (0.989,1.011)	0.993	1.42 (1.269,1.59)	0.000	1.373 (1.217,1.548)	0.000	1.465 (1.287,1.667)	0.000
Knee Public Private	0.006 (-0.02,0.031)	0.667	-0.476 (-1.026,0.074)	0.090	-0.015 (-0.04,0.009)	0.225	0.113 (-0.089,0.314)	0.274	0.276 (0.035,0.517)	0.025	0.131 (-0.071,0.332)	0.205
Elec Emerg Ratio	-0.005 (-0.013,0.002)	0.167	-0.322 (-0.451,-0.192)	0.000	-0.016 (-0.025,-0.006)	0.001	-0.008 (-0.117,0.101)	0.884	-0.053 (-0.174,0.068)	0.392	-0.028 (-0.128,0.073)	0.591

For peer review only

Supplementary Table T4. Interrupted time series model results with maximum auto-correlation lag 5

	pre-trend		level change		trend change		spring		summer		autumn	
	estimate (95% CI)	p	estimate (95% CI)	p	estimate (95% CI)	p	estimate (95% CI)	p	estimate (95% CI)	p	estimate (95% CI)	p
Hip Admissions	0.995 (0.987,1.003)	0.249	1.055 (0.917,1.215)	0.454	0.991 (0.978,1.005)	0.217	1.19 (1.04,1.362)	0.012	1.207 (1.104,1.32)	0.000	1.101 (0.993,1.221)	0.069
Hip Average Age	-0.015 (-0.094,0.065)	0.714	1.571 (0.241,2.901)	0.021	-0.06 (-0.168,0.048)	0.277	1.52 (-0.178,3.218)	0.079	2.09 (0.818,3.361)	0.001	1.031 (-0.639,2.701)	0.226
Hip Prop Women	0.999 (0.995,1.002)	0.503	0.966 (0.895,1.042)	0.367	1.007 (1.001,1.014)	0.027	1.023 (0.932,1.122)	0.636	1.088 (1,1.184)	0.051	0.981 (0.879,1.094)	0.727
Hip Prop 2+ Charlson	0.994 (0.985,1.004)	0.261	1.411 (1.116,1.785)	0.004	1.01 (1,1.02)	0.042	1.195 (0.979,1.459)	0.079	1.306 (1.111,1.535)	0.001	1.003 (0.792,1.27)	0.981
Hip Prop High Deprivation	1.003 (0.994,1.012)	0.551	1.027 (0.883,1.195)	0.729	1.004 (0.99,1.018)	0.577	0.937 (0.805,1.091)	0.400	0.877 (0.752,1.022)	0.092	0.996 (0.847,1.171)	0.961
Hip LoS	-0.006 (-0.029,0.017)	0.605	0.312 (-0.12,0.745)	0.157	-0.014 (-0.042,0.015)	0.346	0.502 (0.286,0.718)	0.000	0.135 (-0.191,0.461)	0.418	0.1 (-0.332,0.532)	0.650
Hip LoS Age 16-59	-0.003 (-0.026,0.02)	0.773	0.379 (-0.046,0.803)	0.080	-0.026 (-0.062,0.01)	0.152	0.223 (-0.113,0.559)	0.193	0.384 (0.09,0.679)	0.011	0.219 (-0.191,0.63)	0.295
Hip LoS Age 60-69	-0.004 (-0.031,0.024)	0.799	0.013 (-0.766,0.791)	0.975	0 (-0.051,0.05)	0.987	0.107 (-0.33,0.543)	0.632	0.158 (-0.451,0.766)	0.611	0.162 (-0.352,0.676)	0.537
Hip LoS Age 70-79	0.006 (-0.054,0.065)	0.853	-0.753 (-1.944,0.438)	0.215	0.007 (-0.056,0.069)	0.836	-0.433 (-1.343,0.476)	0.350	-0.842 (-1.91,0.226)	0.122	-0.865 (-2.063,0.334)	0.157
Hip LoS Age 80+	-0.068 (-0.147,0.012)	0.097	2.109 (0.803,3.414)	0.002	-0.002 (-0.111,0.107)	0.967	1.506 (0.499,2.513)	0.003	0.222 (-0.799,1.243)	0.670	1.003 (-0.111,2.116)	0.078
Hip LoS Men	-0.007 (-0.047,0.033)	0.732	0.347 (-0.44,1.135)	0.387	-0.02 (-0.073,0.033)	0.458	0.293 (-0.117,0.704)	0.161	0.289 (-0.193,0.771)	0.239	0.458 (-0.163,1.079)	0.149
Hip LoS Women	-0.004 (-0.035,0.028)	0.816	0.235 (-0.271,0.741)	0.362	-0.014 (-0.046,0.018)	0.391	0.693 (0.395,0.991)	0.000	0.009 (-0.421,0.439)	0.966	-0.203 (-0.762,0.357)	0.477
Hip LoS Charlson 0	-0.011 (-0.039,0.016)	0.418	0.297 (-0.242,0.837)	0.280	-0.005 (-0.035,0.025)	0.738	0.912 (0.617,1.208)	0.000	0.178 (-0.123,0.478)	0.247	0.222 (-0.066,0.51)	0.131
Hip LoS Charlson 1	0.034 (0.003,0.065)	0.030	0.238 (-0.604,1.08)	0.580	-0.077 (-0.137,-0.018)	0.011	0.191 (-0.385,0.767)	0.515	0.312 (-0.431,1.056)	0.410	0.014 (-0.563,0.591)	0.962
Hip LoS Charlson 2+	-0.123 (-0.239,-0.007)	0.038	0.172 (-1.97,2.314)	0.875	0.116 (0.005,0.228)	0.040	-0.789 (-2.189,0.611)	0.269	-0.783 (-2.119,0.553)	0.250	0.188 (-2.176,2.552)	0.876
Hip LoS Dep 1	0.004 (-0.029,0.037)	0.814	0.553 (-0.303,1.408)	0.206	-0.017 (-0.065,0.032)	0.501	0.784 (0.131,1.437)	0.019	0.42 (-0.034,0.874)	0.070	0.299 (-0.401,0.999)	0.402
Hip LoS Dep 2	-0.038 (-0.083,0.006)	0.092	0.822 (-0.167,1.81)	0.103	-0.015 (-0.078,0.047)	0.630	1.509 (0.737,2.28)	0.000	0.66 (-0.202,1.522)	0.133	0.589 (-0.461,1.638)	0.272
Hip LoS Dep 3	-0.034 (-0.102,0.033)	0.319	0.617 (-0.789,2.023)	0.390	0.01 (-0.072,0.091)	0.819	-0.216 (-1.011,0.579)	0.595	-0.424 (-1.293,0.445)	0.339	-0.701 (-1.351,-0.05)	0.035
Hip LoS Dep 4	0.081 (0.017,0.144)	0.013	-0.473 (-1.858,0.913)	0.504	-0.112 (-0.189,-0.035)	0.004	-0.374 (-1.59,0.842)	0.547	-1.204 (-2.355,-0.053)	0.040	-0.491 (-1.989,1.007)	0.520
Hip LoS Dep 5	0.015 (-0.056,0.085)	0.682	-0.72 (-1.647,0.208)	0.128	0 (-0.07,0.07)	0.998	0.621 (-0.104,1.347)	0.093	0.689 (-0.25,1.629)	0.150	0.816 (-0.696,2.329)	0.290
Hip Bed Occ	0.997 (0.987,1.008)	0.627	1 (0.862,1.161)	0.996	0.987 (0.97,1.004)	0.128	1.291 (1.116,1.492)	0.001	1.283 (1.14,1.444)	0.000	1.146 (0.99,1.326)	0.068
Hip Public Private	0.013 (-0.015,0.041)	0.377	-0.741 (-1.237,-0.245)	0.003	-0.019 (-0.05,0.011)	0.218	-0.008 (-0.212,0.196)	0.939	0.308 (0.154,0.463)	0.000	0.038 (-0.166,0.241)	0.718
Knee Admissions	0.995 (0.992,0.999)	0.016	0.843 (0.761,0.934)	0.001	1.005 (0.998,1.012)	0.170	1.308 (1.154,1.482)	0.000	1.26 (1.16,1.369)	0.000	1.286 (1.178,1.403)	0.000
Knee Average Age	-0.078 (-0.152,-0.005)	0.037	-1.632 (-2.825,-0.439)	0.007	0.211 (0.128,0.293)	0.000	0.926 (-0.072,1.924)	0.069	0.953 (0.077,1.829)	0.033	0.354 (-0.422,1.13)	0.371
Knee Prop Women	1.004 (0.999,1.009)	0.086	0.96 (0.861,1.071)	0.465	0.994 (0.987,1.001)	0.097	1.037 (0.973,1.105)	0.260	1.017 (0.922,1.121)	0.743	1.036 (0.966,1.111)	0.319
Knee Prop 2+ Charlson	1.009 (0.993,1.026)	0.250	0.638 (0.468,0.869)	0.004	1.042 (1.021,1.064)	0.000	1.156 (0.931,1.436)	0.190	1.074 (0.841,1.372)	0.567	0.909 (0.626,1.321)	0.617
Knee Prop High Deprivation	1.005 (0.999,1.011)	0.107	0.968 (0.835,1.122)	0.664	0.986 (0.977,0.995)	0.003	1.224 (1.082,1.384)	0.001	1.075 (0.95,1.217)	0.251	1.031 (0.924,1.149)	0.586
Knee LoS	-0.024 (-0.047,-0.001)	0.042	0.176 (-0.2,0.551)	0.360	-0.008 (-0.031,0.015)	0.490	0.422 (0.033,0.81)	0.033	0.15 (-0.199,0.499)	0.398	0.396 (-0.011,0.803)	0.057

For peer review only

Knee LoS Age 16-59	-0.016 (-0.032,0.001)	0.068	0.403 (0.008,0.799)	0.046	-0.028 (-0.059,0.003)	0.081	0.484 (0.198,0.77)	0.001	0.422 (-0.01,0.855)	0.056	0.475 (0.216,0.734)	0.000
Knee LoS Age 16-59	0.007 (-0.018,0.033)	0.571	-0.295 (-0.854,0.265)	0.302	-0.026 (-0.062,0.009)	0.147	0.069 (-0.455,0.593)	0.795	-0.194 (-0.652,0.265)	0.407	-0.096 (-0.713,0.52)	0.759
Knee LoS Age 70-79	-0.009 (-0.045,0.027)	0.616	0.77 (0.101,1.439)	0.024	-0.054 (-0.096,-0.012)	0.012	-0.086 (-0.736,0.563)	0.794	-0.552 (-1.116,0.012)	0.055	0.367 (-0.3,1.034)	0.281
Knee LoS Age 80+	-0.051 (-0.097,-0.006)	0.026	-0.562 (-1.849,0.724)	0.391	0.028 (-0.062,0.119)	0.541	1.521 (0.119,2.923)	0.034	1.37 (0.391,2.349)	0.006	1.361 (0.617,2.106)	0.000
Knee LoS Men	-0.007 (-0.028,0.014)	0.514	0.096 (-0.297,0.488)	0.633	-0.033 (-0.062,-0.004)	0.024	0.082 (-0.416,0.58)	0.746	0.157 (-0.244,0.558)	0.444	0.205 (-0.149,0.559)	0.256
Knee LoS Women	-0.042 (-0.079,-0.005)	0.027	0.252 (-0.356,0.861)	0.416	0.017 (-0.018,0.052)	0.350	0.635 (-0.036,1.307)	0.064	0.15 (-0.454,0.754)	0.625	0.49 (-0.258,1.239)	0.199
Knee LoS Charlson 0	-0.021 (-0.044,0.002)	0.077	0.067 (-0.312,0.446)	0.730	-0.011 (-0.036,0.015)	0.419	0.645 (0.331,0.959)	0.000	0.193 (-0.129,0.516)	0.240	0.415 (0.075,0.754)	0.017
Knee LoS Charlson 1	-0.03 (-0.064,0.005)	0.092	0.47 (-0.199,1.139)	0.169	-0.029 (-0.067,0.01)	0.145	0.455 (-0.097,1.007)	0.106	0.324 (-0.341,0.988)	0.340	1.058 (0.384,1.731)	0.002
Knee LoS Charlson 2+	-0.04 (-0.092,0.012)	0.134	0.407 (-1.956,2.771)	0.736	-0.022 (-0.184,0.14)	0.789	-0.627 (-1.625,0.37)	0.218	-0.949 (-2.374,0.476)	0.192	-1.433 (-2.175,-0.691)	0.000
Knee LoS Dep 1	-0.009 (-0.05,0.031)	0.644	-0.035 (-0.656,0.586)	0.911	-0.008 (-0.059,0.043)	0.750	0.257 (-0.541,1.056)	0.527	0.096 (-0.581,0.772)	0.782	-0.127 (-0.724,0.47)	0.677
Knee LoS Dep 2	-0.019 (-0.055,0.017)	0.300	0.199 (-0.57,0.968)	0.612	-0.043 (-0.086,0)	0.049	0.018 (-0.731,0.767)	0.962	-0.731 (-1.507,0.046)	0.065	0.05 (-0.887,0.987)	0.917
Knee LoS Dep 3	0.014 (-0.031,0.059)	0.544	-0.426 (-1.102,0.25)	0.217	-0.036 (-0.095,0.022)	0.220	0.976 (0.281,1.672)	0.006	0.909 (-0.067,1.886)	0.068	0.547 (-0.12,1.215)	0.108
Knee LoS Dep 4	-0.064 (-0.127,-0.001)	0.047	0.634 (-0.165,1.432)	0.120	0.033 (-0.04,0.106)	0.380	0.669 (-0.076,1.414)	0.079	0.617 (0.007,1.227)	0.047	0.852 (-0.274,1.978)	0.138
Knee LoS Dep 5	-0.035 (-0.087,0.018)	0.194	0.709 (-0.225,1.643)	0.137	0.015 (-0.042,0.072)	0.606	0.123 (-0.701,0.947)	0.770	-0.387 (-1.304,0.531)	0.409	1.104 (0.188,2.02)	0.018
Knee Bed Occ	0.993 (0.986,0.999)	0.032	0.834 (0.728,0.956)	0.009	1 (0.991,1.009)	0.991	1.42 (1.308,1.542)	0.000	1.373 (1.213,1.553)	0.000	1.465 (1.318,1.627)	0.000
Knee Public Private	0.006 (-0.02,0.031)	0.667	-0.476 (-1.026,0.074)	0.090	-0.015 (-0.04,0.009)	0.225	0.113 (-0.089,0.314)	0.274	0.276 (0.035,0.517)	0.025	0.131 (-0.071,0.332)	0.205
Elec Emerg Ratio	-0.005 (-0.013,0.003)	0.198	-0.322 (-0.455,-0.189)	0.000	-0.016 (-0.026,-0.005)	0.004	-0.008 (-0.107,0.091)	0.872	-0.053 (-0.18,0.074)	0.414	-0.028 (-0.163,0.108)	0.690

For peer review only

The RECORD statement – checklist of items, extended from the STROBE statement, that should be reported in observational studies using routinely collected health data.

	Item No.	STROBE items	Location in manuscript where items are reported	RECORD items	Location in manuscript where items are reported
**Title and abstract**					
	1	(a) Indicate the study's design with a commonly used term in the title or the abstract (b) Provide in the abstract an informative and balanced summary of what was done and what was found		RECORD 1.1: The type of data used should be specified in the title or abstract. When possible, the name of the databases used should be included.<br><br>RECORD 1.2: If applicable, the geographic region and timeframe within which the study took place should be reported in the title or abstract.<br><br>RECORD 1.3: If linkage between databases was conducted for the study, this should be clearly stated in the title or abstract.	Abstract P2 L35-37<br><br>Abstract P2 L35-38
**Introduction**					
Background rationale	2	Explain the scientific background and rationale for the investigation being reported			Introduction P4-5 L76-113
Objectives	3	State specific objectives, including any prespecified hypotheses			Introduction P5 L107-113
**Methods**					
Study Design	4	Present key elements of study design early in the paper			Methods P5 L116-119
Setting	5	Describe the setting, locations, and relevant dates, including periods of recruitment, exposure, follow-up, and data collection			Methods P5 L116-119 Methods P7-8 L70-176

For peer review only

Participants	6	*(a) Cohort study* - Give the eligibility criteria, and the sources and methods of selection of participants. Describe methods of follow-up<br>*Case-control study* - Give the eligibility criteria, and the sources and methods of case ascertainment and control selection. Give the rationale for the choice of cases and controls<br>*Cross-sectional study* - Give the eligibility criteria, and the sources and methods of selection of participants<br><br>*(b) Cohort study* - For matched studies, give matching criteria and number of exposed and unexposed<br>*Case-control study* - For matched studies, give matching criteria and the number of controls per case		RECORD 6.1: The methods of study population selection (such as codes or algorithms used to identify subjects) should be listed in detail. If this is not possible, an explanation should be provided.<br><br>RECORD 6.2: Any validation studies of the codes or algorithms used to select the population should be referenced. If validation was conducted for this study and not published elsewhere, detailed methods and results should be provided.<br><br>RECORD 6.3: If the study involved linkage of databases, consider use of a flow diagram or other graphical display to demonstrate the data linkage process, including the number of individuals with linked data at each stage.	Methods P6 L141-147 Supplementary Table T1
Variables	7	Clearly define all outcomes, exposures, predictors, potential confounders, and effect modifiers. Give diagnostic criteria, if applicable.		RECORD 7.1: A complete list of codes and algorithms used to classify exposures, outcomes, confounders, and effect modifiers should be provided. If these cannot be reported, an explanation should be provided.	Methods P6-7 L141-169
Data sources/ measurement	8	For each variable of interest, give sources of data and details of methods of assessment (measurement).<br>Describe comparability of assessment methods if there is more than one group			Methods P5-6 L120-140

For peer review only

Bias	9	Describe any efforts to address potential sources of bias			Methods P7-8 L170-185,
Study size	10	Explain how the study size was arrived at			Methods P6 L141-147
Quantitative variables	11	Explain how quantitative variables were handled in the analyses. If applicable, describe which groupings were chosen, and why			Methods P6-7 L141-169
Statistical methods	12	(a) Describe all statistical methods, including those used to control for confounding<br>(b) Describe any methods used to examine subgroups and interactions<br>(c) Explain how missing data were addressed<br>(d) *Cohort study* - If applicable, explain how loss to follow-up was addressed<br>*Case-control study* - If applicable, explain how matching of cases and controls was addressed<br>*Cross-sectional study* - If applicable, describe analytical methods taking account of sampling strategy<br>(e) Describe any sensitivity analyses			Methods P7-8 L170-185
Data access and cleaning methods		..		RECORD 12.1: Authors should describe the extent to which the investigators had access to the database population used to create the study population.	Data Sharing P17 L392-400

For peer review only

				RECORD 12.2: Authors should provide information on the data cleaning methods used in the study.	Methods P6-7 L141-169
Linkage		..		RECORD 12.3: State whether the study included person-level, institutional-level, or other data linkage across two or more databases. The methods of linkage and methods of linkage quality evaluation should be provided.	N/A
**Results**					
Participants	13	(a) Report the numbers of individuals at each stage of the study (*e.g.*, numbers potentially eligible, examined for eligibility, confirmed eligible, included in the study, completing follow-up, and analysed)<br>(b) Give reasons for non-participation at each stage.<br>(c) Consider use of a flow diagram		RECORD 13.1: Describe in detail the selection of the persons included in the study (*i.e.,* study population selection) including filtering based on data quality, data availability and linkage. The selection of included persons can be described in the text and/or by means of the study flow diagram.	Methods P6 L141-147<br><br>Results P8 L191-194
Descriptive data	14	(a) Give characteristics of study participants (*e.g.*, demographic, clinical, social) and information on exposures and potential confounders<br>(b) Indicate the number of participants with missing data for each variable of interest<br>(c) *Cohort study* - summarise follow-up time (*e.g.*, average and total amount)			Results P8 L191-194
Outcome data	15	*Cohort study* - Report numbers of outcome events or summary measures over time<br>*Case-control study* - Report numbers in each exposure			Results P8-9 L195-206

For peer review only

		category, or summary measures of exposure<br>*Cross-sectional study* - Report numbers of outcome events or summary measures			
Main results	16	(a) Give unadjusted estimates and, if applicable, confounder-adjusted estimates and their precision (e.g., 95% confidence interval). Make clear which confounders were adjusted for and why they were included<br>(b) Report category boundaries when continuous variables were categorized<br>(c) If relevant, consider translating estimates of relative risk into absolute risk for a meaningful time period			Results P9-11 L196-267
Other analyses	17	Report other analyses done—e.g., analyses of subgroups and interactions, and sensitivity analyses			Results P9-11 L196-267
**Discussion**					
Key results	18	Summarise key results with reference to study objectives			Discussion P11-12, L268-288
Limitations	19	Discuss limitations of the study, taking into account sources of potential bias or imprecision. Discuss both direction and magnitude of any potential bias		RECORD 19.1: Discuss the implications of using data that were not created or collected to answer the specific research question(s). Include discussion of misclassification bias, unmeasured confounding, missing data, and changing eligibility over time, as they pertain to the study being reported.	Discussion P12-13 L289-303
Interpretation	20	Give a cautious overall interpretation of results considering objectives,			Discussion P13-14 L317-340

For peer review only

		limitations, multiplicity of analyses, results from similar studies, and other relevant evidence			
Generalisability	21	Discuss the generalisability (external validity) of the study results			Discussion P13 L294-303
**Other Information**					
Funding	22	Give the source of funding and the role of the funders for the present study and, if applicable, for the original study on which the present article is based			Funding Statement P16 L375-380
Accessibility of protocol, raw data, and programming code		..		RECORD 22.1: Authors should provide information on how to access any supplemental information such as the study protocol, raw data, or programming code.	Methods P8 L186-188 Data Sharing P17 L392-400

*Reference: Benchimol EI, Smeeth L, Guttmann A, Harron K, Moher D, Petersen I, Sørensen HT, von Elm E, Langan SM, the RECORD Working Committee. The REporting of studies Conducted using Observational Routinely-collected health Data (RECORD) Statement. *PLoS Medicine* 2015; in press.

*Checklist is protected under Creative Commons Attribution (CC BY) license.

For peer review only
